# Genomic Insights into the Distribution and Phylogeny of Glycopeptide Resistance Determinants within the *Actinobacteria* Phylum

**DOI:** 10.3390/antibiotics10121533

**Published:** 2021-12-14

**Authors:** Andrés Andreo-Vidal, Elisa Binda, Victor Fedorenko, Flavia Marinelli, Oleksandr Yushchuk

**Affiliations:** 1 Department of Biotechnology and Life Sciences, University of Insubria, 21100 Varese, Italy; aandreovidal@uninsubria.it (A.A.-V.); elisa.binda@uninsubria.it (E.B.); oleksandr.yushchuk@uninsubria.it (O.Y.); 2 Department of Genetics and Biotechnology, Ivan Franko National University of Lviv, 79005 Lviv, Ukraine; viktor.fedorenko@lnu.edu.ua

**Keywords:** glycopeptide antibiotics, multidrug resistance, antibiotic resistance, *van* genes

## Abstract

The spread of antimicrobial resistance (AMR) creates a challenge for global health security, rendering many previously successful classes of antibiotics useless. Unfortunately, this also includes glycopeptide antibiotics (GPAs), such as vancomycin and teicoplanin, which are currently being considered last-resort drugs. Emerging resistance towards GPAs risks limiting the clinical use of this class of antibiotics—our ultimate line of defense against multidrug-resistant (MDR) Gram-positive pathogens. But where does this resistance come from? It is widely recognized that the GPA resistance determinants—*van* genes—might have originated from GPA producers, such as soil-dwelling Gram-positive actinobacteria, that use them for self-protection. In the current work, we present a comprehensive bioinformatics study on the distribution and phylogeny of GPA resistance determinants within the *Actinobacteria* phylum. Interestingly, *van*-like genes (*vlgs*) were found distributed in different arrangements not only among GPA-producing actinobacteria but also in the non-producers: more than 10% of the screened actinobacterial genomes contained one or multiple *vlgs*, while less than 1% encoded for a biosynthetic gene cluster (BGC). By phylogenetic reconstructions, our results highlight the co-evolution of the different *vlgs*, indicating that the most diffused are the ones coding for putative VanY carboxypeptidases, which can be found alone in the genomes or associated with a *vanS*/*R* regulatory pair.

## 1. Introduction

Starting with the discovery of penicillin [1], humanity has been involved in a never-ending arms race between life-threatening bacterial pathogens and antibiotics, either natural, semisynthetic or completely synthetic. An estimation made by the United Nations Interagency Coordination Group on Antimicrobial Resistance in 2014 predicted that the spread of antimicrobial resistance (AMR) will cause up to 10 million deaths per year by 2050 [2]. However, recent events might make this number even more grim: the worldwide health crisis caused by SARS-CoV-2 has led to an increase in antibiotic use and misuse, which, in turn, is likely to further accelerate AMR diffusion [3]. AMR has rendered many previously successful groups of antibiotics non-functional, leaving us hiding behind the “thin red line” of last-resort drugs, capable to combat multidrug-resistant (MDR) pathogens. Glycopeptide antibiotics (GPAs) are a class of non-ribosomally synthesized, highly cross-linked, halogenated and glycosylated natural products, which are considered frontline drugs against Gram-positive MDR pathogens such as *Staphylococcus aureus*, *Enterococcus* spp., *Clostridioides difficile*, etc. [4].

**Figure 1:**
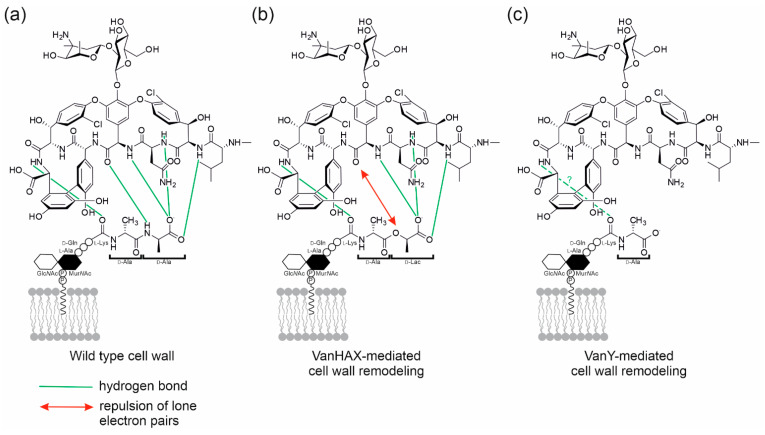
Schematic representation of (**a**) vancomycin (as a model GPA), interacting with the d-Ala-d-Ala terminus of the lipid II pentapeptide stem of a Gram-positive cell wall, forming five hydrogen bonds [10]; (**b**) in the cell wall remodeled by the action of VanHAX, the d-Ala-d-Lac termini of lipid II pentapeptide stem interacts with a significantly lower (1000 fold lower) affinity with vancomycin, due to the formation of only four hydrogen bonds and the repulsion of lone electron pairs between oxygen atoms [11]; (**c**) in the cell wall remodeled by the action of VanY d,d-carboxypeptidase, the lipid II pentapeptide stem is truncated by the excision of the terminal d-Ala, and vancomycin affinity for this target appears significantly reduced, although to which extent has not been investigated yet.

GPAs are produced by high G-C content soil-dwelling actinobacteria and could be divided into five types according to their chemical structures and molecular targets [5]. Types I–IV are compounds having a cross-linked heptapeptide core highly glycosylated and/or lipidated; their molecular target in pathogens is lipid II [6], a highly conserved macromolecule among bacteria that is essential for cell-wall biosynthesis [7]. Types I–IV GPAs form five hydrogen bonds with the d-alanyl-d-alanine (d-Ala-d-Ala) terminus of the lipid II pentapeptide stem (Figure 1a) [8,9,10,11]. Such binding terminates upstream transpeptidation and transglycosylation reactions, preventing the formation of mature peptidoglycan (PG). An old term describes Types I–IV GPAs very precisely: dalbaheptides [12], meaning d-Ala-d-Ala-binding antibiotics with heptapeptide structures. Lipid II is obviously involved in the cell wall biosynthesis of dalbaheptide producers as well. Thus, to avoid suicide during GPA production, producing strains need self-resistance mechanisms. This topic was recently revised in detail [13,14]. In brief, two main mechanisms of cell-wall remodeling exist in dalbaheptide-producers as well as in the resistant pathogens [15,16]. The first involves three genes—*vanHAX—*coding for: (i) an α-ketoacid dehydrogenase, VanH, which stereospecifically reduces pyruvate to d-lactate (d-Lac); (ii) a d-Ala-d-Lac ligase, VanA; and (iii) a d,d-dipeptidase, VanX. VanX removes the d-Ala-d-Ala termini of lipid II pentapeptide stems, while VanA prepares a pool of d-Ala-d-Lac dipeptides, which MurF, a UDP-*N*-acetylmuramoyl-tripeptide ligase, installs instead of the terminal d-Ala-d-Ala. As a result, GPAs form four instead of five hydrogen bonds with such d-Ala-d-Lac termini, and the repulsion of lone electron pairs between oxygen atoms contributes to make these GPA-lipid II complexes unstable (Figure 1b). The second resistance mechanism requires the expression of a d,d-carboxypeptidase VanY, which cleaves the terminal d-Ala residues of lipid II pentapeptide stems, hampering GPA complex formation with such truncated lipid II derivatives (Figure 1c). So, the formed tetrapeptide-carrying lipid II molecules are still able to enter the transpeptidation and transglycosylation steps, yielding a 3-3 crosslinked mature peptidoglycan [17]. Expression of either *vanHAX* or *vanY* (known overall as *van* genes) is often inducible and regulated by a VanRS two-component regulatory pair. There, a sensor histidine kinase VanS (de)phosphorylates transcriptional regulator VanR in response to the extracellular presence of GPAs, and phosphorylated VanR activates the expression of the *van* genes involved in cell-wall remodeling. In dalbaheptide producers, *van* genes are always localized inside the borders of GPA biosynthetic gene clusters (BGCs), which group genes devoted to antibiotic biosynthesis, transport and regulation, guaranteeing co-regulation of self-resistance with antibiotic production. The only exception is the chloroeremomycin BGC in *Kibdelosporangium aridum* A82846, which apparently does not include *van* genes [18].

Type V GPAs are not glycopeptides *sensu stricto*, since they are not glycosylated; moreover, they are not dalbaheptides, since they include also nonapeptide antibiotics (such as corbomycin and GP6738 [19,20]), and they target autolysins (named also murein hydrolases) instead of lipid II. Autolysins are enzymes breaking the bonds within the peptidoglycan to allow bacterial growth and cell division [21]; thus, type V GPAs block cell-wall remodeling, arresting cell division. Consistently, type V GPA BGCs lack the cluster-situated *van* genes needed to remodel lipid II termini [19,22]. *van* genes are also missed in the related BGC of the uncrosslinked non-glycosylated peptide antibiotic known as feglymycin [23], which inhibits peptidoglycan synthesis targeting MurA (the UDP-*N*-acetylglucosamine 1-carboxyvinyltransferase, catalyzing phosphoenolpyruvate transfer to UDP-*N*-acetylglucosamine) and MurB (the UDP-*N*-acetylenolpyruvoylglucosamine reductase, catalyzing the last step of the formation of UDP-*N*-acetylmuramic acid), both acting at the cytoplasmic step of cell-wall biosynthesis. However, type V GPA and feglymycin BGCs carry genes for a two-component regulatory pair, consisting of a sensor histidine kinase and a response regulator, which remind VanS and VanR of dalbaheptide BGCs, but whose functions remains unclear.

Intriguingly, functional *van* genes are also found in non-producing actinobacteria such as in *Streptomyces coelicolor* A3(2) [24], as well as in various other environmental non-infectious low G-C content bacteria (such as *Paenibacillus popilliae* [25] or *Bacillus circulans* [26]). As a consequence, different hypotheses have been proposed to explain *van* gene distribution and evolution in bacteria. The most accredited one is that pathogens such as enterococci (and the GPA non-producing environmental bacteria) might have acquired *van* genes from dalbaheptide producers through a series of horizontal gene transfer events, likely promoted by the selective pressure exerted by antibiotic environmental contamination [27]. An alternative hypothesis suggests that pathogens received *van* genes from low G-C soil Gram-positives (*Firmicutes* phylum), as the above-mentioned *Pnb. popilliae* and *Bac. circulans* [28], implying that in these bacteria *van* genes evolved independently.

To get an insight into the phylogeny of *van* genes, in this work we analyze their distribution and arrangement within the different orders belonging to the *Actinobacteria* phylum, using the genomic data available in public databases. We have investigated more than 7000 actinobacterial genomes and found *van-*like genes (defined hereafter *vlgs*) in more than one tenth of them. Thus, we can confirm that the presence of *vlgs* is not limited to the dalbaheptide producers, since these genes are also widely distributed among genomes of Type V GPA producers and in non-producing actinobacterial taxa, which do not need them for self-resistance. In addition, phylogenetic reconstructions made for VanY-like proteins as well as for the VanHAX triads and for the two-component VanS/VanR regulatory system, highlight the evolutionary independent stories of the corresponding gene acquisitions. Thanks to this comparative genomic analysis, novel transposon-like mobile elements carrying *vlgs* are here for the first time described, originating from poorly investigated orders such as *Eggerthellales* and *Coriobacteriales.* Finally, as a control, the same bioinformatic analysis was applied to more than 2000 *Bacillales* complete genome assemblies, which yielded only a few *vlgs*, often adjacent to transposase-like open reading frames (ORFs). Taken altogether, these data reveal that the phylum *Actinobacteria* is an incredibly vast source of variable GPA resistance determinants, which might potentially continue to move towards pathogens, contributing to the alarming diffusion of AMR. Their study might help the surveillance of AMR spread in compliance with the One-Health approach [29].

## 2. Results

### 2.1. Organization of vlgs in GPA Producers and Beyond

Until now, *vlgs* in actinobacteria were reported as BGC-situated in more than 20 “classical” dalbaheptide-producers [13]. The case of the GPA non-producer actinobacterium *S. coelicolor* A3(2)—having a whole set of *vlgs* [30,31]—was rather interpreted as an exception. Thanks to the abundance of genomic data on actinobacteria today, our aim is to prove or disprove the assumption that *vlgs* are peculiar to GPA producers, and to clarify how these genes are eventually distributed and organized among different orders belonging to *Actinobacteria* phylum. Thus, we screened all the genome assemblies available for actinobacteria in GenBank at the moment of this work preparation (April 2020, Supplementary Excel File S1). This search covered 28 orders of the *Actinobacteria* phylum (Table 1), including two “candidate” ones (namely *candidatus* Actinomarinales and Nanopelagicales). We searched for *vanY* and *vanHAX* sequences, co-localized with *vanRS-*like two-component regulatory pairs, and then we analyzed the genetic context of these genes.

At least one *vlg* sequence was found in the majority of orders (22, Table 1); only the orders *Acidimicrobiales, candidatus* Actinomarinales*, Actinopolysporales, Bifidobacteriales, Egibacteriales* and *Gaiellales* lacked any *vlgs*. *vlgs* were most abundant (found in more than 10% of the genomic records per each order) in orders *Catenulisporales, Corynebacteriales, Cryptosporangiales, Frankiales, Geodermatophilales, Glycomycetales, Jiangellales, Micromonosporales, Nakamurellales, Nitriliruptorales, Pseudonocardiales, Rubrobacterales, Streptomycetales* and *Streptosporangiales* (Table 1, Supplementary Excel File S2). Since the known GPA producers belong to *Pseudonocardiales, Streptosporangiales, Micromonosporales* and *Streptomycetales* [5], we started to investigate *vlgs* organization in these orders in detail, to move then into the analysis of the still unexplored taxa.

#### 2.1.1. Order *Pseudonocardiales*

Order *Pseudonocardiales* is the most abundant source of Types I–IV GPAs [5,32]. So far, GPA BGCs were described only in the *Amycolatopsis* and *Kibdelosporangium* genera. In our screening of 243 genomes from *Pseudonocardiales* spp., we found at least one *vlgs* sequence in 135 assemblies (Table 1, Supplementary Excel Files S1 and S2). Only 30 assemblies contained GPA BGCs (Supplementary Excel File S2). Besides the known GPA producing genera, *Amycolatopsis* and *Kibdelosporangium* [18], a GPA BGC was, for the first time, found in a species belonging to the genus *Actinokineospora.* Overall, no correlation between the quality and quantity of *vlgs* in GPA producers and non-producers was observed. The repertoire of *vlgs* was quite different in each strain. The following combinations were found: *vanYRS, vanHAXRS, vanHAX, vanYHAX* and *vanYHAXRS.* A significant portion of the *vanY-*like genes was found to be “orphan” (meaning not co-localized with any other *vlgs*). Sequences similar to the *vanJ* and *vanZ* genes, which were previously sporadically reported as involved in GPA resistance, but apparently without an essential role [14], were rare and always co-localized with the *vanRS*-like pair (except cases in *Pseudonocardia* sp. CNS-139—*vanHAXZ* arrangement; and in *Pseudonocardia cypriaca* DSM 45511—*vanHAXJ* arrangement).

**Figure 2:**
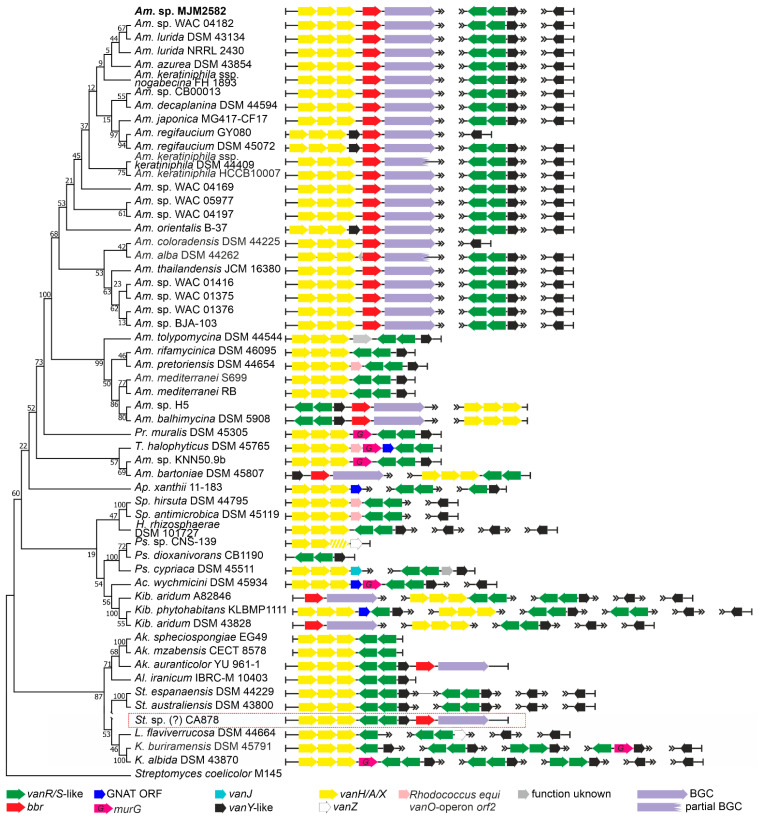
Organization of *vlgs* found within the set of 55 chosen genomes from *Pseudonocardiales* spp. Maximum Likelihood phylogenetic tree of the 16S rRNA genes of the corresponding species (see Section 4 for details) served as a phylogenetic framework for the scheme. *Amycolatopsis* sp. MJM2582 (in bold at the top of the figure) is outside the framework due to the lack of a full 16S rRNA gene in the corresponding genome assembly. *vlgs* from the metagenomic CA878 BGC (highlighted with red frame) were arbitrary introduced in this scheme since the retrieved sequences likely belong to *Saccharothrix* spp. Names of the *Pseudonocardiales* genera were abbreviated according to ESM Table S1. The legend below the figure explains the color-coding of the scheme. Please refer to the text for the role of the single genes. Pseudogenes are shaded.

To study the arrangement of *vlgs* in detail, we focused on the 30 genomes of the known GPA producers and, as a control, on 25 genomes of never previously investigated non-producing *Pseudonocardiales* spp. (Figure 2). Thus, in the majority of the GPA-producing *Amycolatopsis*, the *vanHAX* operon was found just upstream of the *bbr*-orthologue (coding for a StrR-like cluster-situated-pathway-specific regulator of GPA biosynthesis [33]), in rare cases having a *vanY*-like gene in between (Figure 2). At the same time, genomes of these *Amycolatopsis* GPA producers contained two copies of *vanY-*like genes located outside the GPA BGCs (Figure 2). One of these copies was always co-localized with *vanRS-*like two-component regulatory genes. *Amycolatopsis balhimycina* DSM 5908 (balhimycin producer) and *Amycolatopsis* sp. H5 were notable exceptions: belonging to a different clade than other *Amycolatopsis* spp. GPA producers, they had a *vanSRY*-genes cluster-situated and a *vanHAX* operon outside the BGC (Figure 2). In *Amycolatopsis bartoniae* DSM 45807, only a *vanY-*like gene was found upstream of the *bbr*-orthologue, while a *vanHAX* operon coupled with *vanRS-*like two-component regulatory genes was placed somewhere else on the chromosome (Figure 2). However, according to 16S rRNA gene phylogeny (see phylogenetic framework on Figure 2), *Am. bartoniae* appeared to outgroup all other *Amycolatopsis* spp. together with *Prauserella muralis* DSM 45305, *Tamaricihabitans halophyticus* DSM 45765 and *Amycolatopsis* sp. KNN50.9b. Thus, it is likely that *Am. bartoniae* (as well as *Amycolatopsis* sp. KNN50.9b) might not belong to the *Amycolatopsis* genus at all. GPA-producing *Kibdelosporangium* spp. had no cluster-situated *vlgs,* but *vanHAX* operons and multiple copies of *vanY-*like genes were found somewhere else on the chromosome (Figure 2). Finally, in *Actinokineospora auranticolor* YU 961-1, *vlgs* were only GPA cluster-situated—a set of *vanHAXRSY* genes was found upstream of the *bbr*-orthologue (Figure 2).

Notably, in some cases (e.g., *Saccharopolyspora hirsuta* DSM 44795), the *vanHAX* operon was followed by a homologue of *orf2* (a gene coding for a protein with unknown function) previously identified in the *Rhodococcus equi* S7B *vanO* operon [34]. As it does occur in the *vanO* operon, the *orf2* homologue was in some species followed by a *murG-*like gene (e.g., *T. halophyticus* DSM 45765), coding for an essential peptidoglycan glycosyltransferase [35]. It is so far unknown how exactly MurG contributes to GPA resistance, but it might be assisting *van-*mediated cell-wall remodeling. In a few species, such as *Pr. muralis* DSM 45305, *murG* was following *vanHAX* directly. Genes coding for a GCN5-related *N*-acetyltransferases (GNATs) were also often found co-localized with *Pseudonocardiales* spp. *vlgs*.

Finally, we introduced in our phylogenetic framework the only metagenomics-derived GPA BGC—CA878 [36]—which was recently shown to be rather closely related to BGCs from *Amycolatopsis* spp. [18]. We speculated that this BGC might also belong to some unknown species of *Pseudonocardiales*—the organization of cluster-situated *vlgs* here seemed identical to the one from *Ak. auranticolor* YU 961-1. Fortunately, at the moment of its discovery, CA878 BGC was sequenced together with unannotated DNA flanks (*ca.* 26 and 3 kbp). This allowed us to annotate 9 ORFs upstream and 6 ORFs downstream of the borders of CA878 BGC (ESM Figure S1). It appeared that the vast majority of these ORFs coded for proteins with orthologues in *Saccharothrix* spp. (ESM Figure S1). In our opinion, it is possible that CA878 comes from an unknown species belonging to the *Saccharothrix* genus, further expanding the list of GPA-producing *Pseudonocardiales* genera.

#### 2.1.2. Order *Streptosporangiales*

Order *Streptosporangiales* is the source of valuable lipidated dalbaheptides, such as A40926 from *Nonomuraea gerenzanensis* ATCC 39727, which is the precursor of second-generation dalbavancin [4,37]. In addition*, N. gerenzanensis* was the first model for studying the role of VanY-like carboxypeptidases in self-resistance [38,39]. Other known GPA producers are *Nonomuraea coxensis* DSM 45129, recently reported to produce the lipoglycopeptide A50926 [40], and *Nonomuraea* sp. ATCC 5507, which produces the type V kistamicin [41]. Here, we analyzed the genomic records from 228 *Streptosporangiales* spp. (Supplementary Excel File S1). *vlgs* were found in 63 out of them (Table 1, Supplementary Excel File S2). *vanY-*like genes were the most abundant, in almost all cases being co-localized with *vanRS*-like regulatory pairs (although few “orphan” ones were also found). *vanHAX* operons were not found so often, coming exclusively from *Actinomadura* spp.; anyhow, the *vanHAX* operon was never found associated with *vanY*-like genes. Accessory *vlgs*, such as *vanJ,* were found rarely and tended to be co-localized with the *vanRS*-like regulatory pairs. Only in one case—in *Actinomadura* sp. H3C3—a *vanZ* pseudogene was discovered, co-localized with *vanRSY*.

**Figure 3:**
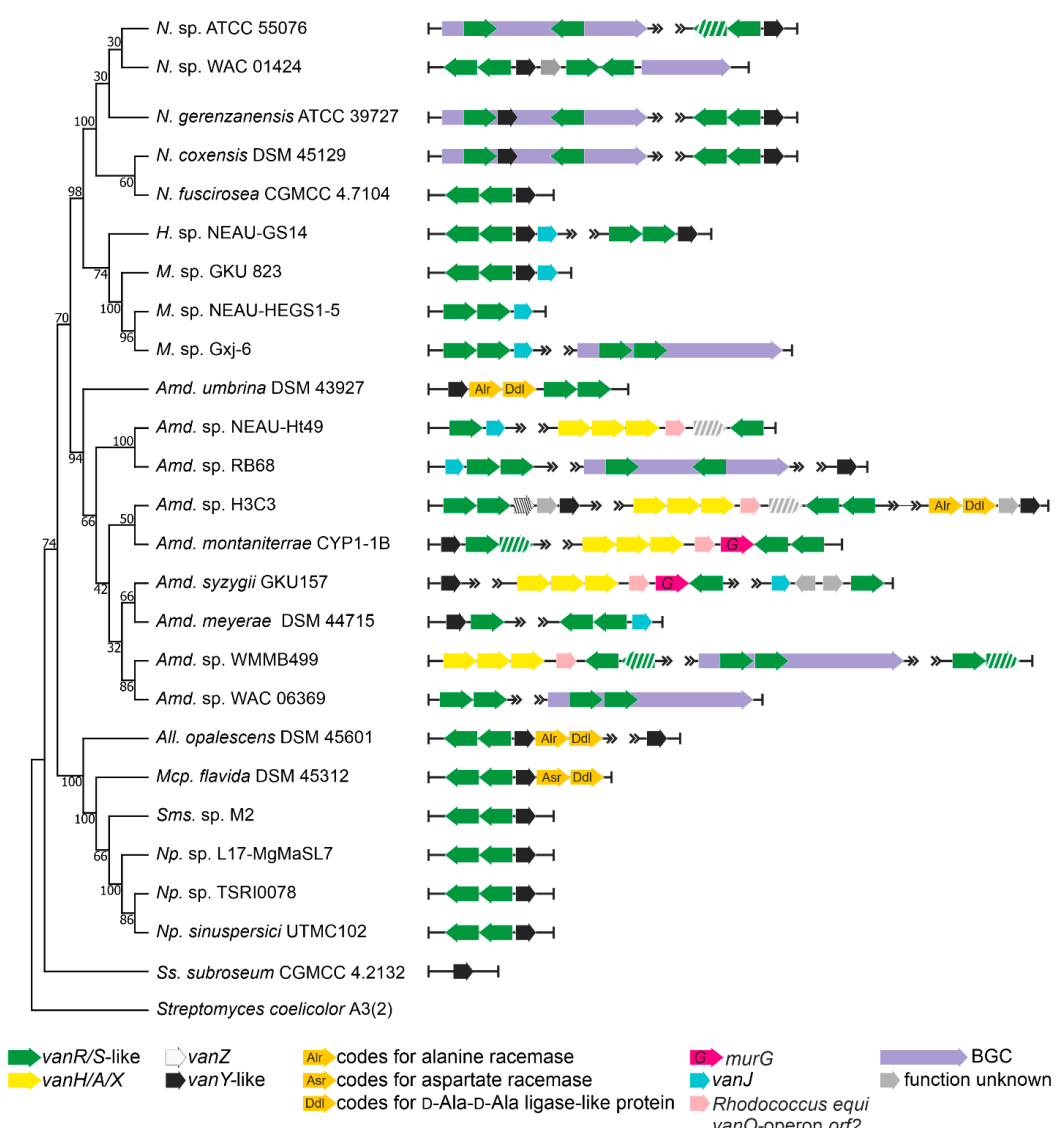
Organization of *vlgs* found within the set of 25 chosen genomes from *Streptosporangiales* spp. Maximum Likelihood phylogenetic tree of the 16S rRNA genes of the corresponding species (see Section 4 for details) served as a phylogenetic framework for the scheme. Names of *Streptosporangiales* genera were abbreviated according to ESM Table S1. The legend below the figure explains the color-coding of the scheme. Please refer to the text for the role of the single genes. Pseudogenes are shaded.

Going into more detail, we analyzed the genomes from the three known GPA producers, those from five strains carrying putative GPA-like BGCs, including *Nonomuraea* sp. WAC 01424 [18], together with 17 genomes of other *Streptosporangiales* spp. lacking any GPA BGCs (Figure 3). We found that the two Type IV GPA producers—*N. gerenzanensis* ATCC 39727 and *N. coxensis* DSM 45129—carried one BGC-situated *vanY*-like gene and an additional *vanY*-like gene, co-localized with a *vanRS*-like regulatory pair, distantly from the BGCs. The similarity of both distant- and cluster-encoded VanY-carboxypeptidases (amino acid sequence identity of 82.4% in *N. coxensis* and of 80.7% in *N. gerenzanensis*) was remarkable. The *vanRSY*-triad was found also in other *Nonomuraea* spp., lacking any GPA BGCs, such as in *Nonomuraea fuscirosea* CGMCC 4.7104 (Figure 3); it also was present on the chromosome of the kistamicin producer *Nonomuraea* sp. ATCC 55076 away from its BGCs, although this Type V GPA probably targets autolysins (such as corbomycin and complestatin [19]), thus not requiring *van* genes for self-resistance. Peculiarly, in the putative Type IV GPA producer *Nonomuraea* sp. WAC 01424 [18], GPA BGC seemed to be localized just downstream the *vanRSY* triad (which indeed was not cluster-situated in the other three GPA-producing *Nonomuraea* spp., Figure 3). WAC 01424 BGC carried another two-component regulatory pair, but without an additional copy of *vanY*.

Another two findings are worth mentioning. Similar to what was observed for *Pseudonocardiales,* we found that all *vanHAX* operons (present exclusively in *Actinomadura* spp. among *Streptosporangiales*) had a homologue of the *vanO* operon *orf2* (and sometimes a *murG-*like gene, too) downstream of *vanX* (Figure 3). Finally, the *vanRSY* triad was in rare cases co-localized with genes coding for alanine/aspartate racemase- and d-Ala-d-Ala-ligase (Ddl)-like proteins (e.g., *Allonocardiopsis opalecscens* DSM 45601 and *Murinocardiopsis flavida* DSM 45312, Figure 3), whose role would merit further investigation, indicating a possible alternative mechanism of cell-wall remodeling, such as the one reported in few enterococci based on the incorporation of d-Ala-d-Ser termini in the resistant peptidoglycan precursors [14].

#### 2.1.3. Order *Micromonosporales*

**Figure 4:**
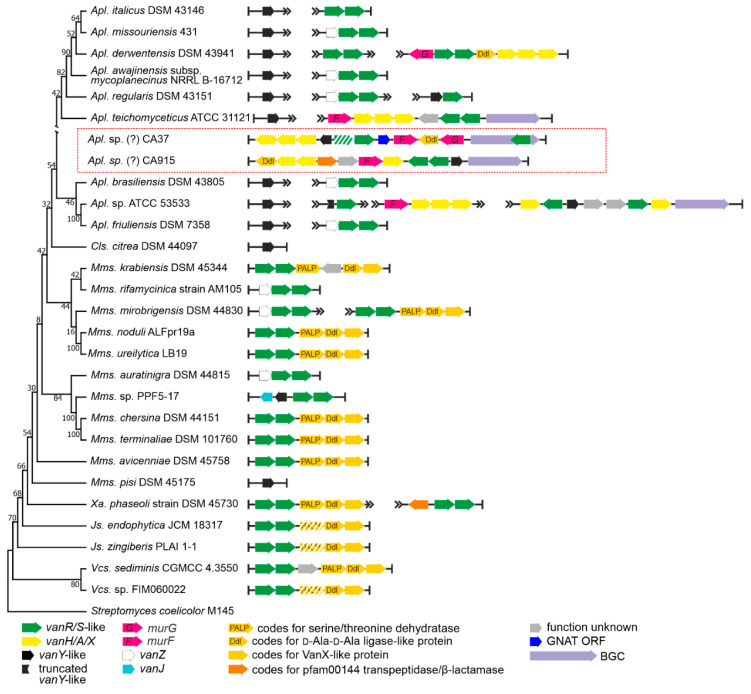
Organization of *vlgs* found within the set of 28 chosen genomes of *Micromonosporales* spp. Maximum Likelihood phylogenetic tree of the 16S rRNA genes of the corresponding species (see Section 4 for details) served as a phylogenetic framework for the scheme. Names of the *Micromonosporales* genera were abbreviated according to ESM Table S1. *vlgs* from the metagenomic CA915 and CA37 BGCs (highlighted with red frame) were arbitrarily introduced within the scheme since the retrieved sequences likely belong to some *Actinoplanes* spp. The legend below the figure explains the color-coding of the scheme. Please refer to the text for the role of the single genes. Pseudogenes are shaded.

The order *Micromonosporales* is rich in GPA producers coming from the genus *Actinoplanes* [42]. These are: (i) the clinically relevant lipo-GPA teicoplanin, coming from *Actinoplanes teichomyceticus* ATCC 31121 [43]; (ii) the sulfated GPA UK-68,597 [44], coming from *Actinoplanes* sp. ATCC 53533; and (iii) the hyperglycosylated GPA actaplanin from *Actinoplanes missouriensis* ATCC 23342 [45]. We screened 200 genome assemblies (Supplementary Excel File S1) and we found *vlgs* in 83 of them (Table 1, Supplementary Excel File S2). Complete sets of *vanHAXRS* genes were found only in GPA producers *Apl. teichomyceticus* (these *vlgs* were already studied experimentally [46]) and *Actinoplanes* sp. ATCC 53533, as well as in the GPA non-producer *Actinoplanes derwentensis* DSM 43941 (Figure 4).

We chose a total of 26 genomes (including only two genomes of GPA producers, since the genome of *Apl. missouriensis* ATCC 23342 is not yet available) for more detailed examination. *vanY-*like genes were found in *Actinoplanes* spp., although they were not co-localized with the *vanRS-*like regulatory pairs. Instead, the *vanRS-*like regulatory pairs were often found co-localized with *vanZ* genes (like in *Actinoplanes missouriensis* 431, Figure 4) or found without any other close *vlg* (such as in *Actinoplanes italicus* DSM 43146). However, one peculiarity specifically attracted our attention. In the course of our screenings, a particular gene arrangement was found to occur very often in the genomes of different *Micromonosporales* spp., especially in those belonging to the *Micromonospora* genus (Figure 4, Supplementary Excel File S2). This arrangement included a triad of genes coding for a PALP (pyridoxal-phosphate dependent)-like serine-threonine dehydratase, a Ddl-like protein and a VanX-like dipeptidase (further referred to as a *pdx* operon). Such a triad was accompanied with a VanRS-like regulatory pair. Overall, such an arrangement strikingly resembled typical *vanHAX-vanRS* operons, although the gene for lactate dehydrogenase was replaced by a serine-threonine dehydratase.

Finally, two metagenome-derived GPA-BGCs were described as related to the *Actinoplanes-*derived ones [18]. These were CA915 and CA37 [47]. Both of them were submitted to GenBank with rather long unannotated flanking regions. As in the case of CA878 (see above), we annotated the genes present on these flanks. The majority of the BGCs flanking genes seemed to be homologous to *Actinoplanes* spp. genes (ESM Figure S2). Thus, CA915 and CA37 most likely belong to some unknown *Actinoplanes* spp.

#### 2.1.4. Order *Streptomycetales*

**Figure 5:**
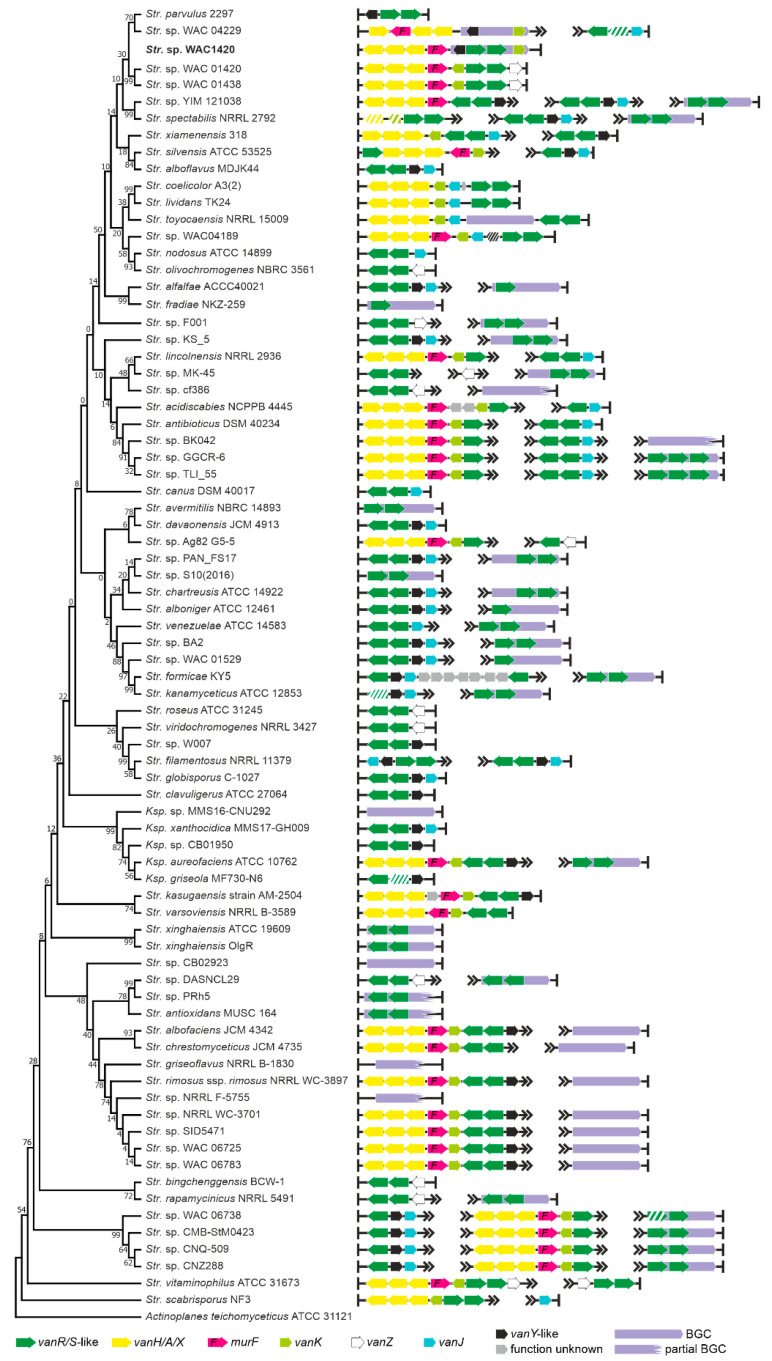
Organization of *vlgs* found within the set of 74 chosen genomes of *Streptomycetales* spp. Maximum Likelihood phylogenetic tree of the 16S rRNA genes of the corresponding species (see Section 4 for details) served as a phylogenetic framework for the scheme. Names of the *Streptomycetales* genera were abbreviated according to ESM Table S1. We were unable to detect the BGC for pekiskomycin within the published genome assembly of *Streptomyces* sp. WAC 01420, although the assembly contained *vlgs*; thus, *vlgs* in pekiksomycin BGC (as published in [49]) are given outside of the phylogenetic framework (highlighted in bold). Please refer to the text for the role of the single genes. Legend below the figure explains the color-coding of the scheme. Pseudogenes are shaded.

Although multiple genomes of *Streptomyces* spp. were sequenced (definitively more than in the other orders belonging to *Actinobacteria* phylum), only few Type I–IV GPA BGCs are known for this genus. These are A47934 BGCs from *Streptomyces toyocaensis* NRRL 15009 [48] and pekiskomycin BGCs from *Streptomyces* spp. WAC 04229 (WAC4229) and WAC1420 [49]. Our current analysis involved 1138 genome assemblies of *Streptomycetales* spp. (Supplementary Excel File S1), but no other novel BGC for Types I–IV GPA was found (Supplementary Excel File S2). On the contrary, BGCs for Type V GPAs and *feg-*like BGCs were found in 44 assemblies—all *Streptomyces* spp. except two *Kitasatospora* spp. Some of these BGCs were already reported [18], but we identified new ones (see Supplementary Excel File S2). *vlgs* were found exceptionally widespread in *Streptomycetales* spp.: more than one third of the analyzed genomes (418) contained *vlgs* (Table 1, Supplementary Excel File S2). Once again, we observed no correlation between the distribution of *vlgs* and of the GPA-like BGCs. More detailed analysis of 76 *Streptomycetales* spp. genomes (including 46 genomes carrying GPA- and *feg-*like BGCs) showed several different combinations, where certain strains carried a putative Type V GPA BGC and no *vlgs* (e.g., *Streptomyces fradiae* NKZ-259, Figure 5) along with strains carrying Type V GPA BGCs and a full set of *vlgs* (e.g., *Streptomyces* sp. NRRL WC-3897, Figure 5). Additionally, different combinations of *vlgs* were found in strains carrying no GPA-like BGCs. One peculiar trait of *Streptomycetales* spp. carrying the canonical *vanHAX-vanRS* operons was the presence of *vanK*, coding for an enzyme belonging to the Fem family, which adds the branch amino acid(s) to the stem pentapeptide of the peptidoglycan precursors carrying the d-Ala-d-Lac termini [50].

#### 2.1.5. Occurrence of *vlgs* in GPA Non-Producing Groups

**Figure 6:**
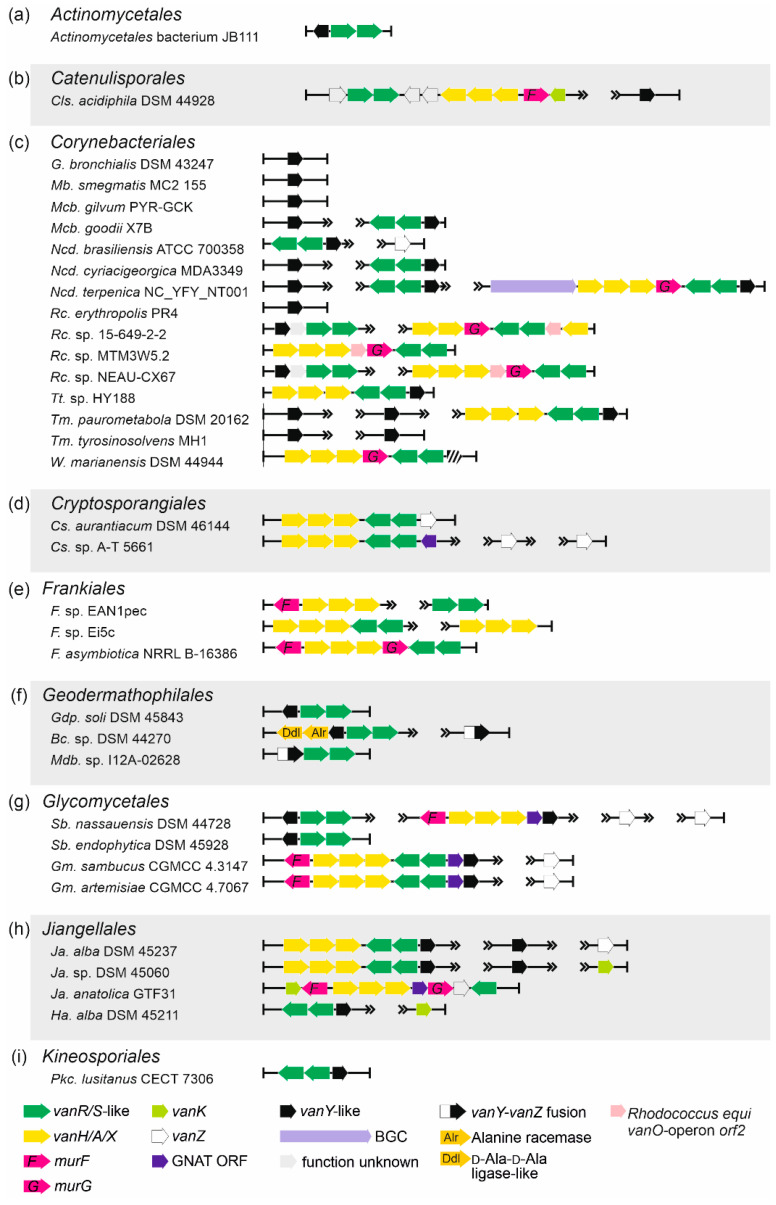
Arrangements of *vlgs* discovered in representative genomes from the orders (**a**) *Actinomycetales*, (**b**) *Catenulisporales*, (**c**) *Corynebacteriales*, (**d**) *Cryptosporangiales*, (**e**) *Frankiales*, (**f**) *Geodermathophilales*, (**g**) *Glycomycetales*, (**h**) *Jiangellales* and (**i**) *Kineosporiales*. Please refer to the main text for more details; genus names were abbreviated according to the ESM Table S1. The legend below the figure explains the color-coding of the scheme. Pseudogenes are shaded.

***Order Actinomycetales.*** Although being known for various opportunistic animal and human pathogens, *Actinomycetales* spp. did not carry multiple *vlgs*. The only taxon (out of the 223 genome assembles screened, Supplementary Excel Files S1 and S2) carrying *vanYRS* genes was *Actinomycetales* bacterium JB111 (Table 1, Figure 6a).

***Order Catenulisporales.*** Only three genomes of *Catenulisporales* spp. were available, and in one of them—*Catenulispora acidiphila* DSM 44928—a complete set of *vanHAXRSY* was found (Table 1, Figure 6b, Supplementary Excel Files S1 and S2).

***Order Coriobacteriales.*** Only two genomes out of the 217 available for *Coriobacteriales* spp. contained a complete set of *vanHAXRSY* (Table 1, Supplementary Excel Files S1 and S2; see the next paragraph for a more detailed description).

***Order Corynebacteriales****. vlgs* appeared to be quite common in *Corynebacteriales* spp. (707 genomes available for screening) (Table 1, Figure 6c, Supplementary Excel Files S1 and S2). Remarkably, a full set of *vlgs* (*vanHAXRS*) was discovered within the genome of *Williamsia marianensis* DSM 44944—a species isolated from Mariana trench (10.898 m below the sea level) in 1998 [51]. Other *vlgs* combinations included *vanY-*like genes paired with a *vanRS-*like regulatory pair; “orphan” *vanY-*like genes; and *vanHAXRSY* genes, often accompanied with *murG* genes and homologues of the *vanO* operon *orf2* (see typical examples on Figure 6c). Peculiarly, during this analysis, a putative unknown GPA BGC was found in the genome of *Nocardia terpenica* NC_YFY_NT001, which is a clinical isolate derived from human cerebrospinal fluid (see CP023778 genome assembly information).

***Order Cryptosporangiales*.** *vlgs* were found in two out of the three available genome assemblies of *Cryptosporangiales* spp. and were arranged as *vanHAXRS/HAXRSZ* (Table 1, Supplementary Excel Files S1 and S2); indeed, multiple copies of “orphan” *vanZ*-like genes were also present in the genome of *Cryptosporangium*. sp. A-T5661 (Figure 6d). In the genome of the latter, a gene coding for a GNAT was co-localized with *vanHAXRS-*genes (Figure 6d).

***Order Eggerthellales.*** Only in one genome out of the 106 available for *Eggerthellales*, a *vlg* was found (Table 1, Supplementary Excel Files S1 and S2; see the next paragraph).

***Order Frankiales.*** Approximately 15% of the analyzed *Frankiales* spp. genomes (46 in total, Supplementary Excel Files S1 and S2) possessed *vlgs*, arranged most often as *vanHAXRS* (and sometimes co-localized with *murF*- and *murG*-like genes) (Table 1, Figure 6e).

***Order Geodermathophilales.*** A large portion of the analyzed *Geodermathophilales* spp. genomes (60, Supplementary Excel Files S1 and S2) carried *vlgs*, namely, *vanY-*like genes co-localized with *vanRS-*like regulatory pairs (Table 1). Rarely, *vanYRS-*like genes were found together with genes coding for a Ddl and for an alanine-racemase (as it was observed in *Alr. opalescens* DSM 45601, Figure 6f). Another unique feature (discovered only in *Geodermathophilales* spp.) was the presence of genes coding for putative VanY–VanZ fusion proteins (e.g., in *Modestobacter* sp. I12A-02628, Figure 6f).

***Order Glycomycetales.****vlgs* were ubiquitously found in *Glycomycetales* spp. genomes (12 available in total), organized as *vanYRS*, *vanHAXY* or *vanHAXRSY* (Table 1, Supplementary Excel Files S1 and S2, Figure 6g). Latter arrangements were coupled with the genes coding for MurF and GNAT. Multiple copies of “orphan” *vanZ-*like genes were also found (Figure 6g).

***Order Jiangellales*.***vlgs* were found in more than 90% of the eleven analyzed *Jiangellales* spp. genomes (Table 1, Supplementary Excel Files S1 and S2). The most frequent arrangement was *vanHAXRSY,* although in *Jiangella anatolica* GTF31 *vanHAXS* genes were co-localized with *vanK* and with genes coding for MurF-, MurG- and GNAT-like proteins (Figure 6h).

***Order Kineosporiales.*** A single set of *vlgs* in this order—*vanRSY—*was found in *Pseudokineococcus lusitanus* CECT 7306 (Figure 6i) among the twelve genomes analyzed (Table 1, Supplementary Excel Files S1 and S2).

**Figure 7:**
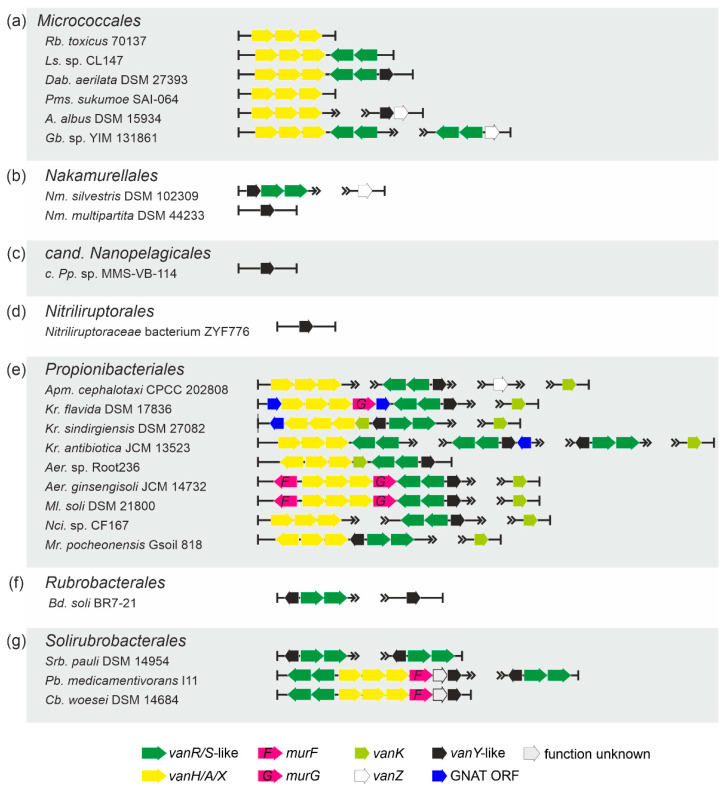
Arrangements of *vlgs* discovered in representative genomes from the orders (**a**) *Micrococcales*, (**b**) *Nakamurellales*, (**c**) *candidatus* Nanopelagicales, (**d**) *Nitriliruptorales*, (**e**) *Propionibacteriales*, (**f**) *Rubrobacterales* and (**g**) *Solirubrobacterales*. Please refer to the main text for more details; genus names were abbreviated according to the ESM Table S1. The legend below the figure explains the color-coding of the scheme.

***Order Micrococcales.****vlg*s were found in less than 1% of the analyzed *Micrococcales* spp. genomes (a total of 1741, Supplementary Excel Files S1 and S2) and were represented mainly as *vanHAX, vanHAXRS* or *vanHAXRSY* arrangements (Table 1, Figure 7a). *vanZ-*like genes, coupled with *vanRS-*like regulatory pairs, were also observed as well as “orphan” *vanY-*like genes co-localized with *vanZ* genes (Figure 7a).

***Order Nakamurellales.****vlgs* were found within all the 6 genome assemblies of *Nakamurella* spp., either as *vanYRS* or as “orphan” *vanY-*like and *vanZ* genes (Table 1, Figure 7b, Supplementary Excel Files S1 and S2).

***Order cand. Nanopelagicales and order Nitriliruptorales.*** In the few genome assemblies belonging to the species of both orders, only “orphan” *vanY-*like genes were rarely found (Table 1, Supplementary Excel Files S1 and S2, Figure 7c, d).

***Order Propionibacteriales.****vlgs* were found in the genome assemblies belonging to few genera of *Propionibacteriales* (Table 1, Supplementary Excel File S2), although it was possible to analyze 593 genomes (Supplementary Excel File S1). There, *vlgs* exhibited different arrangements, summarized in Figure 7e. Genes coding for MurF, MurG and GNAT proteins were often co-localized with *vlgs.*

***Orders Rubrobacterales and Solirubrobacterales.*** Only a small portion of species, belonging to both orders, carried *vlgs* within their genomes (Table 1, Supplementary Excel Files S1 and S2). *vlgs* mainly were arranged as either *vanYRS* or *vanRSHAXY* (sometimes co-localized with a *murF* gene, Figure 7f, g).

#### 2.1.6. Putatively Novel Transposable Elements Carrying *vlgs* in *Eggerthellales* and *Coriobacteriales* spp.

**Figure 8:**
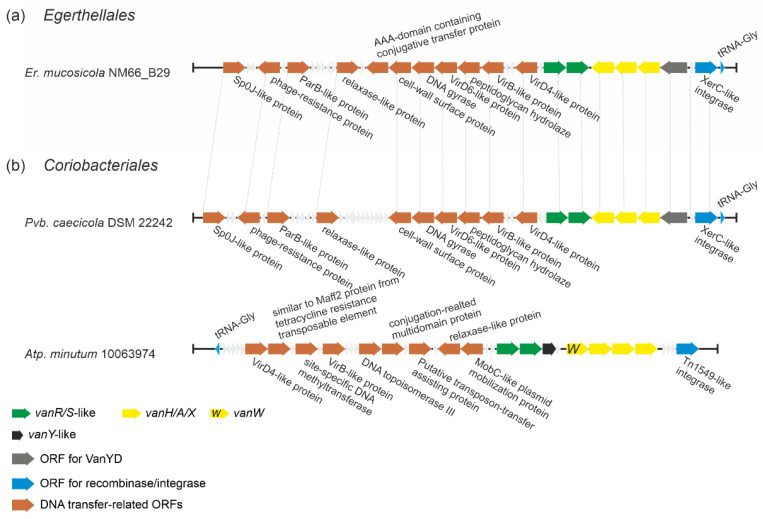
A scheme of putative MGEs found in (**a**) *Er. mucosicola* (*Eggerthellales*) and (**b**) *Coriobacteriales* spp. *Pvb. caecicola* and *Atp. minutum* (note that this MGE contains *vanW* gene – coding for a protein of unknown function). MGEs from *Er. mucosicola* and *Pvb. caecicola* seem to be almost identical (identical genes are joined with gray dashed lines), while MGE from *Atp. minutum* differs from both. The legend below the figure explains the color-coding of the scheme.

Analyzing the genomes of actinobacteria belonging to orders *Eggerthellales* and *Coriobacteriales,* we found *vlgs* in *Enterorhabdus mucosicola* NM66_B29, *Parvibacter caecicola* DSM 22242 and *Atopobium minutum* 10063974. When we examined the genetic neighborhood of these genes, it emerged that they might belong to the family of transposon-like mobile genetic elements (MGEs), involving multiple genes deputed to DNA transfer (Figure 8). Moreover, these *vlgs*-carrying putative MGEs were almost identical in *Er. mucosicola* NM66_B29 (order *Eggerthellales*) and *Pb. caecicola* DSM 22242 (order *Coriobacteriales*), while the MGE from *Atp. minutum* 10063974 (order *Coriobacteriales*) significantly differed from both (Figure 8). Transposons and other MGEs are believed to be one of the main sources of *van* genes dissemination throughout pathogens [52]. Thus, we decided to check whether MGEs from the three abovementioned species corresponded to those already known. For this, we compared putative integrases/recombinases from *Er. mucosicola* NM66_B29, *Pb. caecicola* DSM 22242 and *Atp. minutum* 10063974 with integrases found in known MGEs carrying *van* genes (ESM Table S2). It came out that the putative integrase from *Atp. minutum* 10063974 (EMZ42128) was identical to the integrase from *Enterococcus faecalis* transposon Tn1549 [53]. Further comparison of Tn1549 genes with the genes from *Atp. minutum* 10063974 MGE confirmed that they were identical. However, both integrases from *Er. mucosicola* NM66_B29 and *Pb. caecicola* DSM 22242 (EMZ42128 and MVX60893, respectively) were only slightly related to the transposases/integrases from Tn1549 and *Enterococcus faecium* insertion sequence IS1216V [54], sharing only an 18.6% and 14.6% amino acid sequence identity, respectively. Thus, MGE found in *Er. mucosicola* NM66_B29 and *Pb. caecicola* DSM 22242 might represent a novel MGE carrying *van* genes. Notably, this last putative MGE seemed to code for a VanYD protein [55], which is a d-Ala-d-Ala carboxypeptidase belonging to the penicillin-binding protein family, structurally unrelated to the VanY M15 peptidases (see below).

#### 2.1.7. Occurrence of *vlgs* in *Bacillales* spp. (*Firmicutes* phylum)

**Figure 9:**
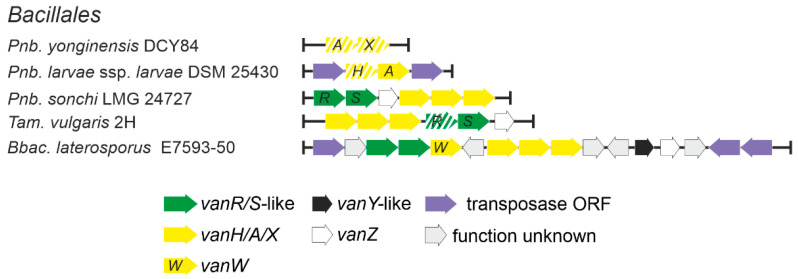
Arrangements of *vlgs* discovered in species belonging to *Bacillales* order. Please refer to the main text for more details; genus names were abbreviated according to ESM Table S1. The legend below the figure explains the color-coding of the scheme. Pseudogenes are shaded.

Low G-C soil Gram-positives and in particular bacilli were considered as a one of possible sources of *vlgs* for pathogens, as it was considered that the ancestral *vanHAX* cluster might have evolved into one such species (e.g., those belonging to the *Paenibacillus* genus) and was then disseminated to pathogens in a transposon-mediated fashion [28]. We decided to test such a hypothesis by screening 2379 full genome assemblies of *Bacillales* spp. (Supplementary Excel File S3), available in GenBank, searching for *vlgs.* The results indicated that *vlgs* were quite rare in *Bacillales* spp. genomes: *vanHAXRS* were found in the genomes assemblies of *Brevibacillus laterosporus* E7593-50, *Thermoactinomyces vulgaris* and *Paenibacillus sonchi* LMG 24727 (Figure 9, Supplementary Excel File S3). *vanAX-*pseudogenes were also found in *Paenibacillus yonginensis* DCY84, while *vanHA-*genes (degraded to different extents) were found in 7 strains of *Paenibacillus larvae* (Figure 9, Supplementary Excel File S3). Only one *vanY*-like gene was found in *Bbac. laterosporus* E7593-50. Most of the *vlgs* were found adjacent to transposase-related genes, except the cases of *Pnb. yonginensis* DCY84, *Pnb. sonchi* LMG 24727 and *Tam. vulgaris* 2H; indeed, the latter strain is known to be naturally competent for exogenous DNA [56]. Overall, such findings indicated that the occurrence of *vlgs* in bacilli is not comparable to their distribution in most of the orders belonging to actinobacteria, making it highly unlikely for *van* genes to arise independently in bacilli.

### 2.2. Phylogeny of VanY-like Carboxypeptidases

Surprisingly, *vanY-*like genes were the most common *vlgs* found in actinobacteria. We decided to reconstruct the phylogeny of the VanY-like proteins to comprehend such a variety and its relation to similar proteins described in low G-C Gram-positives, including pathogens such as GPA-resistant enterococci. For this purpose, we selected 251 proteins (see Section 4) coming either from actinobacteria or from low G-C Gram-positives, which, according to the MEROPS peptidase database [57,58], belonged to the M15B subfamily of the M15 family of metallopeptidases (mostly carboxypeptidases and dipeptidases). Other subfamilies are M15A, composed of a specific group of the so-called *Streptomyces-*type zinc-d-Ala-d-Ala carboxypeptidases [59], and M15D, to which VanX d-Ala-d-Ala dipeptidases belong (see the following section) [60,61]. To check that our selected proteins were VanY-like and no other carboxypeptidases, we controlled their sequence by CD-Search [58] and excluded those sharing the putative peptidoglycan-binding domain on the N-terminal region, which is typical of M15A proteins.

**Figure 10:**
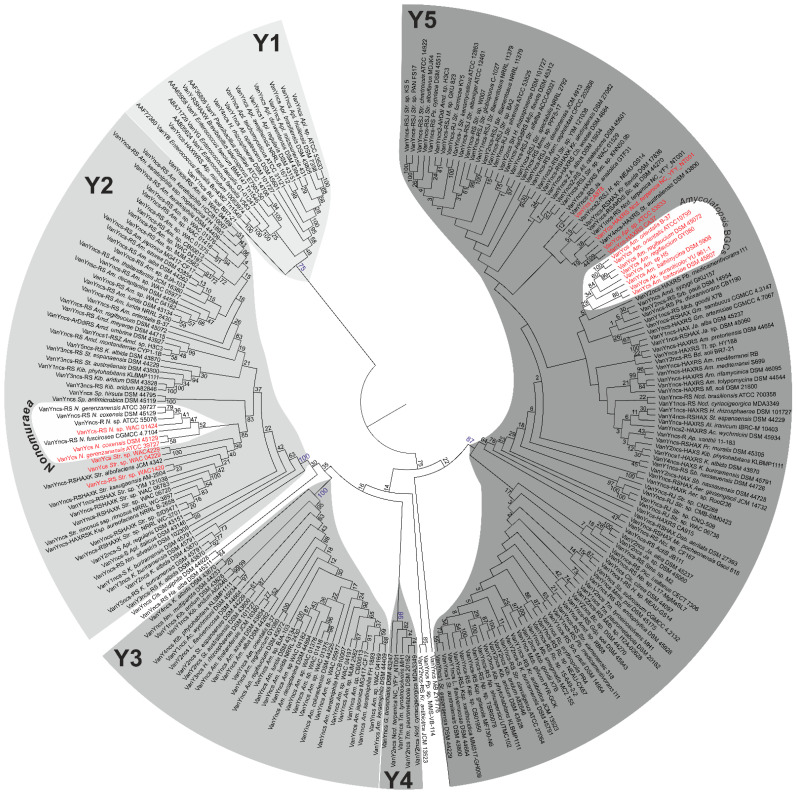
Maximum Likelihood phylogenetic tree of 251 VanY-like M15B carboxypeptidases. To show the topology of the tree better, branch lengths were ignored; the same tree drawn to scale is given in ESM Figures S3–S7. Five well supported clusters—Y1 to Y5—were distinguished on the tree. “cs/ncs” abbreviations in the label at the tip of each branch mean “cluster-situated/non-cluster-situated”. BGC-encoded proteins are given in red. Importantly, the most-studied VanY_n_ (Dbv7) from the A40926-producing *N. gerenzanensis* ATCC 39727 [62] belongs to Y2, while VanY_Ab_ (coming from the balhimycin producer *Am. balhimycina* DSM 5908 [63]) belongs to the Y5 cluster.

Reconstruction of the Maximum Likelihood phylogeny of VanY-like proteins from our dataset yielded a tree, where 5 distinct clusters were differentiated (Figure 10, ESM Figure S3). Cluster Y1 (Figure 10, ESM Figures S3 and S4) was the outgroup of the tree and contained VanY-like proteins originating from enterococci and other low G-C Gram-positives, consistently with previous reports [37]. Differently from what described a decade before [38], this cluster included also VanY-like proteins coming from different *Actinoplanes* spp., such as *Bd. soli* BR7-21, *H. rhizosphaerae* DSM 101727 and *All. opalescens* DSM 45601 (Figure 10, ESM Figure S4). This discrepancy is due to the increasing number of genomes that have become accessible in the meantime. Notably, all the genes corresponding to Y1 proteins in actinobacteria were ‘orphan’ (i.e., not co-localized with any other *vlgs*; see Figure 3, Figure 4 and Figure 7, and Supplementary Excel File S2).

In contrast, the VanY-like proteins from the large Y2 cluster were almost exclusively encoded by genes adjacent to the *vanRS-*like pairs and/or other *vlgs* (Figure 10, ESM Figure S5, Supplementary Excel File S2). They were found in the genomes of multiple orders of actinobacteria (Figure 10, ESM Figure S5, Supplementary Excel File S2). Importantly, Y2 included the VanY-like proteins from *Nonomuraea* GPA BGCs (including the most-studied VanY_n_ from *N. gerenzanensis* ATCC 39727 [62]) and pekiskomycin BGCs (*Streptomyces* spp. WAC1420, WAC4229, WAC 04229 [49]).

The next clusters—Y3 and Y4 (Figure 10, ESM Figure S6)—were composed of VanY-like proteins coming from *Pseunocardiales* and *Corynebacteriales,* respectively, with the corresponding genes again being mostly ‘orphan’.

Finally, Y5 was the last cluster defined within the tree of the VanY-like proteins. It was the biggest and most separated from the others, although the internal branching pattern was not completely clear, often lacking a trustable bootstrap support (Figure 10, ESM Figure S6). Nevertheless, genes corresponding to Y5 proteins were found either co-localized with different other *vlgs* or ‘orphan’. The VanY-like proteins coded by *Pseudonocardiales* spp. GPA BGCs, including the most-studied VanY_Ab_ from the balhimycin producer *Am. balhimycina* [63], formed a distinct subclade within Y5. The VanY-like proteins encoded within CA878, CA37, CA915, *auk* and *Ncd. terpenica* NC_YFY_NT001 BGCs were also found in Y5.

### 2.3. Phylogeny of VanHAX

***VanH lactate dehydrogenases.*** For the phylogenetic reconstruction of VanH, we used a dataset of 156 VanH proteins from actinobacteria and low G-C Gram-positives, with SCO2118 lactate dehydrogenase serving as an outgroup (see Section 4). It appeared that VanH proteins were quite conserved, and a branching pattern with completely reliable bootstrap support was difficult to obtain (ESM Figure S8). Nevertheless, few features could be presumed with certainty. First, VanH proteins coded within the putative MGEs of *Er. mucosicola* NM66_B29 and *Pvb. caecicola* DSM 22242 reliably out-grouped all other proteins (ESM Figure S8). Then, a well-defined cluster (named VH1, ESM Figure S8) was composed of VanH proteins from pathogens and from low G-C soil Gram-positives. VH1 was not that close to the base of the tree, with multiple actinobacterial VanH proteins out-grouping it (ESM Figure S8). Another well-supported cluster—VH2 (ESM Figure S8)—was composed of VanH proteins coming from different *Streptomyces* spp., including those from A47934 BGC (*Str. toyocaensis* NRRL 15009) and pekiskomycin BGC (*Streptomyces* sp. WAC 04229). A third big cluster (VH3) was formed by VanH proteins coming from different GPA non-producing *Streptomyces* spp. Finally, VanH proteins coded within *tei, auk,* CA37 and CA915 also grouped together on the tree (ESM Figure S8).

***VanA d-Ala-d-Lac-ligases.*** Overall, from previous works, it seems plausible that VanA d-Ala-d-Lac-ligases are a specialized evolutionary branch of d-Ala-d-Ala ligases (Ddl) involved in the “primary metabolism” of the cell wall in actinobacteria [64]. As reported above, Ddl-like proteins were also found encoded in metagenomic-sourced CA37, CA915 and in the *auk* BGCs; additionally, in the *Micromonosporales* we found a peculiar putative *pdx* operon (see Section 2.1.3) composed of genes for a PALP threonine dehydratase, a Ddl-like ligase and a VanX-like dipeptidase, adjacent to a *vanRS-*like regulatory pair. Thus, here we decided to test the phylogeny of the VanA ligases together with the above-mentioned Ddl-like proteins on a background of ‘house-keeping’ Ddl-ligases from the main actinobacterial orders. The protein set (see Section 4) used for this phylogenetic reconstruction contained 153 VanA ligases from actinobacteria and low G-C Gram-positives, 12 Ddl-like ligases from *Micromonosporales* spp., the 3 Ddl-like ligases from CA37, CA915 and *auk* BGCs, as well as 81 ‘house-keeping’ actinobacterial Ddl-ligases.

In the resulting phylogenetic tree (ESM Figure S9), the ‘house-keeping’ Ddl-ligases formed a number of distinct clades that corresponded to the orders of origin (ESM Figure S9). There, Ddl-like ligases coded in CA915, CA37 and *auk* BGCs grouped together with “house-keeping” Ddl-ligases from order *Eggerthellales*, while Ddl-like ligases from *Micromonosporales* (coded in putative *pdx-*operons) formed a distinct clade among the other clades of ‘house-keeping’ Ddl-ligases (ESM Figures S9 and S10). Most strikingly, clades for VanA ligases from low G-C Gram-positives (ESM Figure S11) and actinobacteria (ESM Figure S12) (both out-grouped by VanA ligases coded within the putative MGEs of *Er. mucosicola* NM66_B29 and *Pvb. caecicola* DSM 22242) clustered together with ‘house-keeping’ Ddl-ligases from the order *Coriobacteriales* (ESM Figure S9).

Composition of the main actinobacterial VanA-clade (ESM Figures S9 and S12) required further comments. The resolution of this clade was high enough to distinguish four well-supported clusters—VA1–4. VA1 was formed by the VanA ligases from GPA non-producing streptomycetes together with the one coded in A47934 GPA BGC from *Str. toyocaensis* NRRL 15009. Other VanA ligases from GPA non-producing streptomycetes were composed of VA2, while VA3 contained proteins from different groups of actinobacteria. Finally, VA4 was formed by VanA ligases coded within GPA BGCs of *Amycolatopsis* spp. VanA ligases from CA915, CA37 and *tei* BGCs grouped together on the tree. Notably, VanA ligases from pekiskomycin BGCs (from *Str.* sp. WAC 04229 and *Str.* sp. WAC1420) grouped together with VanA from *Str. varsoviensis* NRRL B-3589, which does not carry any GPA BGC.

***VanX M15D dipeptidases.*** According to the MEROPS database, VanX dipeptidases belong to the same M15 family as VanY carboxypeptidases, but group in the M15D subfamily. The dataset used for the phylogenetic reconstruction contained 155 VanX dipeptidases coded within different *vanHAX* operons from actinobacteria and low G-C Gram-positives and 12 VanX-like dipeptidases coded in the putative *pdx* operon found in *Micromonosporales.* The obtained phylogenetic tree revealed that VanX-like dipeptidases coded in the *pdx* operon and VanX proteins from low G-C Gram-positives formed well-supported clusters (VX1 and VX2, respectively, ESM Figure S13). All other actinobacterial VanX-like dipeptidases were out-grouped by the VanX-like dipeptidases coded within the putative MGEs of *Er. mucosicola* NM66_B29 and *Pvb. caecicola* DSM 22242 (ESM Figure S13). Unfortunately, internal topology of the latter clade had non-optimal bootstrap support, since the VanX-proteins appeared well conserved (ESM Figure S14). However, it was possible to distinguish two clusters—VX3 and VX4. Interestingly, the composition of VX3 and VX4 corresponded to the clusters VA4 and VA3, respectively, of th eVanA-like ligases tree (ESM Figure S12). Finally, the VanX dipeptidases coded in pekiskomycin BGCs (from *Str.* sp. WAC 04229 and *Str.* sp. WAC1420) again grouped together with VanX from *Str. varsoviensis* NRRL B-3589, which is not a GPA producer.

The *van* genes from pathogenic enterococci often code a peculiar group of bifunctional d,d-dipeptidases/d,d-carboxypeptidases, known as VanXY [65]. In 2014, a comparative structural study of VanXY, VanX and VanY [57] assumed VanXY peptidases to be a specialized evolutionary branch of VanY carboxypeptidases in pathogens. This assumption was supported with a phylogenetic reconstruction of the M15 family dipeptidases [57]. However, rather few protein sequences were available at that time. Thus, we decided to check the phylogeny of the VanXY peptidases in relation to our VanX and VanY datasets simultaneously. We used a dataset of 425 proteins, including 7 VanXY-peptidases. The topology of the resulting tree correlated with the topologies of the trees received for the VanX and VanY datasets, separately. At the same time, the VanXY peptidases appeared to root deeply within the VanY clade (ESM Figure S15) from low G-C Gram-positives (corresponding to the Y1 cluster of VanY carboxypeptidases, Figure 10). Therefore, our large-scale reconstruction was in line with the previously made assumption about the origin of VanXY [57].

### 2.4. Phylogeny of VanRS-like Two-Component Regulatory Pairs

**Figure 11:**
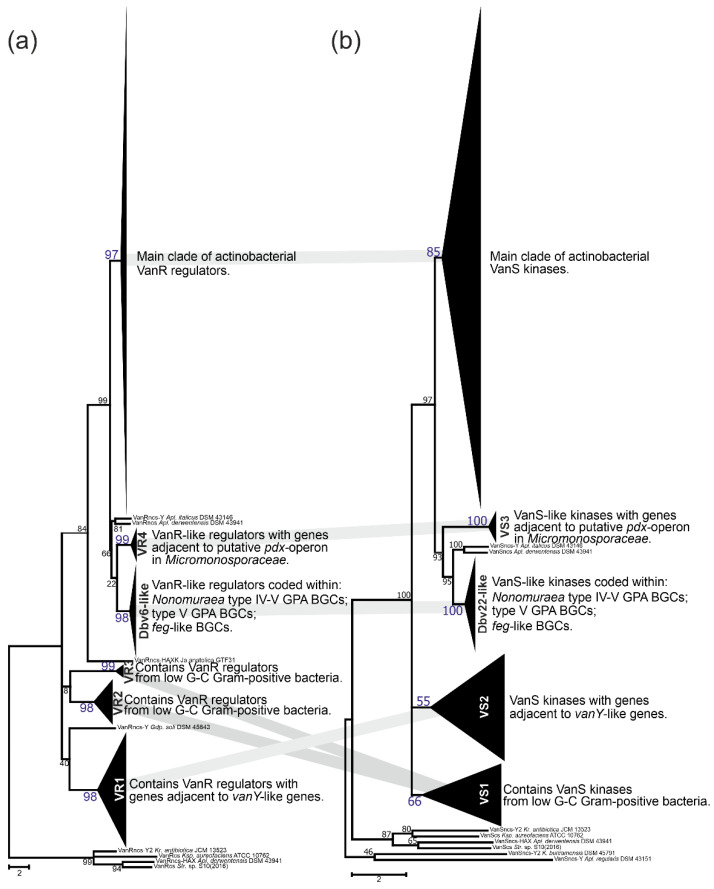
Phylogenetic trees of actinobacterial VanR-like response regulators (**a**) and VanS-sensor histidine kinases (**b**). Defined clusters on each tree were collapsed; for the expanded versions, please refer to ESM Figures S16–31. Coherent clusters are joined with thick grey lines. Please see main text for more details. Scale bar represents the number of substitutions per site.

In the course of our screen for *vlgs*, many of them were found co-localized with *vanRS-*like regulatory pairs. *vanRS-*like regulatory pairs were also found adjacent to (i) a putative *pdx* operon in *Micromonosporales*; (ii) putative operons formed with genes coding for alanine/aspartate racemase, Ddl and VanY-like carboxypeptidase (such as in *All. opalescens* DSM 45601 and *Mcp. flavida* DSM 45312, respectively, Figure 3); or (iii) genes for β-lactamases (such as in *Xa. phaseoli* DSM 45730, Figure 4). Additionally, multiple BGCs for Type V GPAs and *feg-*like BGCs from *Streptomyces, Microbispora* and *Actinomadura* spp., as well as for Type IV GPAs from *Nonomuraea* spp., were found to carry *vanRS-*like regulatory pairs. To clarify if, how and to what extent all these VanR-like response regulators and VanS-like sensor histidine kinases are related to each other and to the VanRS proteins from low G-C Gram-positives, we reconstructed separate phylogenies of the two datasets: one for the VanR- and another for the VanS-like proteins. The first dataset contained 295 proteins, while the second was composed of 313 proteins. Such a discrepancy in numbers derives from the fact that the *vanRS-*like pairs often lacked one of the genes or had it impaired (as pseudogene). Overall, the final topology of both trees was quite coherent, implying the co-evolution of the VanR- and VanS-like proteins coded within one gene pair (Figure 11); this allowed us to define a set of well-supported clusters (named VS in the VanS and VR in VanR trees). Both trees showed a set of clusters formed as basal clades as well as defined crown groups. Basal clades formed VR1–4 clusters plus a Dbv6-like cluster in the VanR phylogenetic tree, and VS1–3 clusters plus a Dbv22-like cluster in the VanS phylogenetic tree. These data were consistent with what was previously reported on Dbv6/Dbv22, which is a cluster-situated two-component regulatory pair in A40926 BGC from *N. gerenzanensis,* not grouping with the “classic” VanRS-like proteins from other GPA BGCs [66].

Proteins from coherent clusters VR1 (VanR-tree, Figure 11a, ESM Figure S16) and VS2 (VanS-tree, Figure 11b, ESM Figure S17) had genes co-localized with *vanY-*like genes, coding for Y2-cluster VanY-like proteins (Figure 10); the overall topology of Y2 was found similar to both VR1 and VS2 (ESM Figures S4, S16 and S17). Then, the VR2+3 and VS1 clusters were also coherent and contained VanS- and VanR-like proteins found in low G-C Gram-positives, as well as the ones coded within the MGEs from *Coriobacteriales* and *Eggerthellales* spp. (Figure 11, ESM Figures S18 and S19). Next pair of coherent clusters contained VanS- and VanR-like proteins coded within *feg-*like BGCs, Type V GPA BGCs and Type IV GPA BGCs from *Nonomuraea* spp. (Figure 11). Basically, both clades were formed with orthologues of either Dbv6 or Dbv22 from *dbv* BGCs, thus they received the corresponding naming (ESM Figures S20 and S21). Finally, the last pair of coherent basal clusters were VR4 and VS3 (Figure 11), formed by VanR- and VanS-like proteins coded adjacent to the putative *pdx* operon from *Micromonosporales* (ESM Figures S22 and S23).

Similar to what was previously observed for VanX, VanA and VanH phylogenies, the resolution of VanR and VanS trees crown groups was not perfect (ESM Figures S24 and S25). Nevertheless, we defined three additional clusters in the VanR-phylogenetic tree—VR5–7 (ESM Figures S24 and S26). VanR regulators from clusters VR5–6 were coded adjacent to *vanY-*like genes in different actinomycetes, while VR7 was composed of VanR-regulators coded adjacent to *vanHAXYK* in *Streptomyces* spp. VR7 contained VanR coded within pekiskomycin BGC from *Str.* sp. WAC1420. The resolution of the VanS tree crown group was better, allowing to distinguish five additional clusters—VS4–8 (ESM Figure S25). Here, the VS4 and VS5 clusters were formed by VanS-kinases coming from different actinobacterial orders and coded adjacent to the *vanY-*like genes and *vanZ-*like genes, respectively (ESM Figures S27 and S28). Then, proteins forming the VS6 cluster were coded adjacent to the *vanHAX* genes in different actinobacteria (ESM Figure S29), while VS7 was formed by VanS kinases coded adjacent to *vanHAXYK* genes from *Streptomyces* spp., including the one from pekiskomycin BGC (*Str.* sp. WAC1420, ESM Figure S30). Finally, proteins from VS8 were coded adjacent to *vanHAXK* genes in streptomycetes; VS8 also included VanS from model *Str. coelicolor* (ESM Figure S31).

Some other notable features in the VanS and VanR crown groups phylogenies require comments. First, both reconstructions placed VanS and VanR proteins encoded within *tei* and A47934 BGCs together (ESM Figures S25 and S26). Second, both trees showed evidence of a possible evolution of the VanRS-like regulatory pair, expanding its regulon control from *van* genes to some other genes: although the VanRS pair from *All. opalescens* DSM 45601 and *Mcp. flavida* DSM 45312 were related to the VanRS proteins coded adjacent to the *vanHAX* genes, the corresponding *vanRS-*like gene pairs actually were co-localized with genes for alanine or aspartate racemases, together with *ddl* and *vanY-*like genes.

## 3. Discussion

In the current work we aimed to address certain unclear issues about *van* genes, such as their distribution and phylogeny. Although we are aware that our results might risk generating more questions than answers, we tried in the following section to summarize what are, in our opinion, the most relevant findings.

***Actinobacteria are the most likely primary sources of vlgs.*** First of all, the results of our screens, covering more than 7000 actinobacterial genomes and 2000 *Bacillales* genomes, revealed that *vlgs* are abundant within the *Actinobacteria* phylum (with an incidence of ca. 13%), while vanishingly rare in bacilli and related species belonging to the *Firmicutes* phylum. This disproves the idea of *van-*like genes and operons emerging independently in soil-dwelling actinobacteria and bacilli [28]. Abundance and context variability of actinobacterial *vlgs* point to the *Actinobacteria* phylum as to the original source of *van* genes. At the same time, the assumption that ubiquitous low G-C soil Gram-positives served as a bridge for *vlgs* to arrive in pathogens [28] seems likely. Such a transfer was probably achieved via MGEs, which often were proved to carry *vlgs* in low G-C soil Gram-positives and pathogens [52]. In fact, we found the classical Tn1549 transposon in the actinobacterium *Atp. minutum* 10063974, even though until now Tn1549-like transposons were described only in enterococci [53]. It is hard to say whether *Atp. minutum* 10063974 might represent the original actinobacterial source of Tn1549, or if this is an example of reverse HGT event: G-C content (estimated from *vanRSYWHAX* genes) of *Atp. minutum* 10063974 Tn1549 is the same as in *Enterococcus faecalis* BM4382 Tn1549—47%. However, this is comparable to the overall genome G-C content of *Atp. minutum* 10063974, which is ca. 48%. At the same time, two novel, putative MGEs (very similar to each other) were also found in the genomes of the actinobacteria *Er. mucosicola* NM66_B29 and *Pvb. caecicola* DSM 22242. These MGEs coded unusual transposases, carrying *vlgs* coding proteins that in our phylogenetic reconstructions did not cluster with Van proteins derived from low G-C Gram-positives. Once again, it is not clear if these MGEs might represent the ‘original’ actinobacterial *vlg*-carrying elements. The G-C content of MGEs from *Er. mucosicola* NM66_B29 and *Pvb. caecicola* DSM 22242 (as estimated from the G-C content of their *vanRSHAXY-D*-genes) is ca. 55%. This is higher than the usual G-C content of MGEs from low G-C Gram-positives, but lower than the overall genome G-C content of *Er. mucosicola* NM66_B29 and *Pvb. caecicola* DSM 22242 (64.6% and 62.4%, respectively). Thus, the study of *vlgs* in the soil mobilome requires more detailed and focused research, which could in future contribute to a better understanding of how *vlgs* were disseminated from actinobacteria.

***vlgs are distributed in actinobacteria without any evident strict correlation to GPA BGCs distribution, although a complex co-evolution with BGCs likely occurred.*** Our comparative genomic analysis of the different orders belonging to the *Actinobacteria* phylum showed that *vlgs* are not necessarily co-localized with Type I–IV GPA BGCs; moreover, in the majority of the cases, *vlgs* were found in GPA non-producers as well as in Type V GPA producers. Consequently, it is reasonable to assume that non-cluster-situated *vlgs* existed independently from GPA BGCs and might actually be considered a preadaptation feature, which then facilitated the spread of GPA BGCs within the *Actinobacteria* phylum. Next, GPA cluster-situated *vlgs* are not monophyletic, meaning that different GPA BGCs likely acquired these genes from different sources of the vast actinobacterial pool. All our phylogenetic reconstructions in fact showed that cluster-situated *vlgs* emerged randomly on the trees, surrounded by non-cluster-situated *vlgs*. One of the most prominent lines of evidence came from the VanY phylogeny, where *Nonomuraea* spp. and *Amycolatopsis* spp. GPA cluster-encoded VanY carboxypeptidases belonged to distant clusters (Y2 and Y5, Figure 9), separated by multiple other non-cluster-encoded VanY proteins.

**Figure 12:**
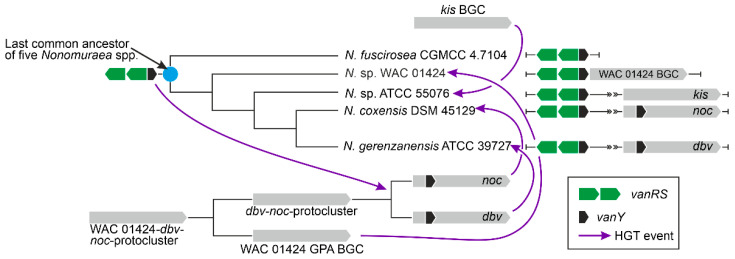
Scheme for the discussed scenario for *vanY*-like genes and GPA BGCs co-evolution in *Nonomuraea* spp. Please refer to the main text for more details.

Moreover, *vlgs* and GPA BGCs seem to have a shared, complex co-evolution pattern. We were able to reconstruct one of the most obvious ones, observed for the self-resistance phenotype in the A40926 producer *N. gerenzanensis*, which relied on the expression of the cluster-situated *vanY*-like gene—*dbv7.* In the corresponding *dbv7* knockout mutant, GPA resistance was significantly reduced, but not completely abolished [62]. Screening the full genome of *N. gerenzanensis* [67], the reason became evident: an additional *vanY* allele was found far away the *dbv* BGC. Interestingly, this allele was co-localized with a *vanRS-*like regulatory pair, whereas *dbv7* was not. The same situation was observed for *N. coxensis* DSM 45129—a recently described producer of A40926-like Type IV GPA [40,68]: a cluster-situated *dbv7* homologue (almost identical) and a non-cluster-situated *vanRSY-*triad were identified in its genome. The two other screened genomes of *Nonomuraea* spp.—the GPA non-producing *N. fuscirosea* CGMCC 4.7104 and the kistamicin producer *Nonomuraea* sp. ATCC 55076—contained close homologues of the *vanRSY-*triad. In addition, the genome of the other putative GPA producer *Nonomuraea* sp. WAC 01424 presented the GPA BGC just upstream the *vanRSY*-triad, while no *vanY-*allele was found in the BGC itself. Finally, in our reconstructed phylogeny of VanY-like carboxypeptidases, the *dbv* and *noc* cluster-encoded VanY-proteins out-grouped (Y1 cluster) all other ones (Figure 10). We believe that such a picture is as a result of a series of HGT events. In our hypothesis, summarized in Figure 12, first, *noc* and *dbv* protoclusters recruited the *vanY* gene from the *vanRSY* triad present in the common ancestor of *Nonomuraea* spp., whereas the WAC 01424 and kistamycin ancestral BGCs did not acquire it. Then, *noc* and *dbv,* through another HGT event, were introduced in the *Nonomuraea* ancestors already carrying the *vanRSY* triad. Another HGT event delivered WAC 01424 BGC to *Nonomuraea* sp. WAC 01424 and kistamycin BGC to *Nonomuraea* sp. ATCC 55076, explaining why the *vanRSY* triad is the only resistance determinant in these strains. Finally, no such HGT event occurred in *N. fuscirosea* CGMCC 4.7104, leaving this strain without any GPA BGCs but still carrying the *vanRSY* triad.

***Is the GPA resistance the original function of vlgs?*** As reported above, actinobacteria are an extremely abundant source of *vlgs.* It is quite difficult to consider that such a variety of *vlgs* in GPA non-producing actinobacteria is needed to protect them versus the GPA producers, which definitively seemed quite rare. Alternatively, *vlgs* might have natural functions in cell-wall remodeling in response to environmental and/or developmental triggers. For instance, VanY-like M15B carboxypeptidases are extremely abundant in actinobacteria, forming diverged evolutionary lineages; it is tempting to suspect their role in, for instance, cell-wall remodeling during the life cycle (a function indeed shown for some other d-Ala-d-Ala carboxypeptidase [69]). Our finding of the so-called *pdx* operon, common for *Micromonosporales,* contributed to such an idea. This putative operon is composed of a PALP-like serine-threonine dehydratase, a Ddl ligase and a VanX-like dipeptidase genes, co-localized with a *vanRS-*like regulatory pair. This organization suggests an alternative mechanism of peptidoglycan precursor remodeling, which reminds the introduction of d-Ala-d-Ser termini in the cell wall of some enterococci, showing a low level of GPA resistance [14]. It is unknown whether the *pdx* operon is functional and if its expression leads to some cell wall remodeling, but such a case indeed merits further experimental evaluation.

To conclude, we must say that the current work only scratched the surface of actinobacterial *vlgs*, leaving many issues without a complete final evaluation and understanding. However, we hope that this should provoke other in silico, in vitro and in vivo studies, which will shed light on such an important question such as GPA resistance. Our findings portray actinobacteria as a Pandora’s box, hosting myriads of putative GPA resistance genes that might be transferred sooner or later to pathogens, significantly contributing to AMR. A thorough understanding of GPA resistance in actinobacteria may prove useful in the future surveillance of emerging mechanisms of resistance to clinically used GPAs. Although further experiments are necessary to show that the discovered in silico putative *vlgs* have a real function in vivo conferring GPA resistance, their study may reveal new insights into their biological functions in actinobacteria, augmenting our comprehension of this remarkable phylum.

## 4. Methods

### 4.1. Routine Analysis of Nucleic and Amino Acid Sequences

All routine analytic work with nucleic acid and amino acid sequences was performed using Geneious 4.8.5 [70], Mega X [71]; routine amino acid sequence alignments were performed with Clustal Omega (EMBL-EBI) [72].

### 4.2. vlgs Search Pipeline

To perform the search for *van-*like genes (named *vlgs*), all genome assemblies of *Actinobacteria*—available at the time of work preparation (April 2020) as either full sequences or uncompleted ones—were retrieved from the GenBank database (in few exceptions, genome assemblies were retrieved from RefSeq database), for a total of 7108 assemblies from species belonging to 26 established and 2 tentative orders within the *Actinobacteria* phylum, represented by 653,111 corresponding nucleic acid sequences. A full list of the genome assemblies and corresponding nucleic acid sequences is given in Supplementary Excel Table S1. The MultiGeneBlast tool [73] was utilized to screen the chosen set of genome assemblies for *vlgs*. To do that, the chosen set of genome assemblies belonging to each order was downloaded from GenBank (or RefSeq) in a genomic GenBank format (*.gbff). These files were then used to create offline MultiGeneBlast custom databases for each order belonging to the *Actinobacteria* phylum. MultiGeneBlast was run in a “homology” mode with the default settings, which included 25% minimal sequence coverage of the BLAST hits and 30% minimal amino acid sequence identity of BLAST hits. The maximum distance between genes in locus was increased to 40 kb (considering that *vlgs* inside a BGC might be separated by some other genes—such as in A47934 BGC [48]). Two input files were used as queries for the MultiGeneBlast search: one included *van* genes from *Str. coelicolor—SCO3589-3590-3594-3595-3596* (*vanSRHAX*); and the other included balhimycin BGC-situated *van* genes from *Am. balhimycina—DMA12_00360, DMA12_00365* and *DMA12_00370* (*vanS*, *vanR* and *vanY* respectively). The first input files allowed to detect *vanHAX* orthologues co-localized with the *vanRS-*like regulatory pairs, while the second helped to detect cases when the *vanY-*like genes were co-localized with *vanRS* but lacked other *van* genes in their genetic neighborhood. MultiGeneBlast outputs were then carefully examined, and the amino acid sequences of the proteins coded with the so-identified *vlgs* were extracted. The information about these *vlgs*, including the corresponding protein accession numbers, nucleic acid accession numbers and taxa, are summarized in Supplementary Excel Table S2. To refine this initial screening, chosen sets of genomes for each order (highlighted in red in Supplementary Excel Table S2) were manually reexamined for *vlgs* using BLASTP [74] with SCO3589 (VanS), SCO3590 (VanR), SCO3592 (VanJ), SCO3593 (VanK), SCO3594 (VanH), SCO3595 (VanA), SCO3596 (VanX)*,* CAG25753 (VanY) and ELS50663 (VanZ) as queries. These last sets of genomes were chosen to cover all the genera within a certain order and to include all the genomes carrying known and putative Type I–V GPA BGCs as well as *feg*-like BGCs. Selected hits were tested for orthology with queries using the Reciprocal Best Hit BLAST approach. Information received here was used to build Figure 2, Figure 3, Figure 4, Figure 5, Figure 6, Figure 7, Figure 8 and Figure 9.

### 4.3. Search for Putative GPA-like BGCs

The MultiGeneBlast tool [73] was used to screen all the genome assemblies for GPA-like BGCs. We utilized the same offline custom databases created for *vlgs* screening. MultiGeneBlast was also run in “homology” mode with the default settings; however, the maximum distance between genes in one locus was increased to 60 kb. An input file was composed of teicoplanin BGC [75] genes—tei4*, tei8*, tei15*, tei17*, tei23*, tei24*, tei28* and tei29*—coding for an ABC transporter, teicoplanin halogenase, StrR-like transcriptional regulator, prephenate dehydrogenase, l-4-hydroxyphenylglycine biosynthesis enzyme HpgT, type III polyketide synthase DpgA (involved in the biosynthesis of both l-4-hydroxyphenylglycine and l-3,5-dihydroxyphenylglycine), HmaS and Hmo (l-3,5-dihydroxyphenylglycine biosynthesis enzymes), respectively. All these genes have their orthologues in BGCs for Type I–V GPAs and in *feg.* MultiGeneBlast outputs were manually examined and the nucleic acid sequences containing MultiGeneBlast hits were applied for upstream antiSMASH [76] analysis. A list of the obtained putative GPA- and *feg-*like BGCs is given in Supplementary Excel Table S2.

### 4.4. Phylogenetic Reconstruction

Since the screening revealed hundreds of *vlgs*, it was difficult to use all the sequences for a comprehensive phylogenetic analysis. Therefore, phylogenies were reconstructed for sets of proteins coded with *vlgs* from the chosen genomes for each order (highlighted in red in Supplementary Excel Table S2). The final protein datasets for the reconstruction of the phylogenies of the VanY-like carboxypeptidases—VanH; VanA and Ddl; VanX and VanX-like dipeptidases; VanY-like carboxypeptidases, VanX and VanX-like dipeptidases; VanR-like regulators; and VanS-like sensor histidine kinases—are given in Supplementary FASTA Files S1–S7, respectively. Some additional information was coded in the name of each protein sequence to indicate (i) whether this protein is BGC-encoded or not; and (ii) whether this protein is coded with an ‘orphan’ gene or the corresponding gene is co-localized with other *vlgs.* For instance, “VanYncs-HAXRS_Tt_sp_HY188” indicates that this is VanY-like non-BGC-encoded peptidase, with the corresponding gene co-localized with *vanHAXRS*, coming from *Tomitella* sp. HY188.

The Mega X [71] package was used to perform the phylogenetic reconstructions. On the road to the representative phylogenetic tree, we always followed the next algorithm. First, multiple amino acid sequence alignments for each dataset were generated using Muscle; the obtained alignments were manually curated to preserve as much meaningful data as possible. Then the curated multiple sequence alignments were analyzed using the Mega X model finder to discover the most appropriate evolutionary models, and the best-scoring models were applied to generate the Maximum Likelihood phylogenies for each protein dataset. A similar approach was used to generate the 16S rRNA gene phylogenetic trees, which can be found in Figure 2, Figure 3, Figure 4 and Figure 5. Final topologies of either the protein or gene trees were derived from 500 bootstraps.

## Figures and Tables

**Table 1:** List of orders belonging to the *Actinobacteria* phylum, which genome assemblies were available in GenBank at the moment of this work preparation (April 2020, Supplementary Excel File S1) and a summary of the *vlgs* found in them.

Order	Number of Genome Assemblies Analyzed	Number of Genome Assembling Containing at Least One *Vlg*	Occurrence (%)	*vanY*-like	*vanR*-like	*vanS*-like	*vanH*	*vanA*	*vanX*
*Acidimicrobiales*	216	0	0	-	-	-	-	-	-
*Actinomycetales*	223	1	0.45	1	1	1	-	-	-
*Actinopolysporales*	10	0	0	-	-	-	-	-	-
*Bifidobacteriales*	1028	0	0	-	-	-	-	-	-
*candidatus* Actinomarinales	214	0	0	-	-	-	-	-	-
*candidatus* Nanopelagicales	26	1	3.85	1	-	-	-	-	-
*Catenulisporales*	3	1	34	1	1	1	1	1	1
*Coriobacteriales*	217	2	0.9	1	2	2	2	2	2
*Corynebacteriales*	707	110	15.6	122	23	25	14	14	14
*Cryptosporangiales*	3	2	67	-	1	1	1	1	1
*Eggerthellales*	106	1	0.9	-	1	1	1	1	1
*Egibacterales*	3	0	0	-	-	-	-	-	-
*Frankiales*	46	7	15	-	6	7	8	8	8
*Gaiellales*	3	0	0	-	-	-	-	-	-
*Geodermatophilales*	60	11	18	12	10	9	-	-	-
*Glycomycetales*	12	11	92	10	10	10	8	8	8
*Jiangellales*	11	10	91	12	11	10	7	7	7
*Kineosporiales*	12	1	8.4	1	1	1	-	-	-
*Micrococcales*	1741	15	0.86	2	13	13	15	15	14
*Micromonosporales*	200	83	42	14	86	88	5	57	56
*Nakamurellales*	6	6	100	6	1	1	-	-	-
*Nitriliruptorales*	6	1	17	1	-	-	-	-	-
*Propionibacteriales*	593	16	2.7	13	16	16	16	16	16
*Pseudonocardiales*	243	135	56	141	129	139	105	105	105
*Rubrobacterales*	8	1	12.5	2	1	1	-	-	-
*Solirubrobacterales*	45	4	8.9	6	6	6	2	2	2
*Streptomycetales*	1138	418	37	126	414	429	93	92	94
*Streptosporangiales*	228	63	28	52	67	63	7	7	6

## Data Availability

All the data are available from the corresponding author upon reasonable request.

## Supplementary Materials

The following are available online at https://www.mdpi.com/article/10.3390/antibiotics10121533/s1: Supplementary Excel File S1, Summary of actinobacterial genome assemblies with corresponding nucleic acid sequences analyzed in this work. Supplementary Excel File S2, Accession numbers and locations of Van-like proteins and GPA BGCs, discovered in this work for actinobacteria. Supplementary Excel File S3, Summary of *Bacillales* genome assemblies analyzed with corresponding nucleic acid sequences; accession numbers of Van-like proteins discovered for *Bacillales* spp. Supplementary FASTA Files S1–S7, containing protein datasets for reconstructing phylogenies of different Van-like proteins, see Section 4.4. Supplementary Tables and Figures File, containing ESM Table S1, Abbreviations of *Actinobacteria* and *Bacillales* genera names, used throughout the work; ESM Table S2, List of transposases from known MGEs carrying *vlgs* used for comparing with the transposases encoded within *Atopobium minutum* 10063974, *Enterorhabdus mucosicola* NM66_B29 and *Parvibacter caecicola* DSM 22242 MGEs; ESM Figure S1, Defining the genes annotated within previously unannotated regions flanking the CA878 GPA BGC; ESM Figure S2, Defining the genes annotated within previously unannotated flanks of CA915 and CA37 GPA BGCs; ESM Figure S3, Phylogenetic tree showing the overall phylogeny of VanY-like M15B carboxypeptidase dataset; ESM Figure S4, Expanded clade corresponding to the Y1 cluster from ESM Figure S3; ESM Figure S5, Expanded clade corresponding to the Y2 cluster from ESM Figure S3; ESM Figure S6, Expanded clades corresponding to the Y3 and Y4 clusters from ESM Figure S3; ESM Figure S7, Expanded clade corresponding to the Y5 cluster from ESM Figure S3; ESM Figure S8, Phylogenetic tree showing the overall phylogeny of VanH-dataset; ESM Figure S9, Phylogenetic tree showing the overall phylogeny of the dataset composed with VanA proteins and Ddl-ligases; ESM Figure S10, Expanded clade of Ddl-ligases coded in putative pdx-operons; ESM Figure S11, Expanded clade containing VanA-ligases from low G-C Gram-positive bacteria; ESM Figure S12, Expanded crown group of VanA-phylogenetic tree; ESM Figure S13, Phylogenetic tree showing the overall phylogeny of the dataset composed with VanX-like proteins; ESM Figure S14, Expanded version of the crown group of actinobacterial VanX M15D dipeptidases; ESM Figure S15, Phylogeny of combined datasets for VanY-like M15B carboxypeptidases and VanX M15D dipeptidases, including also VanXY-proteins; ESM Figure S16, Expanded version of the VR1 cluster from the phylogenetic tree of actinobacterial VanR-like response regulators; ESM Figure S17, Expanded version of the VS2 cluster from the phylogenetic tree of actinobacterial VanS-like sensor histidine kinases; ESM Figure S18, Expanded versions of the VR2 and VR3 clusters from the phylogenetic tree of actinobacterial VanR-like response regulators; ESM Figure S19, Expanded version of the VS1 cluster from the phylogenetic tree of actinobacterial VanS-like sensor histidine kinases; ESM Figure S20, Expanded version of the Dbv6-like cluster from the phylogenetic tree of actinobacterial VanR-like response regulators; ESM Figure S21, Expanded version of the Dbv22-like cluster from the phylogenetic tree of actinobacterial VanS-like sensor histidine kinases; ESM Figure S22, Expanded version of the VR4 cluster from the phylogenetic tree of actinobacterial VanR-like response regulators; ESM Figure S23, Expanded version of the VS3 cluster from the phylogenetic tree of actinobacterial VanS-like sensor histidine kinases; ESM Figure S24, Expanded version of the clade corresponding to the crown group of bacterial VanR response regulators; ESM Figure S25, Expanded version of the crown group of bacterial VanS-like sensor histidine kinases; ESM Figure S26, Expanded versions of the VR5-7 clusters of the phylogenetic tree from ESM Figure S24; ESM Figure S27, Expanded version of the VS4 cluster of the phylogenetic tree from ESM Figure S25; ESM Figure S28, Expanded version of the VS5 cluster of the phylogenetic tree from ESM Figure S25; ESM Figure S29, Expanded version of the VS6 cluster of the phylogenetic tree from ESM Figure S25; ESM Figure S30, Expanded version of the VS7 cluster of the phylogenetic tree from ESM Figure S25; ESM Figure S31, Expanded version of the VS8 cluster of the phylogenetic tree from ESM Figure S25.

## References

[1] Fleming A. (2001). On the antibacterial action of cultures of a penicillium, with special reference to their use in the isolation of B. influenzae. 1929. Bull. World Health Organ..

[2] O’ Neil J. Review on Antibiotic resistance. Antimicrobial resistance: Tackling a crisis for the health and wealth of nations. *Health Wealth Nations*
**2014**, 1–16. https://wellcomecollection.org/works/rdpck35v.

[3] Ghosh S., Bornman C., Zafer M.M. (2021). Antimicrobial resistance threats in the emerging COVID-19 pandemic: Where do we stand?. J. Infect. Public Health.

[4] Marcone G.L., Binda E., Berini F., Marinelli F. (2018). Old and new glycopeptide antibiotics: From product to gene and back in the post-genomic era. Biotechnol. Adv..

[5] Nicolaou K.C., Boddy C.N.C., Bräse S., Winssinger N. (1999). Chemistry, biology, and medicine of the glycopeptide antibiotics. Angew. Chem. Int. Ed..

[6] Sarkar P., Yarlagadda V., Ghosh C., Haldar J. (2017). A review on cell wall synthesis inhibitors with an emphasis on glycopeptide antibiotics. Medchemcomm.

[7] Vollmer W., Blanot D., De Pedro M.A. (2008). Peptidoglycan structure and architecture. FEMS Microbiol. Rev..

[8] Perkins H.R. (1969). Specificity of combination between mucopeptide precursors and vancomycin or ristocetin. Biochem. J..

[9] Nitanai Y., Kikuchi T., Kakoi K., Hanamaki S., Fujisawa I., Aoki K. (2009). Crystal structures of the complexes between vancomycin and cell-wall precursor analogs. J. Mol. Biol..

[10] Williams D.H. (1996). The glycopeptide story-how to kill the deadly “Superbugs”. Nat. Prod. Rep..

[11] Marschall E., Cryle M.J., Tailhades J. (2019). Biological, chemical, and biochemical strategies for modifying glycopeptide antibiotics. J. Biol. Chem..

[12] Parenti F., Cavalleri B. (1989). Proposal to name the vancomycin-ristocetin like glycopeptides as dalbaheptides. J. Antibiot..

[13] Yushchuk O., Binda E., Marinelli F. (2020). Glycopeptide antibiotic resistance genes: Distribution and function in the producer actinomycetes. Front. Microbiol..

[14] Binda E., Marinelli F., Marcone G.L. (2014). Old and new glycopeptide antibiotics: Action and resistance. Antibiotics.

[15] Arthur M., Quintiliani R.J. (2001). Regulation of VanA-and VanB-type glycopeptide resistance in enterococci. Antimicrob. Agents Chemother..

[16] Arthur M., Molinas C., Bugg T.D.H., Wright G.D., Walsh C.T., Courvalin P. (1992). Evidence for *in vivo* incorporation of d-lactate into peptidoglycan precursors of vancomycin-resistant enterococci. Antimicrob. Agents Chemother..

[17] Stegmann E., Frasch H.J., Kilian R., Pozzi R. (2015). Self-resistance mechanisms of actinomycetes producing lipid II-targeting antibiotics. Int. J. Med. Microbiol..

[18] Waglechner N., McArthur A.G., Wright G.D. (2019). Phylogenetic reconciliation reveals the natural history of glycopeptide antibiotic biosynthesis and resistance. Nat. Microbiol..

[19] Culp E.J., Waglechner N., Wang W., Fiebig-Comyn A.A., Hsu Y.P., Koteva K., Sychantha D., Coombes B.K., Van Nieuwenhze M.S., Brun Y.V. (2020). Evolution-guided discovery of antibiotics that inhibit peptidoglycan remodelling. Nature.

[20] Xu M., Wang W., Waglechner N., Culp E.J., Guitor A.K., Wright G.D. (2020). GPAHex-A synthetic biology platform for Type IV–V glycopeptide antibiotic production and discovery. Nat. Commun..

[21] Mitchell S.J., Verma D., Griswold K.E., Bailey-Kellogg C. (2021). Building blocks and blueprints for bacterial autolysins. PLoS Comput. Biol..

[22] Cheng A.V., Wuest W.M. (2020). Phylogeny-guided approach yields glycopeptides with unique action. Trends Pharmacol. Sci..

[23] Gonsior M., Mühlenweg A., Tietzmann M., Rausch S., Poch A., Süssmuth R.D. (2015). Biosynthesis of the peptide antibiotic feglymycin by a linear nonribosomal peptide synthetase mechanism. Chem. Biol. Chem..

[24] Hong H.J., Paget M.S.B., Buttner M.J. (2002). A signal transduction system in *Streptomyces coelicolor* that activates expression of a putative cell wall glycan operon in response to vancomycin and other cell wall-specific antibiotics. Mol. Microbiol..

[25] Patel R., Piper K., Cockerill F.R., Steckelberg J.M., Yousten A.A. (2000). The biopesticide *Paenibacillus popilliae* has a vancomycin resistance gene cluster homologous to the enterococcal VanA vancomycin resistance gene cluster. Antimicrob. Agents Chemother..

[26] Fontana R., Ligozzi M., Pedrotti C., Padovani E.M., Cornaglia G. (1997). Vancomycin-resistant *Bacillus circulans* carrying the *vanA* gene responsible for vancomycin resistance in enterococci. Eur. J. Clin. Microbiol. Infect. Dis..

[27] Marshall C.G., Broadhead G., Leskiw B.K., Wright G.D. (1997). d-Ala-d-Ala ligases from glycopeptide antibiotic-producing organisms are highly homologous to the enterococcal vancomycin-resistance ligases VanA and VanB. Proc. Natl. Acad. Sci. USA.

[28] Aminov R.I., Mackie R.I. (2007). Evolution and ecology of antibiotic resistance genes. FEMS Microbiol. Lett..

[29] One Health. https://www.who.int/news-room/q-a-detail/one-health.

[30] Hutchings M.I., Hong H.J., Buttner M.J. (2006). The vancomycin resistance VanRS two-component signal transduction system of *Streptomyces coelicolor*. Mol. Microbiol..

[31] Hong H.J., Hutchings M.I., Neu J.M., Wright G.D., Paget M.S.B., Buttner M.J. (2004). Characterization of an inducible vancomycin resistance system in *Streptomyces coelicolor* reveals a novel gene (*vanK*) required for drug resistance. Mol. Microbiol..

[32] Adamek M., Alanjary M., Sales-Ortells H., Goodfellow M., Bull A.T., Winkler A., Wibberg D., Kalinowski J., Ziemert N. (2018). Comparative genomics reveals phylogenetic distribution patterns of secondary metabolites in *Amycolatopsis* species. BMC Genom..

[33] Shawky R.M., Puk O., Wietzorrek A., Pelzer S., Takano E., Wohlleben W., Stegmann E. (2007). The border sequence of the balhimycin biosynthesis gene cluster from *Amycolatopsis balhimycina* contains *bbr*, encoding a StrR-like pathway-specific regulator. J. Mol. Microbiol. Biotechnol..

[34] Gudeta D.D., Moodley A., Bortolaia V., Guardabassi L. (2014). VanO, a new glycopeptide resistance operon in environmental *Rhodococcus equi* isolates. Antimicrob. Agents Chemother..

[35] Mengin-Lecreulx D., Texier L., Rousseau M., Van Heijenoort J. (1991). The *murG* gene of *Escherichia coli* codes for the UDP-*N*-acetylglucosamine: *N*-acetylmuramyl-(pentapeptide) pyrophosphoryl-undecaprenol *N*-acetylglucosamine transferase involved in the membrane steps of peptidoglycan synthesis. J. Bacteriol..

[36] Banik J.J., Craig J.W., Calle P.Y., Brady S.F. (2010). Tailoring enzyme-rich environmental DNA clones: A source of enzymes for generating libraries of unnatural natural products. J. Am. Chem. Soc..

[37] Goldstein B.P., Selva E., Gastaldo L., Berti M., Pallanza R., Ripamonti F., Ferrari P., Denaro M., Arioli V., Cassani G. (1987). A40926, a new glycopeptide antibiotic with anti-*Neisseria* activity. Antimicrob. Agents Chemother..

[38] Binda E., Marcone G.L., Pollegioni L., Marinelli F. (2012). Characterization of VanYn, a novel d, d-peptidase/d, d-carboxypeptidase involved in glycopeptide antibiotic resistance in *Nonomuraea* sp. ATCC 39727. FEBS J..

[39] Binda E., Marcone G.L., Berini F., Pollegioni L., Marinelli F. (2013). *Streptomyces* spp. as efficient expression system for a d,d-peptidase/d,d-carboxypeptidase involved in glycopeptide antibiotic resistance. BMC Biotechnol..

[40] Yushchuk O., Vior N.M., Andreo-Vidal A., Berini F., Rückert C., Busche T., Binda E., Kalinowski J., Truman A.W., Marinelli F. (2021). Genomic-led discovery of a novel glycopeptide antibiotic by *Nonomuraea coxensis* DSM 45129. ACS Chem. Biol..

[41] Nazari B., Forneris C.C., Gibson M.I., Moon K., Schramma K.R., Seyedsayamdost M.R. (2017). *Nonomuraea* sp. ATCC 55076 harbours the largest actinomycete chromosome to date and the kistamicin biosynthetic gene cluster. Medchemcomm.

[42] Yushchuk O., Ostash B., Truman A.W., Marinelli F., Fedorenko V. (2020). Teicoplanin biosynthesis: Unraveling the interplay of structural, regulatory, and resistance genes. Appl. Microbiol. Biotechnol..

[43] Bardone M.R., Paternoster M., Coronelli C. (1978). Teichomycins, new antibiotics from *Actinoplanes teichomyceticus* nov. sp. II. Extraction and chemical characterization. J. Antibiot..

[44] Yim G., Kalan L., Koteva K., Thaker M.N., Waglechner N., Tang I., Wright G.D. (2014). Harnessing the synthetic capabilities of glycopeptide antibiotic tailoring enzymes: Characterization of the UK-68, 597 biosynthetic cluster. Chem. Biol. Chem..

[45] Debono M., Merkel K.E., Molloy R.M., Barnhart M., Prestí E., Hunt A.H., Hamill R.L. (1984). Actaplanin, new glycopeptide antibiotics produced by *Actinoplanes missouriensis* the isolation and preliminary chemical characterization of actaplanin. J. Antibiot..

[46] Yushchuk O., Homoniuk V., Ostash B., Marinelli F., Fedorenko V. (2020). Genetic insights into the mechanism of teicoplanin self-resistance in *Actinoplanes teichomyceticus*. J. Antibiot..

[47] Owen J.G., Reddy B.V.B., Ternei M.A., Charlop-Powers Z., Calle P.Y., Kim J.H., Brady S.F. (2013). Mapping gene clusters within arrayed metagenomic libraries to expand the structural diversity of biomedically relevant natural products. Proc. Natl. Acad. Sci. USA.

[48] Pootoolal J., Thomas M.G., Marshall C.G., Neu J.M., Hubbard B.K., Walsh C.T., Wright G.D. (2002). Assembling the glycopeptide antibiotic scaffold: The biosynthesis of A47934 from *Streptomyces toyocaensis* NRRL15009. Proc. Natl. Acad. Sci. USA.

[49] Thaker M.N., Wang W., Spanogiannopoulos P., Waglechner N., King A.M., Medina R., Wright G.D. (2013). Identifying producers of antibacterial compounds by screening for antibiotic resistance. Nat. Biotechnol..

[50] Hong H.J., Hutchings M.I., Hill L.M., Buttner M.J. (2005). The role of the novel fem protein VanK in vancomycin resistance in *Streptomyces coelicolor*. J. Biol. Chem..

[51] Pathom-Aree W., Nogi Y., Sutcliffe I.C., Ward A.C., Horikoshi K., Bull A.T., Goodfellow M. (2006). *Williamsia marianensis* sp. nov., a novel actinomycete isolated from the Mariana Trench. Int. J. Syst. Evol. Microbiol..

[52] Hegstad K., Mikalsen T., Coque T.M., Werner G., Sundsfjord A. (2010). Mobile genetic elements and their contribution to the emergence of antimicrobial resistant *Enterococcus faecalis* and *Enterococcus faecium*. Clin. Microbiol. Infect..

[53] Garnier F., Taourit S., Glaser P., Courvalin P., Galimand M. (2000). Characterization of transposon Tn1549, conferring VanB-type resistance in *Enterococcus* spp.. Microbiol..

[54] Handwerger S., Skoble J. (1995). Identification of chromosomal mobile element conferring high-level vancomycin resistance in *Enterococcus faecium*. Antimicrob. Agents Chemother..

[55] Reynolds P.E., Ambur O.H., Casadewall B., Courvalin P. (2001). The VanYD dd-carboxypeptidase of *Enterococcus faecium* BM4339 is a penicillin-binding protein. Microbiol..

[56] Hopwood D.A., Wright H.M. (1972). Transformation in *Thermoactinomyces vulgaris*. J. Gen. Microbiol..

[57] Meziane-Cherif D., Stogios P.J., Evdokimova E., Savchenko A., Courvalin P. (2014). Structural basis for the evolution of vancomycin resistance d,d-peptidases. Proc. Natl. Acad. Sci. USA.

[58] Rawlings N.D., Waller M., Barrett A.J., Bateman A. (2014). MEROPS: The database of proteolytic enzymes, their substrates and inhibitors. Nucleic Acids Res..

[59] Charlier P., Wery J.-P., Dideberg O., Frère J.-M. (2006). *Streptomyces albus* G d-Ala-d-Ala carboxypeptidase. Handb. Met..

[60] Marshall C.G., Lessard I.A.D., Park I.S., Wright G.D. (1998). Glycopeptide antibiotic resistance genes in glycopeptide-producing organisms. Antimicrob. Agents Chemother..

[61] Bussiere D.E., Pratt S.D., Katz L., Severin J.M., Holzman T., Park C.H. (1998). The structure of VanX reveals a novel amino-dipeptidase involved in mediating transposon-based vancomycin resistance. Mol. Cell.

[62] Marcone G.L., Beltrametti F., Binda E., Carrano L., Foulston L., Hesketh A., Bibb M., Marinelli F. (2010). Novel mechanism of glycopeptide resistance in the A40926 producer *Nonomuraea* sp. ATCC 39727. Antimicrob. Agents Chemother..

[63] Kilian R., Frasch H.J., Kulik A., Wohlleben W., Stegmann E. (2016). The VanRS homologous two-component system VnlRS_Ab_ of the glycopeptide producer *Amycolatopsis balhimycina* activates transcription of the *vanHAX_Sc_* genes in *Streptomyces coelicolor*, but not in *A. balhimycina*. Microb. Drug Resist..

[64] Mainardi J.-L., Villet R., Bugg T.D., Mayer C., Arthur M. (2008). Evolution of peptidoglycan biosynthesis under the selective pressure of antibiotics in Gram-positive bacteria. FEMS Microbiol. Rev..

[65] Podmore A.H.B., Reynolds P.E. (2002). Purification and characterization of VanXYc, a d,d-dipeptidase/d,d-carboxypeptidase in vancomycin-resistant *Enterococcus gallinarum* BM4174. Eur. J. Biochem..

[66] Alduina R., Tocchetti A., Costa S., Ferraro C., Cancemi P., Sosio M., Donadio S. (2020). A two-component regulatory system with opposite effects on glycopeptide antibiotic biosynthesis and resistance. Sci. Rep..

[67] D’Argenio V., Petrillo M., Pasanisi D., Pagliarulo C., Colicchio R., Talà A., De Biase M.S., Zanfardino M., Scolamiero E., Pagliuca C. (2016). The complete 12 Mb genome and transcriptome of *Nonomuraea gerenzanensis* with new insights into its duplicated “magic” RNA polymerase. Sci. Rep..

[68] Yushchuk O., Andreo-Vidal A., Marcone G.L., Bibb M., Marinelli F., Binda E. (2020). New molecular tools for regulation and improvement of A40926 glycopeptide antibiotic production in *Nonomuraea gerenzanensis* ATCC 39727. Front. Microbiol..

[69] Rioseras B., Yaguë P., López-Garciá M.T., Gonzalez-Quinõnez N., Binda E., Marinelli F., Manteca A. (2016). Characterization of SCO4439, a d-alanyl-d-alanine carboxypeptidase involved in spore cell wall maturation, resistance, and germination in *Streptomyces Coelicolor*. Sci. Rep..

[70] Kearse M., Moir R., Wilson A., Stones-Havas S., Cheung M., Sturrock S., Buxton S., Cooper A., Markowitz S., Duran C. (2012). Geneious Basic: An integrated and extendable desktop software platform for the organization and analysis of sequence data. Bioinformatics.

[71] Kumar S., Stecher G., Li M., Knyaz C., Tamura K. (2018). MEGA X: Molecular evolutionary genetics analysis across computing platforms. Mol. Biol. Evol..

[72] Sievers F., Higgins D.G. (2014). Clustal Omega. Curr. Protoc. Bioinform..

[73] Medema M.H., Takano E., Breitling R. (2013). Detecting sequence homology at the gene cluster level with MultiGeneBlast. Mol. Biol. Evol..

[74] Altschul S.F., Gish W., Miller W., Myers E.W., Lipman D.J. (1990). Basic local alignment search tool. J. Mol. Biol..

[75] Li T.L., Huang F., Haydock S.F., Mironenko T., Leadlay P.F., Spencer J.B. (2004). Biosynthetic gene cluster of the glycopeptide antibiotic teicoplanin: Characterization of two glycosyltransferases and the key acyltransferase. Chem. Biol..

[76] Blin K., Shaw S., Steinke K., Villebro R., Ziemert N., Lee S.Y., Medema M.H., Weber T. (2019). AntiSMASH 5.0: Updates to the secondary metabolite genome mining pipeline. Nucleic Acids Res..

