# Supplementary material for: Genomic Insights into the Distribution and Phylogeny of Glycopeptide Resistance Determinants within the Actinobacteria Phylum
Source: Antibiotics (Basel). 2021 Dec 14;10(12):1533. doi: 10.3390/antibiotics10121533 (PMC8698665; doi:10.3390/antibiotics10121533)
Supplement: Supplementary file 1 [file antibiotics-10-01533-s001.zip › Supplementary Tables and Figures File.pdf]

Supplementary Tables and Figures File

Genomic insights into the distribution and phylogeny of glycopeptide resistance determinants within the Actinobacteria phylum

Andres Andreo-Vidal [1], Elisa Binda [1], Victor Fedorenko [2], Flavia Marinelli [1] and Oleksandr Yushchuk [1,2*]

[1] Department of Biotechnology and Life Sciences, University of Insubria, Varese, Italy.

[2] Department of Genetics and Biotechnology, Ivan Franko National University of Lviv, Lviv, Ukraine.

Inventory of Supplementary Tables and Figures File

Contents	Page
**ESM Table S1.** Abbreviations of Actinobacteria and *Bacillales* genera names, used throughout the work.	**4**
**ESM Table S2.** List of transposases from known MGEs carrying *vlgs.*	**6**
**ESM Figure S1.** Defining the genes annotated within previously unannotated regions flanking the CA878 GPA BGC.	**7**
**ESM Figure S2.** Defining the genes annotated within previously unannotated flanks of CA915 and CA37 GPA BGCs.	**8**
**ESM Figure S3.** Phylogenetic tree showing the overall phylogeny of VanY-like M15B carboxypeptidase dataset.	**9**
**ESM Figure S4.** Expanded clade corresponding to the Y1 cluster from ESM Figure S3.	**10**
**ESM Figure S5.** Expanded clade corresponding to the Y2 cluster from ESM Figure S3.	**11**
**ESM Figure S6.** Expanded clades corresponding to the Y3 and Y4 clusters from ESM Figure S3.	**12**
**ESM Figure S7.** Expanded clade corresponding to the Y5 cluster from ESM Figure S3.	**13**
**ESM Figure S8.** Phylogenetic tree showing the overall phylogeny of VanH-dataset.	**14-15**
**ESM Figure S9.** Phylogenetic tree showing the overall phylogeny of the dataset composed with VanA proteins and Ddl-ligases.	**16**
**ESM Figure S10.** Expanded clade of Ddl-ligases coded in putative *pdx*-operons.	**17**
**ESM Figure S11.** Expanded clade containing VanA-ligases from low G-C Gram-positive bacteria.	**17**
**ESM Figure S12.** Expanded crown group of VanA-phylogenetic tree.	**18-19**
**ESM Figure S13.** Phylogenetic tree showing the overall phylogeny of the dataset composed with VanX-like proteins.	**20**
**ESM Figure S14.** Expanded version of the crown group of actinobacterial VanX M15D dipeptidases.	**21-22**
**ESM Figure S15.** Phylogeny of combined datasets for VanY-like M15B carboxypeptidases and VanX M15D dipeptidases, including also VanXY-proteins.	**23**
**ESM Figure S16.** Expanded version of the VR1 cluster from the phylogenetic tree of actinobacterial VanR-like response regulators.	**24**
**ESM Figure S17.** Expanded version of the VS2 cluster from the phylogenetic tree of actinobacterial VanS-like sensor histidine kinases.	**25**
**ESM Figure S18.** Expanded versions of the VR2 and VR3 clusters from the phylogenetic tree of actinobacterial VanR-like response regulators.	**26**
**ESM Figure S19.** Expanded version of the VS1 cluster from the phylogenetic tree of actinobacterial VanS-like sensor histidine kinases.	**27**
**ESM Figure S20.** Expanded version of the Dbv6-like cluster from the phylogenetic tree of actinobacterial VanR-like response regulators.	**28**
**ESM Figure S21.** Expanded version of the Dbv22-like cluster from the phylogenetic tree of actinobacterial VanS-like sensor histidine kinases.	**29**
**ESM Figure S22.** Expanded version of the VR4 cluster from the phylogenetic tree of actinobacterial VanR-like response regulators.	**30**

**Contents**	**Page**
**ESM Figure S23.** Expanded version of the VS3 cluster from the phylogenetic tree of actinobacterial VanS-like sensor histidine kinases.	**30**
**ESM Figure S24.** Expanded version of the clade corresponding to the crown group of bacterial VanR response regulators.	**31-32**
**ESM Figure S25.** Expanded version of the crown group of bacterial VanS-like sensor histidine kinases.	**33-34**
**ESM Figure S26.** Expanded versions of the VR5-7 clusters of the phylogenetic tree from ESM Figure S24.	**35**
**ESM Figure S27.** Expanded version of the VS4 cluster of the phylogenetic tree from ESM Figure S25.	**36**
**ESM Figure S28.** Expanded version of the VS5 cluster of the phylogenetic tree from ESM Figure S25.	**36**
**ESM Figure S29.** Expanded version of the VS6 cluster of the phylogenetic tree from ESM Figure S25.	**37**
**ESM Figure S30.** Expanded version of the VS7 cluster of the phylogenetic tree from ESM Figure S25.	**37**
**ESM Figure S31.** Expanded version of the VS8 cluster of the phylogenetic tree from ESM Figure S25.	**38**
**Supplementary references**	**39**

ESM Table S1. Abbreviations of *Actinobacteria* and *Bacillales* genera names, used throughout the work.

Full genus name:	Abbreviation used in the work:
Agromyces.	*A.*
Actinocrispum	*Ac.*
Aeromicrobium	*Aer.*
Actinokineospora	*Ak.*
Alloactinosynnema	*Al.*
Allonocardiopsis	*All.*
Amycolatopsis	*Am.*
Actinomadura	*Amd.*
Actinophytocola	*Ap.*
Actinoplanes	*Apl.*
Actinopolymorpha	*Apm.*
Atopobium.	*Atp.*
Bacillus	*Bac.*
Blastococcus	*Bc.*
Brevibacillus	*Bbac.*
Baekduia	*Bd.*
cand. Planktophila	*c. Pp.*
Catellatospora	*Cls.*
Diaminobutiricimonas	*Dab.*
Enterorhabdus	*Er.*
Frankia	*F.*
Gordonia	*G.*
Glacibacter	*Gb.*
Geodermathophilus	*Gdp.*
Glycomyces	*Gm.*
Herbihabitans	*H.*
Haloactinospora	*Ha.*
Herbidospora	*Hs.*
Jiangella	*Ja.*
Jishengella	*Js.*
Kutzneria	*K.*
Kibdelosporangium	*Kib.*
Kribbella	*Kr.*
Kitasatospora	*Ksp.*
Lentzea	*L.*
Leifsonia	*Ls.*
Microbispora	*M.*
Mycobacterium	*Mb.*
Mycolicibacterium	*Mcb.*
Murinocardiopsis	*Mcp.*
Modestobacter	*Mdb.*
Micromonospora.	*Mms.*
Nonomuraea	*N.*
Nocardia	*Ncd.*
Nitriliruptoraceae bacterium	*NiB*
Nakamurella	*Nm.*
Nocardiopsis	*Np.*

Full genus name:	Abbreviation used in the work:
Paenibacillus	*Pnb.*
Pseudokineococcus	*Pkc.*
Promicromonospora	*Pms.*
Prauserella	*Pr.*
Pseudonocardia	*Ps.*
Parvibacter	*Pbv.*
Rathayibacter	*Rb.*
Rhodococcus	*Rc.*
Stackebrandtia	*Sb.*
Streptomonospora	*Sms.*
Saccharopolyspora	*Sp.*
Streptosporangium	*Ss.*
Saccharothtix	*St.*
Streptomyces	*Str.*
Tamaricihabitans	*T.*
Thermoactinomyces	*Tam.*
Tsukamurella	*Tm.*
Tomitella	*Tt.*
Verrucosispora	*Vcs.*
Williamsia	*W.*
Xiangella	*Xa.*
Cryptosporangium	*Cs.*

ESM Table S2. List of transposases from known MGEs carrying *vlgs* used for comparing with the transposases encoded within *Atopobium minutum* 10063974, *Enterorhabdus mucosicola* NM66_B29 and *Parvibacter caecicola* DSM 22242 MGEs.

Protein ID:	Name:	Source:	Reference:
AAQ17155	Tn1546 transposase	*Staphylococcus aureus* plasmid pLW043	[1]
AAQ17125	IS431mec transposase	*Staphylococcus aureus* plasmid pLW043	[1]
AAQ17171	Tn552 transposase	*Staphylococcus aureus* plasmid pLW043	[1]
AAB42161	Tn5506 IS1252 transposase	*Enterococcus faecium* plasmid pHKK701	[2]
RNL10277	site-specific integrase	*Parvibacter caecicola* DSM 22242	-
AAB00677	Tn5482 IS3-like transposase	*Enterococcus faecium* insertion sequence IS1216V	[3]
AAB00676	Tn5482 ISS1 homolog transposase	*Enterococcus faecium* insertion sequence IS1216V	[3]
AAO83056	IS256 transposase	*Enterococcus faecalis* V583 plasmid pTEF1	[4]
AAO83057	IS1216 transposase	*Enterococcus faecalis* V583 plasmid pTEF1	[4]
AAF72368	Tn1549 integrase	*Enterococcus faecalis* transposon Tn1549	[5]
AAL27448	Tn6202 Int410 integrase	*Enterococcus faecalis* N00-0410 transposon Tn6202	[6]
AAC44460	Tn1547 transposase	*Enterococcus faecalis* insertion sequence IS16, Tn1547	[7]
EMZ42128	site-specific recombinase/integrase	*Atopobium minutum* 10063974	-
MVX60893	tyrosine-type recombinase/integrase	*Enterorhabdus mucosicola* NM66_B29	-

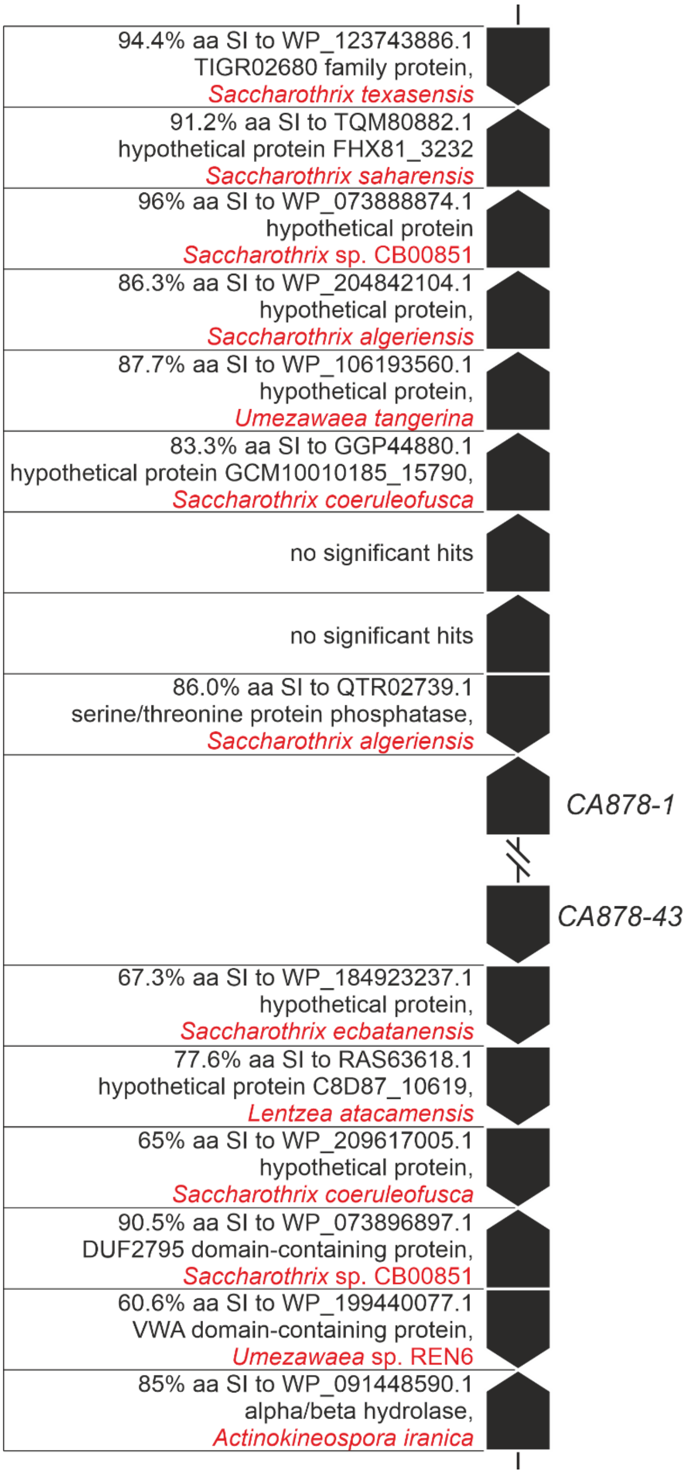

ESM Figure S1. Defining the genes annotated within previously unannotated regions flanking the CA878 GPA BGC (HM486075, genes *CA878-1* to *CA878-43*), coming from metagenomic sample. Majority of newly annotated genes encode proteins which have homologues with highest amino acid sequence identity percentages (aa SI) in *Saccharothrix* spp.

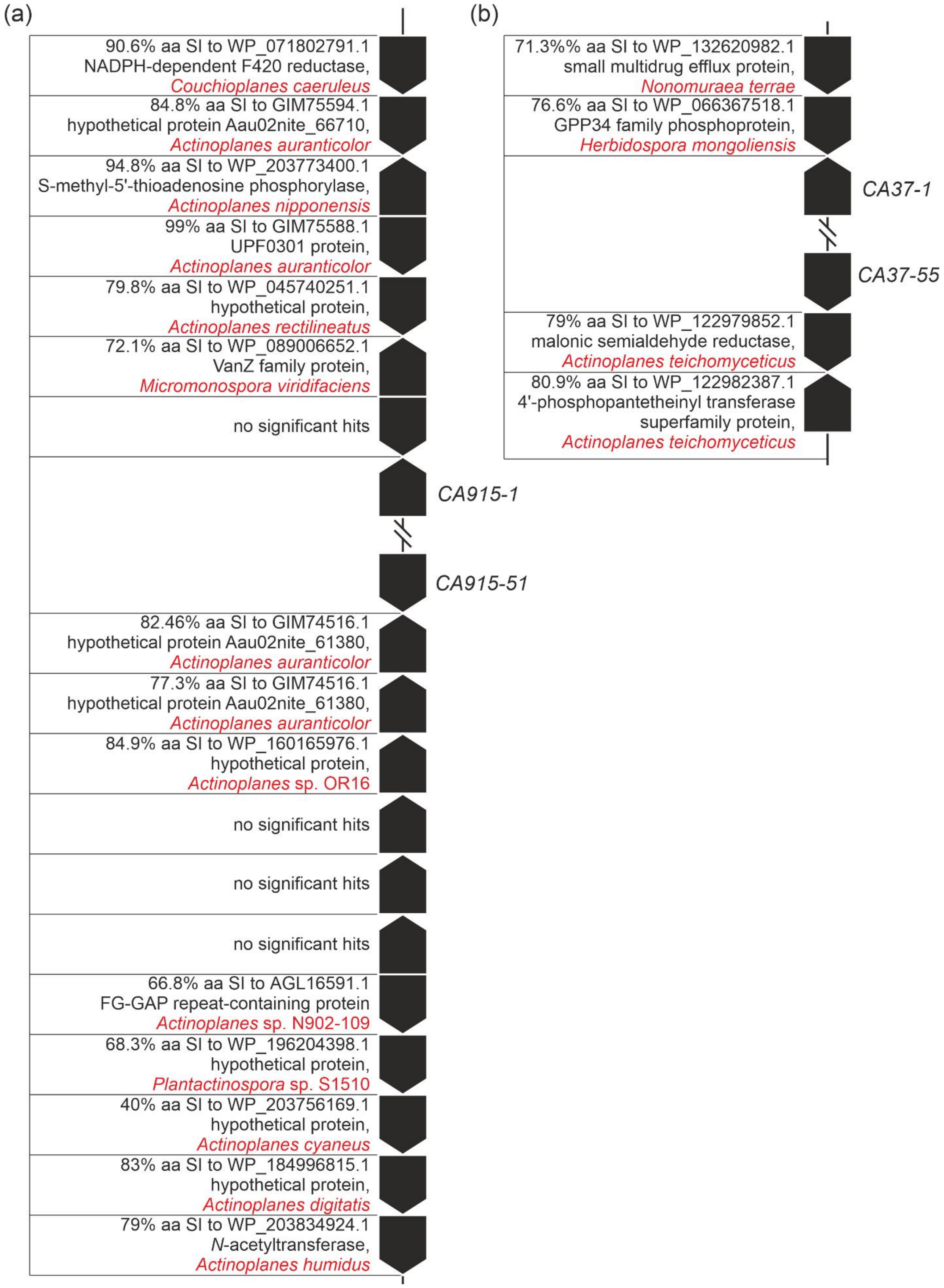

ESM Figure S2. Defining the genes annotated within previously unannotated flanks of (**a**) CA915 (HM486076, genes from *CA915-1* to *CA915-51*) and (**b**) CA37 (HM486074, genes from *CA37-1* to *CA37-55*) GPA BGCs, coming from metagenomic samples. Majority of newly annotated genes encode proteins which have homologues with highest amino acid sequence identity percentages (aa SI) in *Actinoplanes* spp.

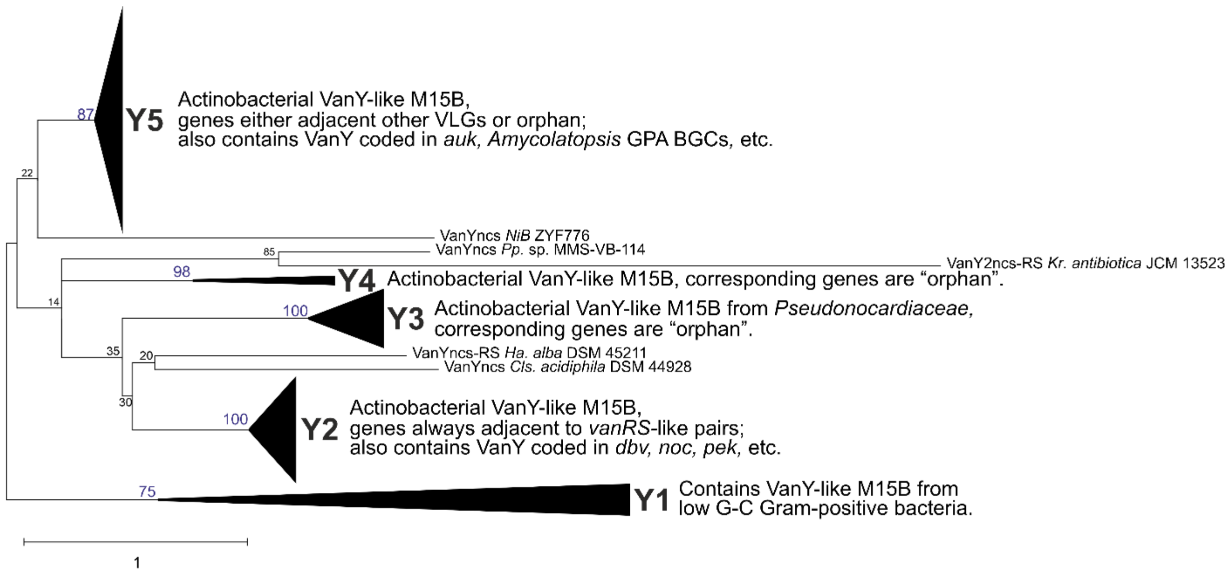

ESM Figure S3. Phylogenetic tree showing the overall phylogeny of VanY-like M15B carboxypeptidase dataset. Five main clusters (Y1-Y5) were collapsed. Expanded clusters are given separately: Y1 in ESM Figure S4; Y2 in ESM Figure S5; Y3 and Y4 in ESM Figure S6; Y5 in ESM Figure S7. Phylogenetic tree was constructed as described in Methods section. “cs/ncs” abbreviations in the label at the tip of each branch here and further means “cluster-situated/non-cluster-situated”. Scale bar represents number of substitutions per site.

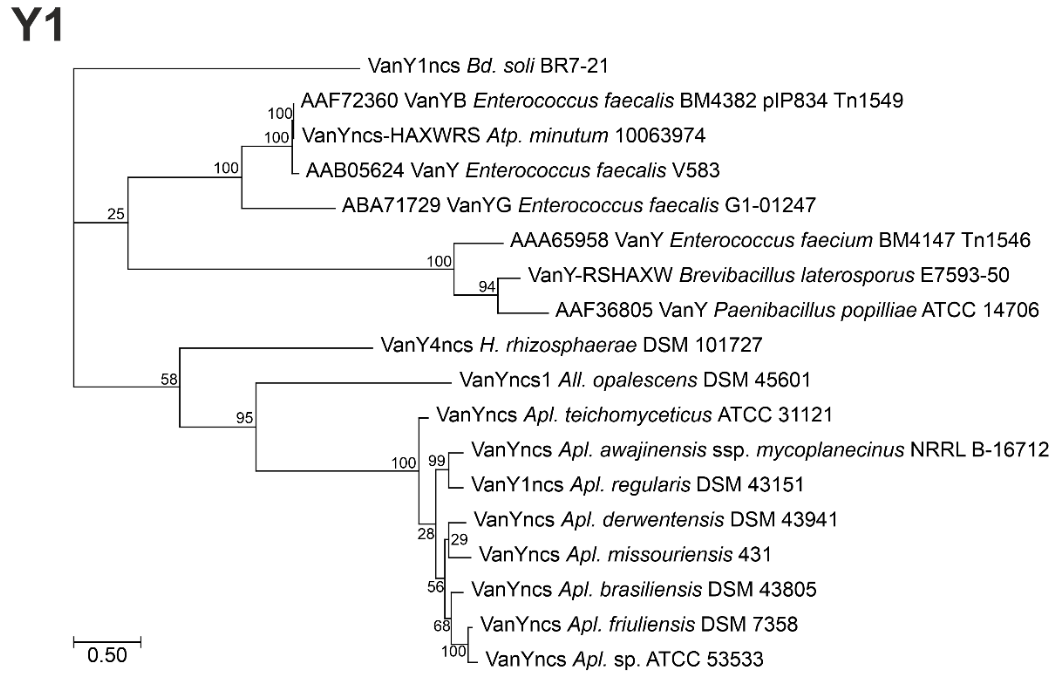

ESM Figure S4. Expanded clade corresponding to the Y1 cluster from ESM Figure S3. Scale bar represents number of substitutions per site.

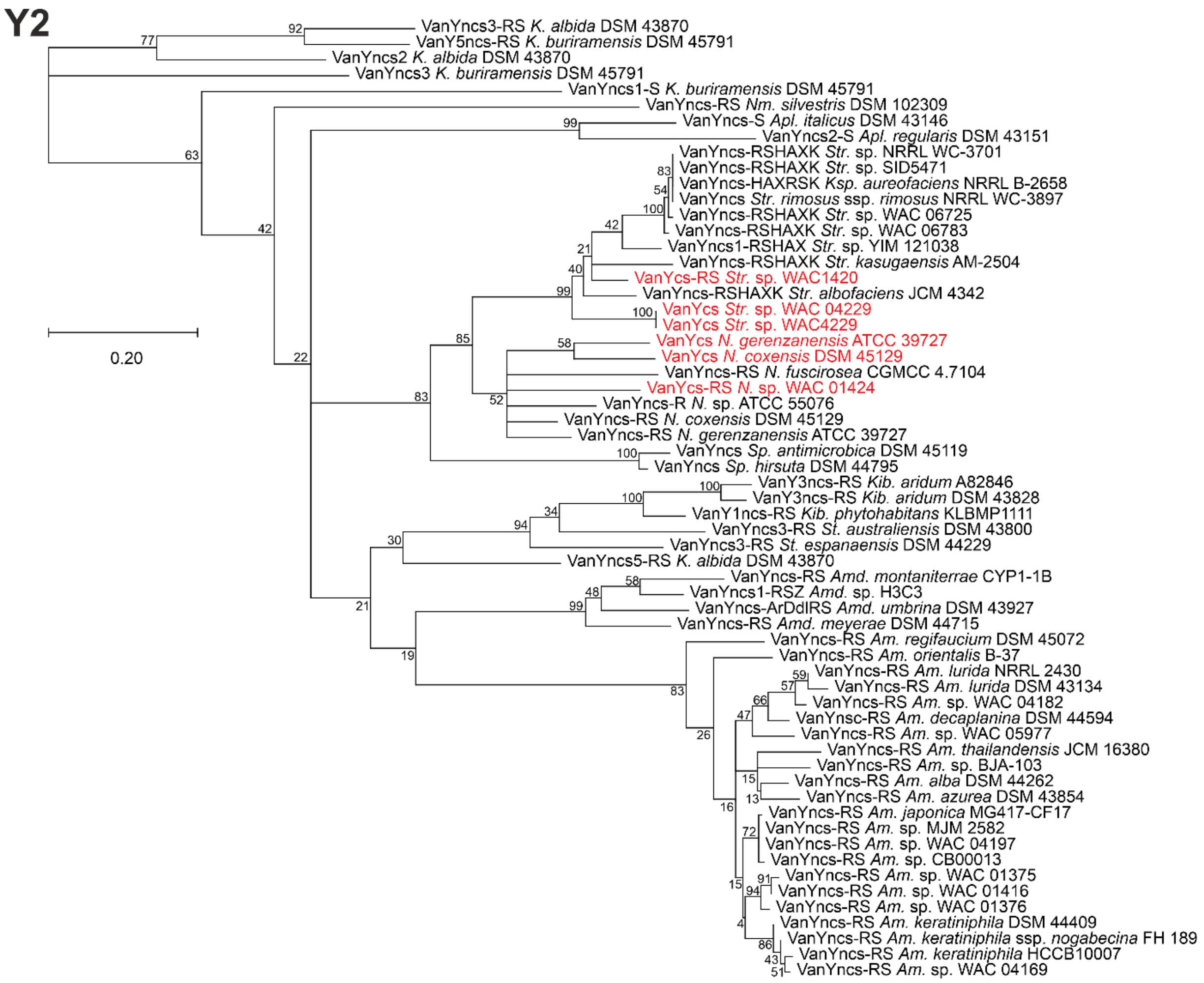

ESM Figure S5. Expanded clade corresponding to the Y2 cluster from ESM Figure S3. BGC-encoded proteins are given in red. Scale bar represents number of substitutions per site.

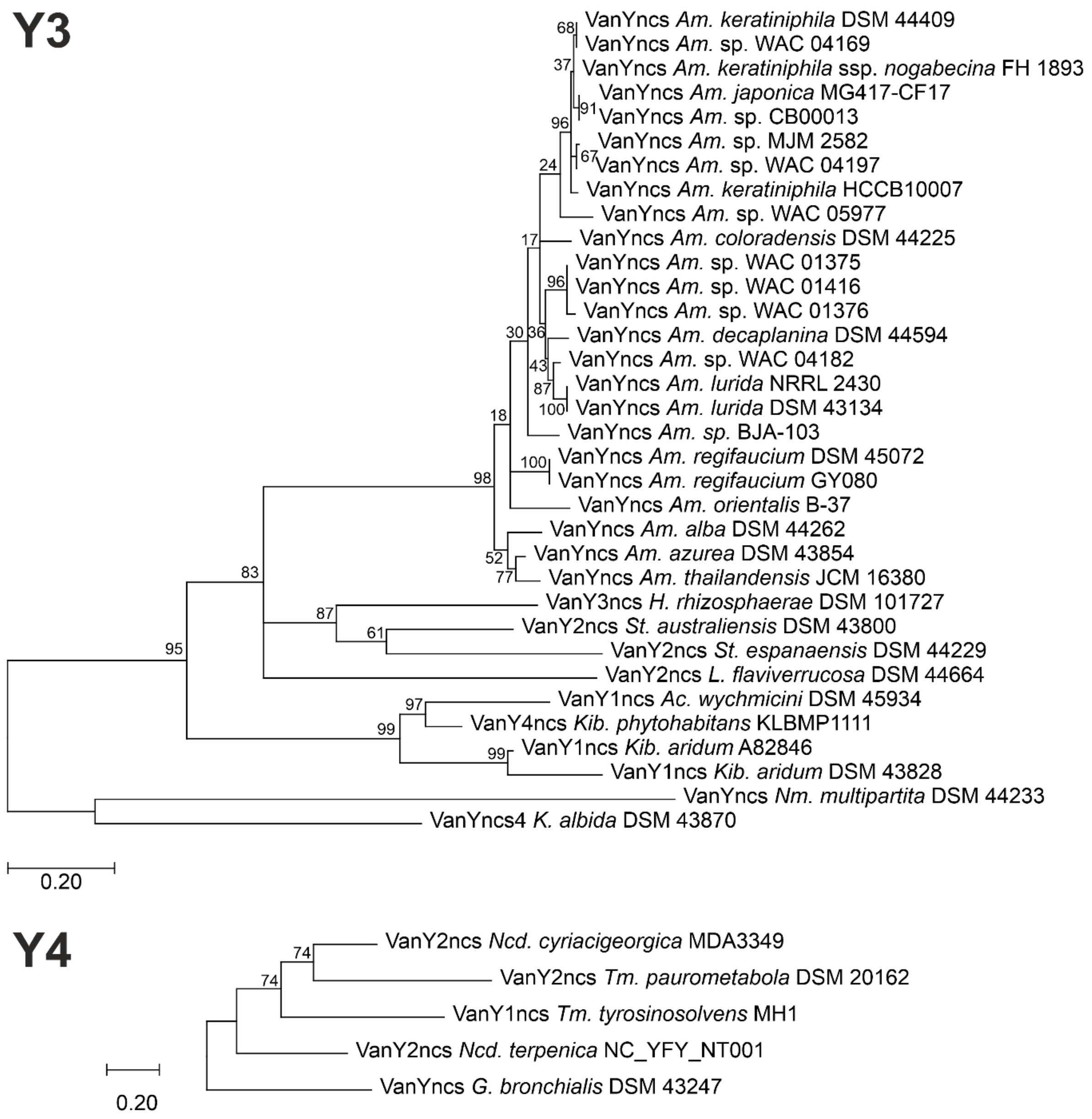

ESM Figure S6. Expanded clades corresponding to the Y3 and Y4 clusters from ESM Figure S3. Scale bar represents number of substitutions per site.

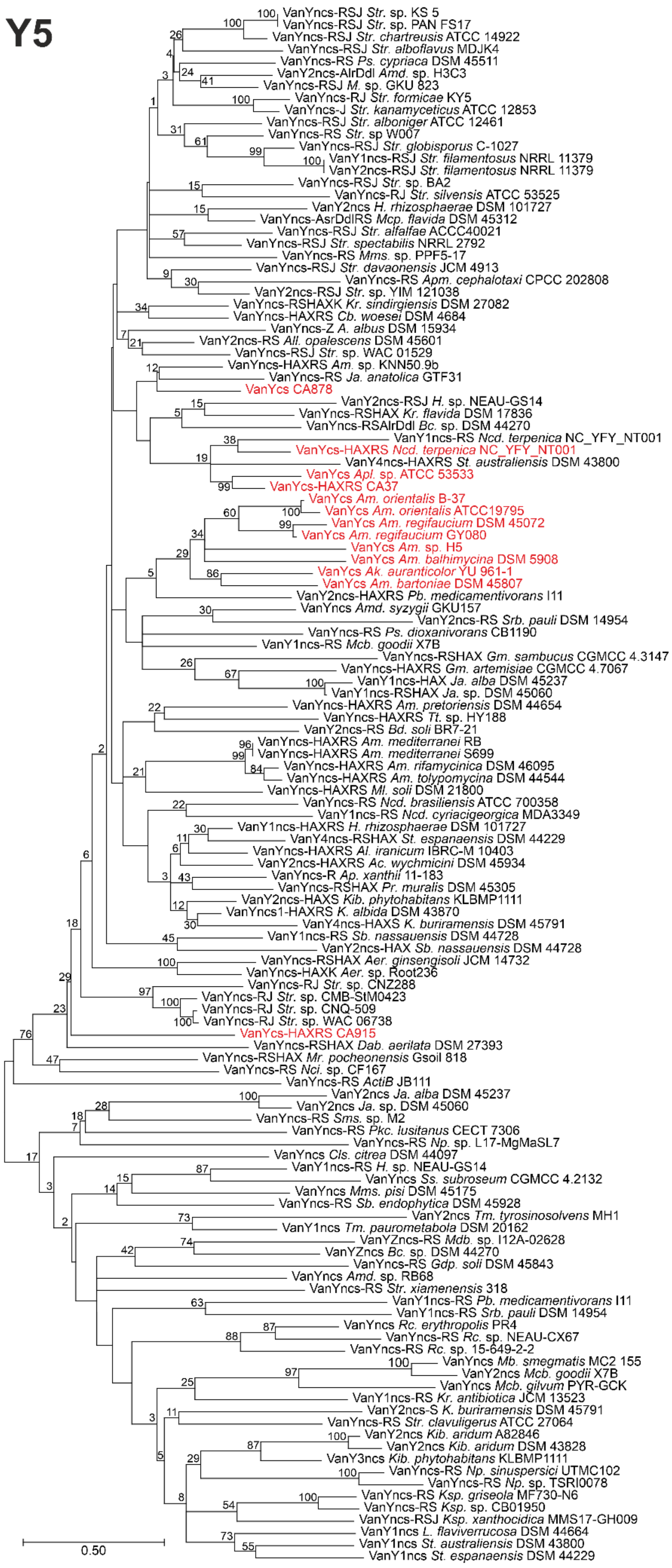

ESM Figure S7. Expanded clade corresponding to the Y5 cluster from ESM Figure S3. BGC-encoded proteins are given in red. Scale bar represents number of substitutions per site.

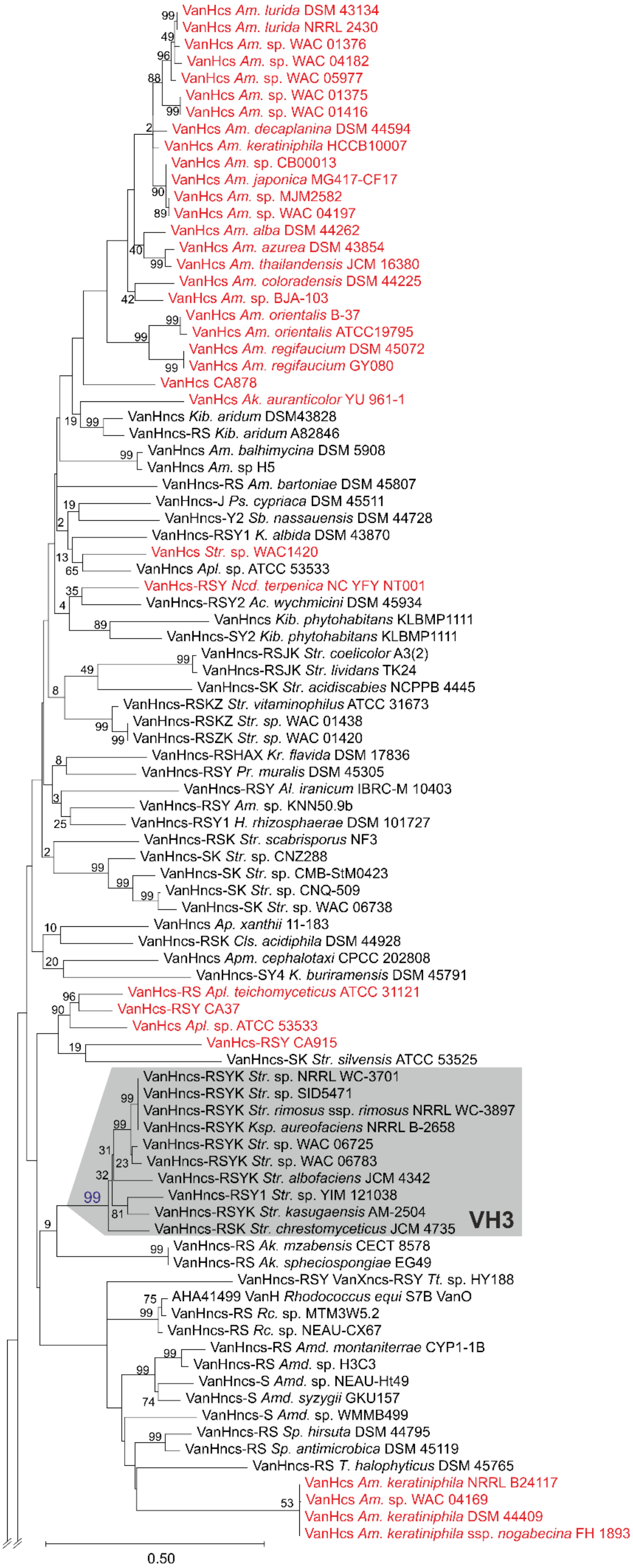

ESM Figure S8. Continued on the next page.

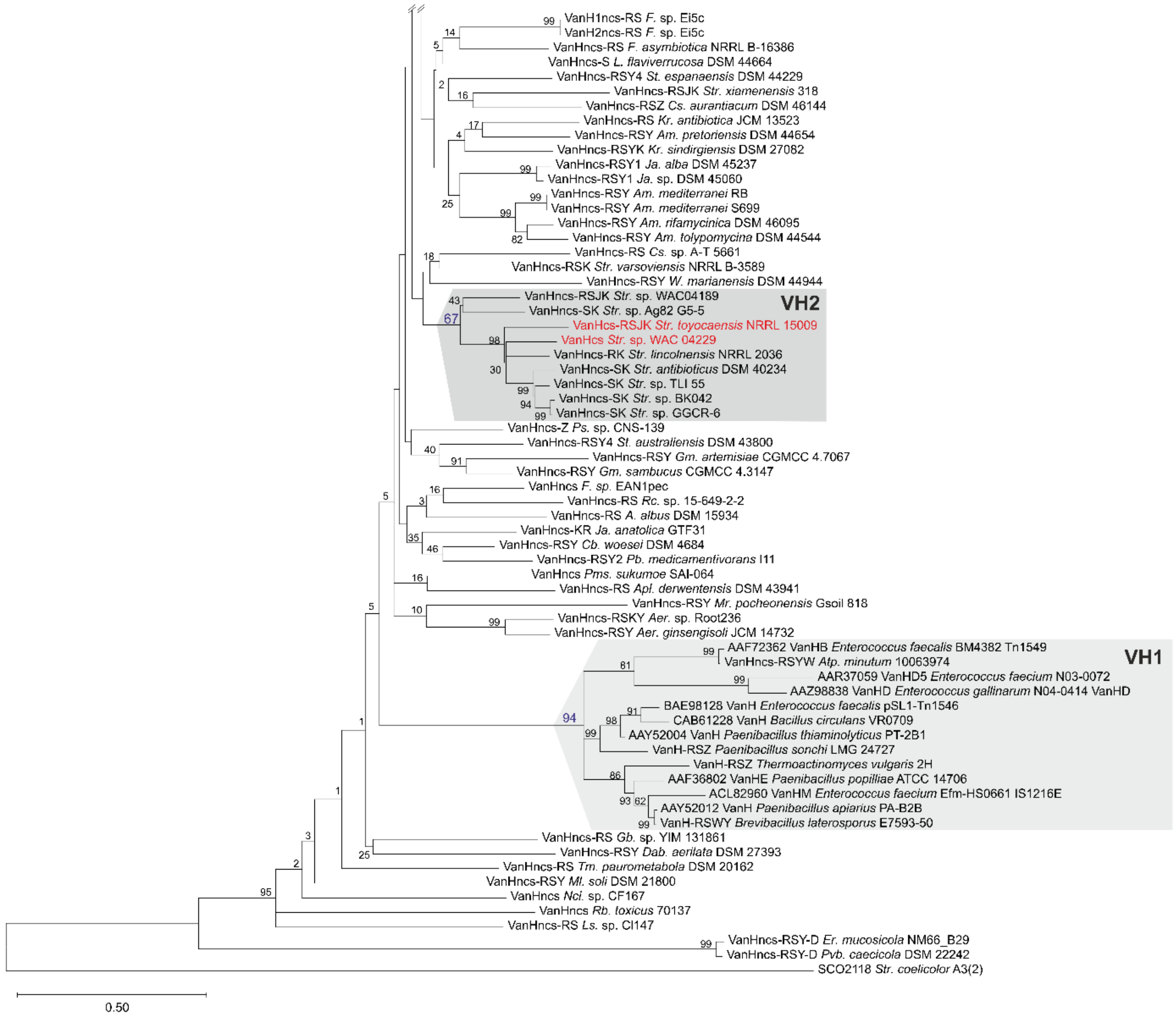

ESM Figure S8. Phylogenetic tree showing the overall phylogeny of VanH-dataset. Three well-supported clusters (VH1-3) are highlighted. Phylogenetic tree was constructed as described in Methods section, SCO2118 – a putative D-lactate dehydrogenase – was used as an outgroup. BGC-encoded proteins are given in red. Scale bar represents number of substitutions per site.

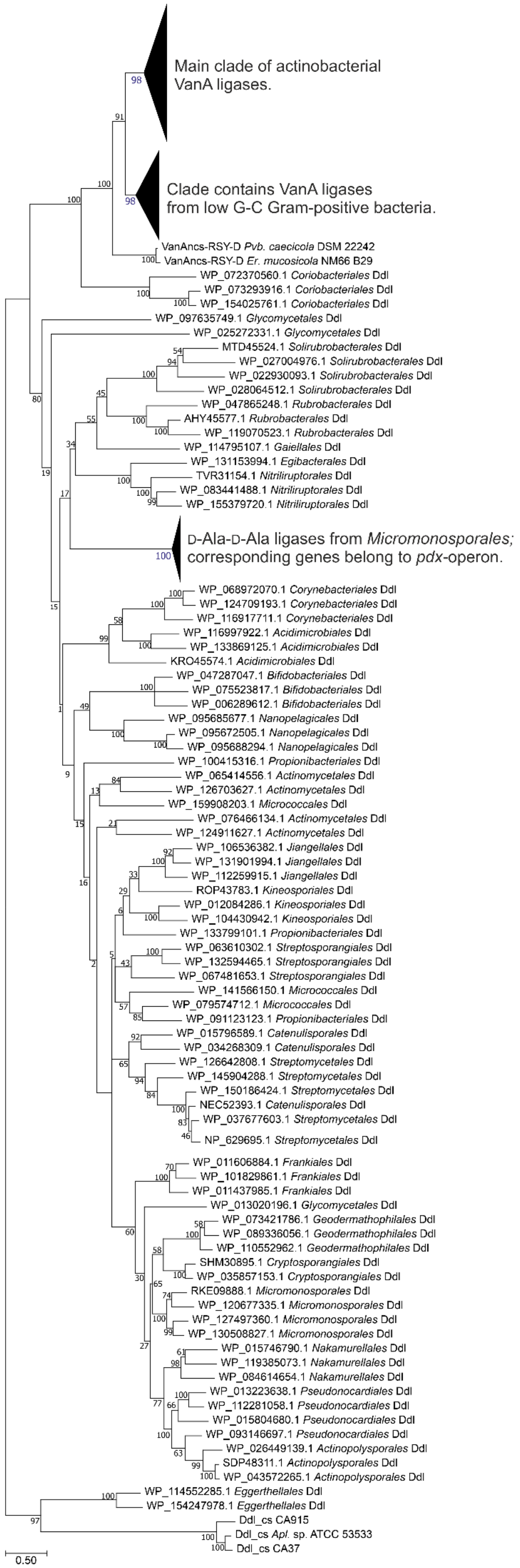

ESM Figure S9. Phylogenetic tree showing the overall phylogeny of the dataset composed with VanA proteins, Ddl-ligases coded in *Micromonosporales* putative *pdx*-operons, *auk*, CA915 and CA37 BGCs as well as actinobacterial "house-keeping" Ddl-ligases. Clades containing Ddl-ligases coded in putative *pdx*-operons and VanA-ligases from low G-C Gram-positive bacteria are collapsed; for these clades expansion please refer to ESM Figures S10 and S11. Expanded crown group of the tree (main clade of actinobacterial VanA-ligases) is given in ESM Figure S12. Phylogenetic tree was constructed as described in Methods section. Scale bar represents number of substitutions per site.

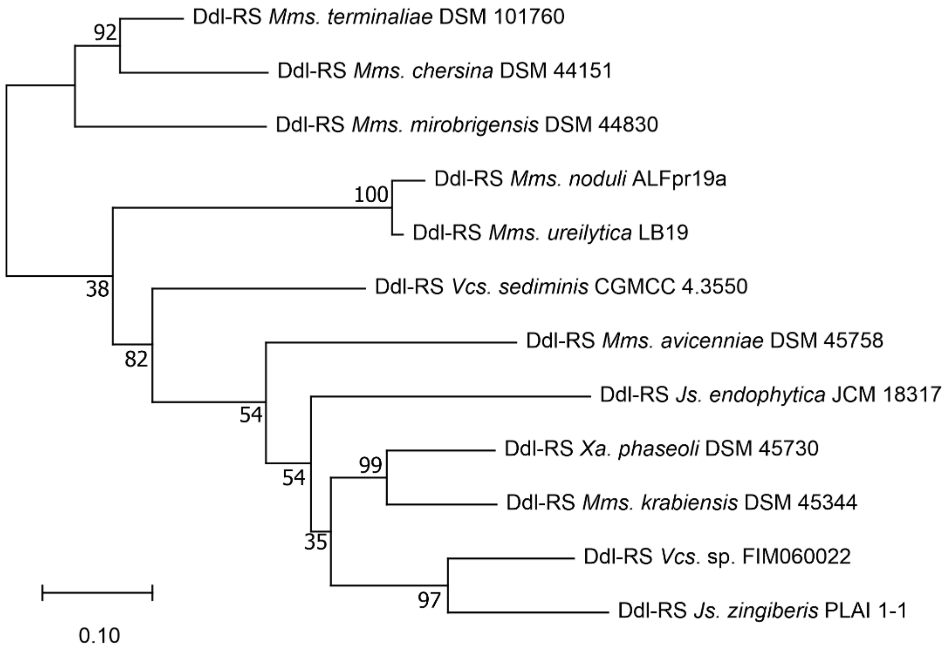

ESM Figure S10. Expanded clade of Ddl-ligases coded in putative *pdx*-operons, which was collapsed on the ESM Figure S9. Scale bar represents number of substitutions per site.

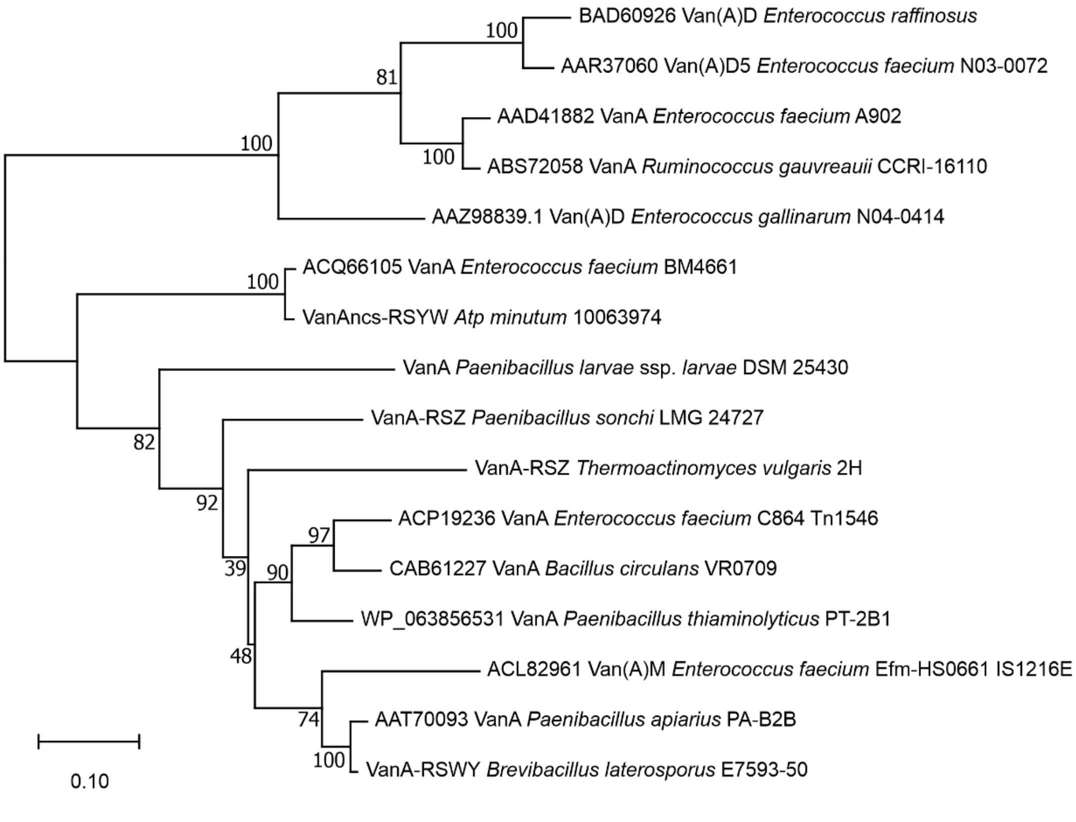

ESM Figure S11. Expanded clade containing VanA-ligases from low G-C Gram-positive bacteria, which was collapsed on the ESM Figure S9. Scale bar represents number of substitutions per site.

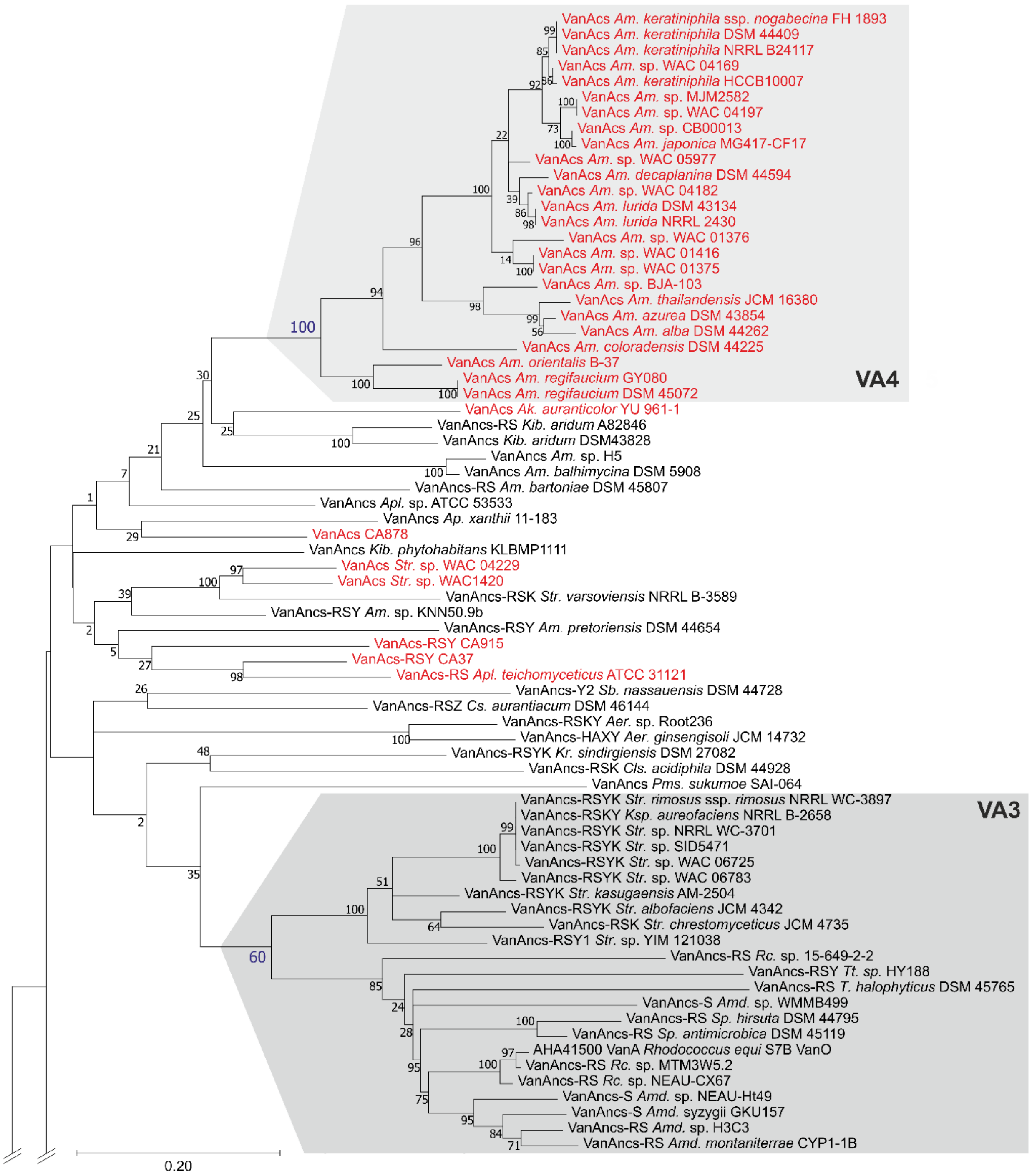

ESM Figure S12. Continued on the next page.

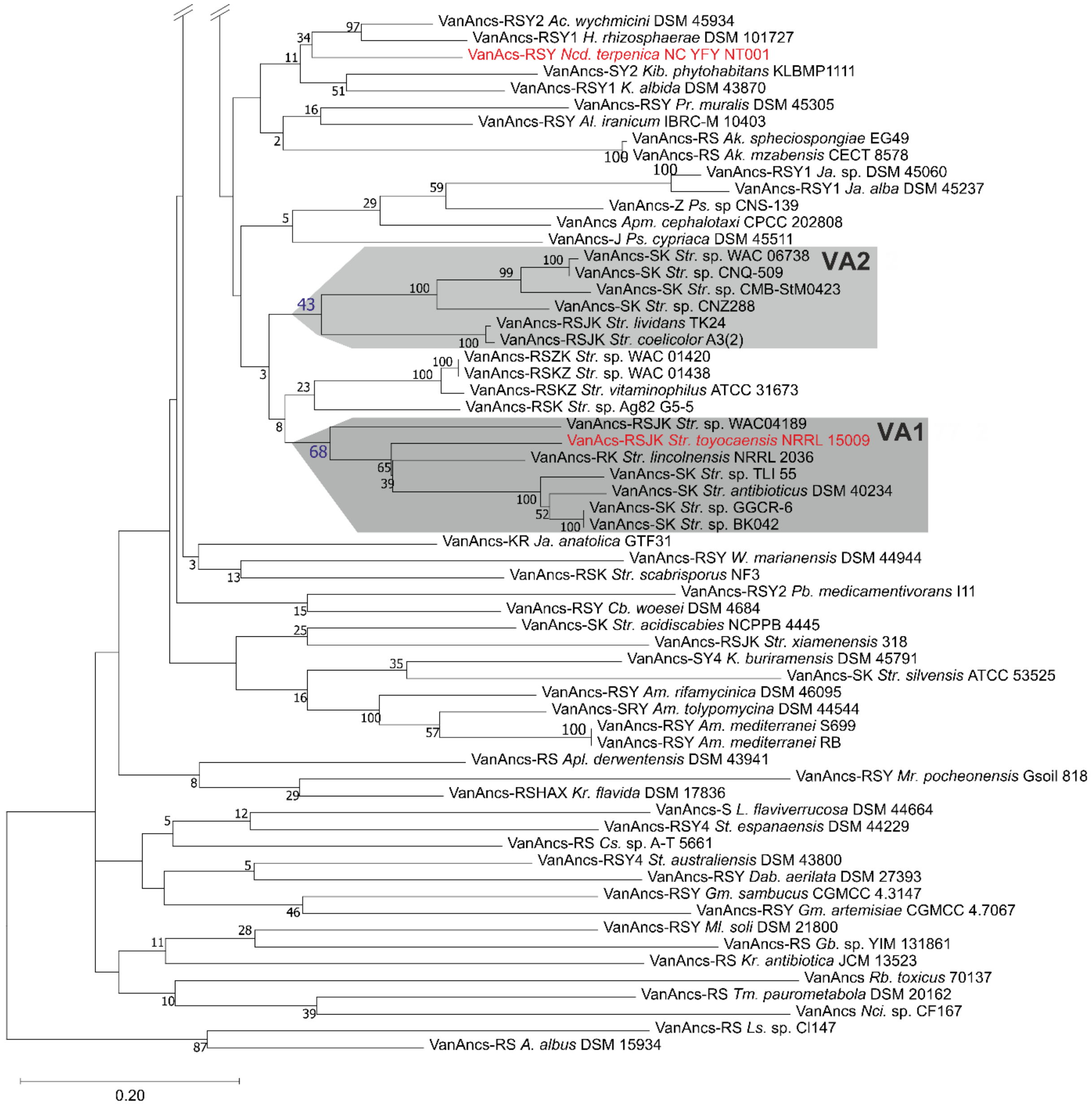

ESM Figure S12. Expanded crown group of VanA-phylogenetic tree, which was collapsed on the ESM Figure S9. Four clusters with the best bootstrap support were distinguished – VA1-4. BGC-encoded proteins are given in red. Please see main text for more details. Scale bar represents number of substitutions per site.

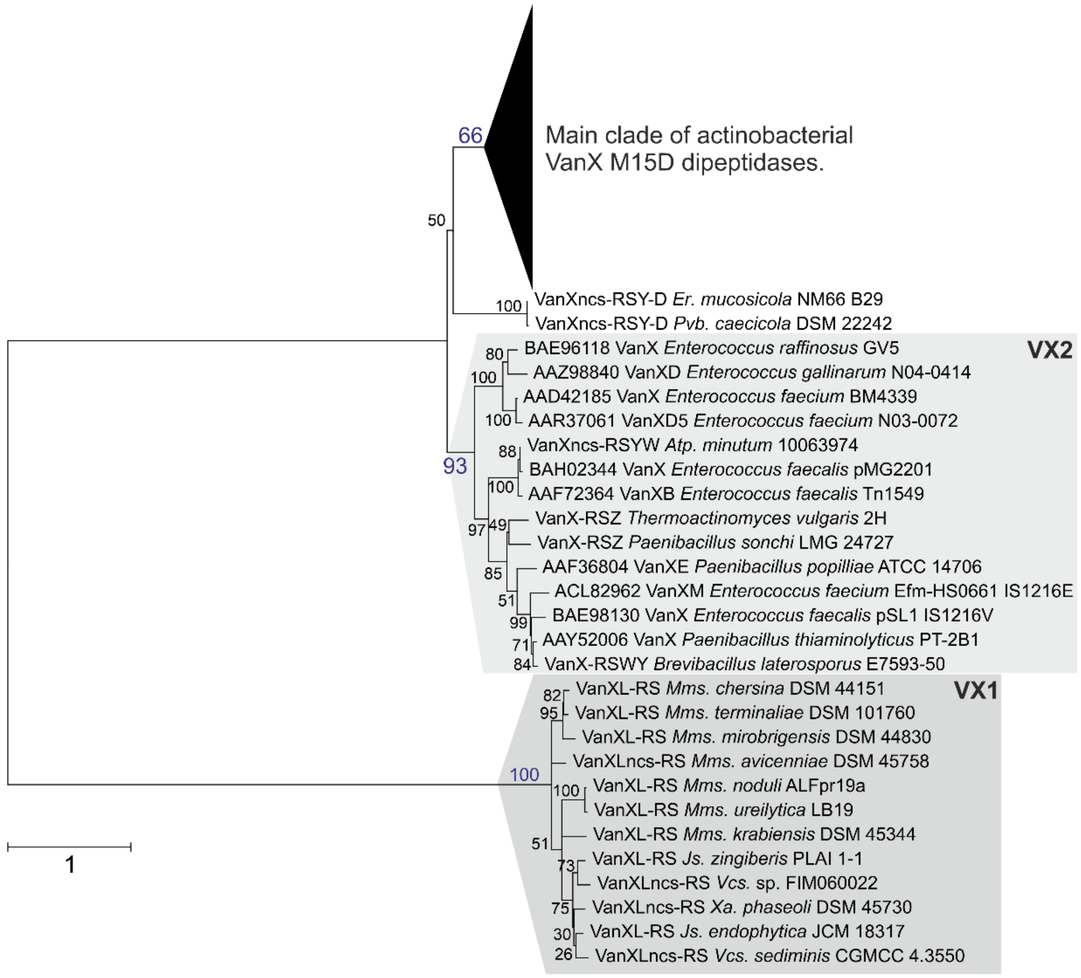

ESM Figure S13. Phylogenetic tree showing the overall phylogeny of the dataset composed with VanX-like proteins, including the ones coded in *Micromonosporales* putative *pdx*-operons. Tree contains well-separated clusters containing VanX-like proteins coded in putative *pdx*-operons (VX1) and VanX proteins from low G-C Gram-positive bacteria (VX2). The crown group of the tree (main clade of actinobacterial VanX M15D dipeptidases) is collapsed; for the expanded version please see ESM Figure S14. Phylogenetic tree was constructed as described in Methods section. Scale bar represents number of substitutions per site.

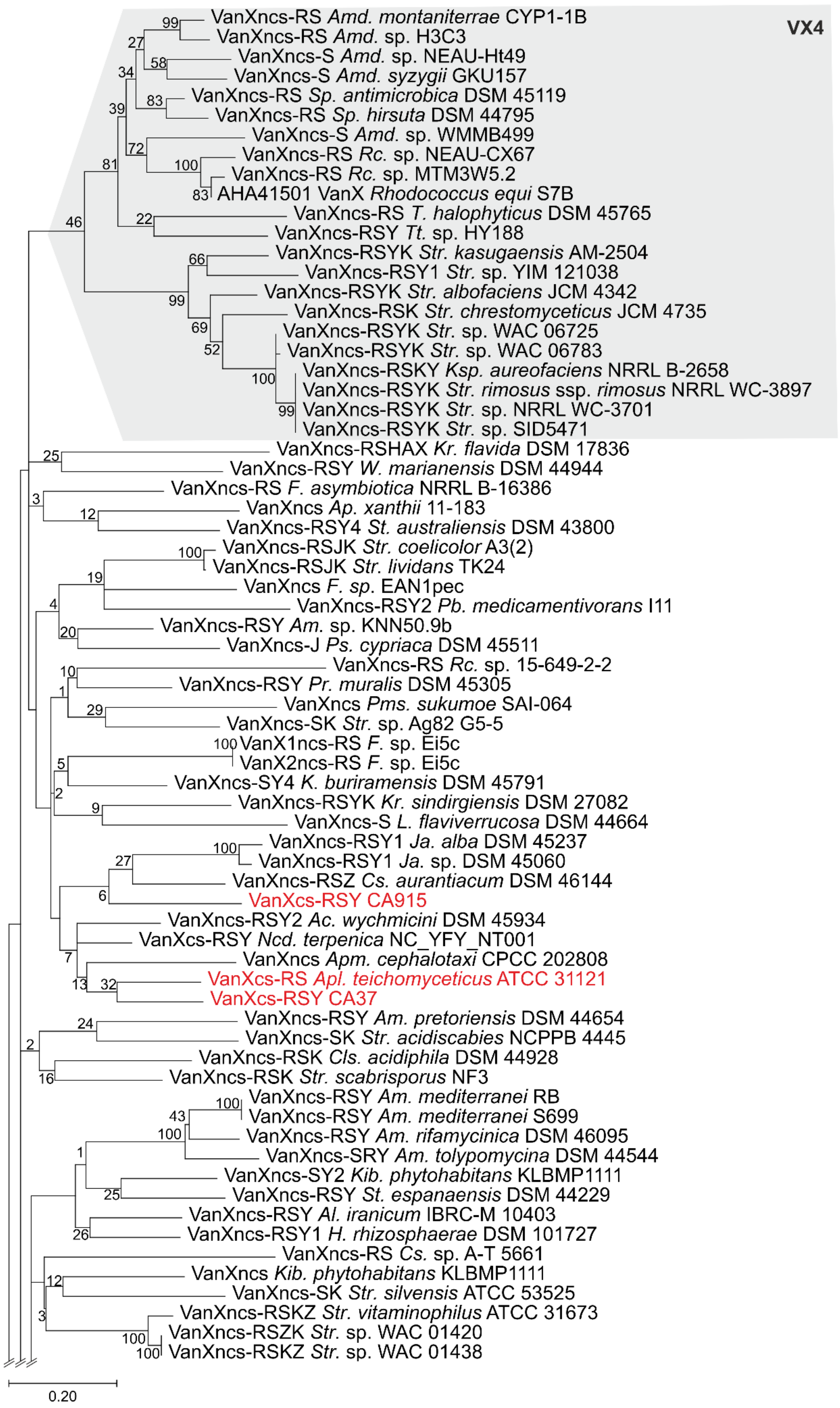

ESM Figure S14. Continued on the next page.

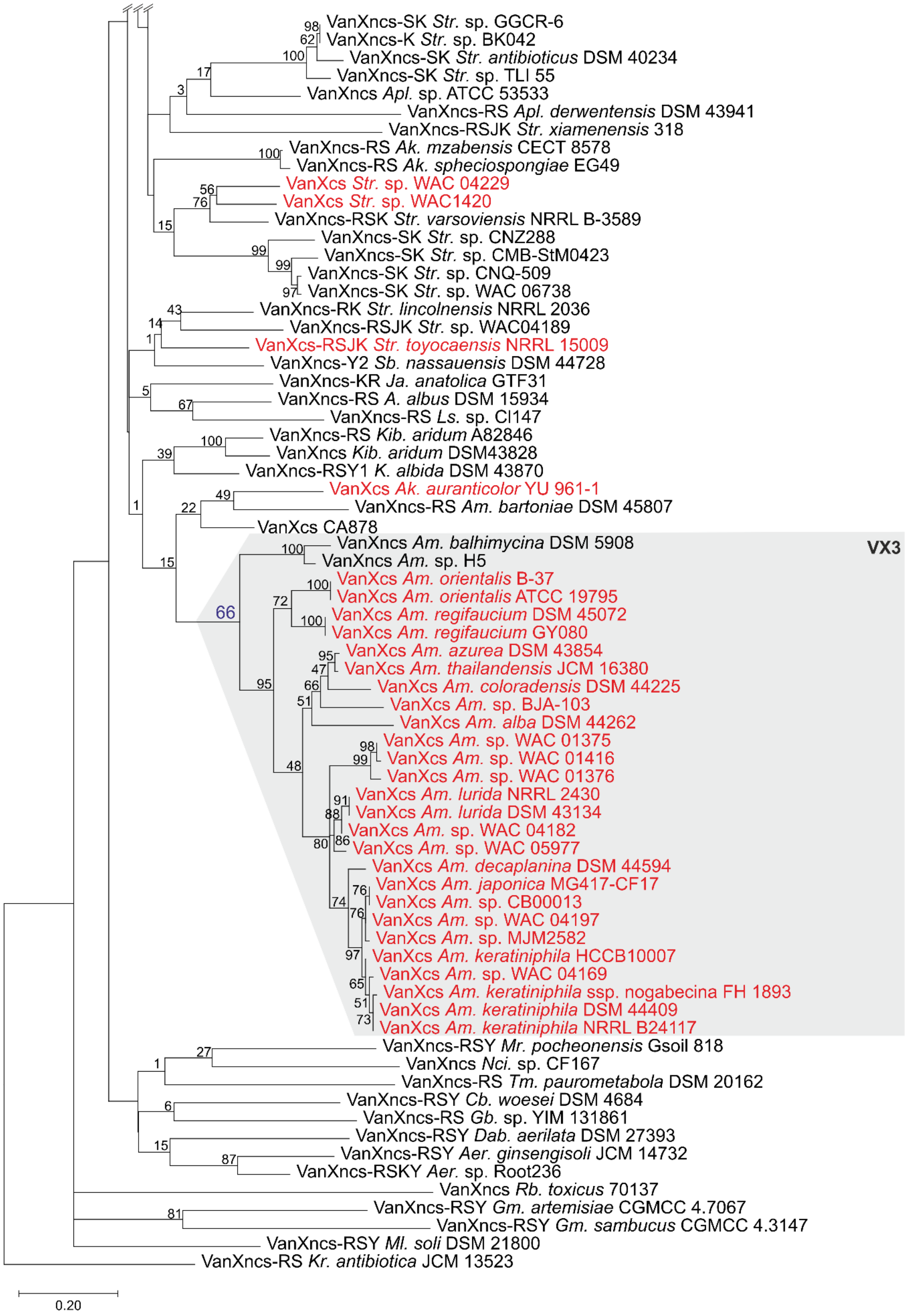

ESM Figure S14. Expanded version of the crown group of actinobacterial VanX M15D dipeptidases from ESM Figure S13. Please refer to the main text for more details. BGC-encoded proteins are given in red. Scale bar represents number of substitutions per site.

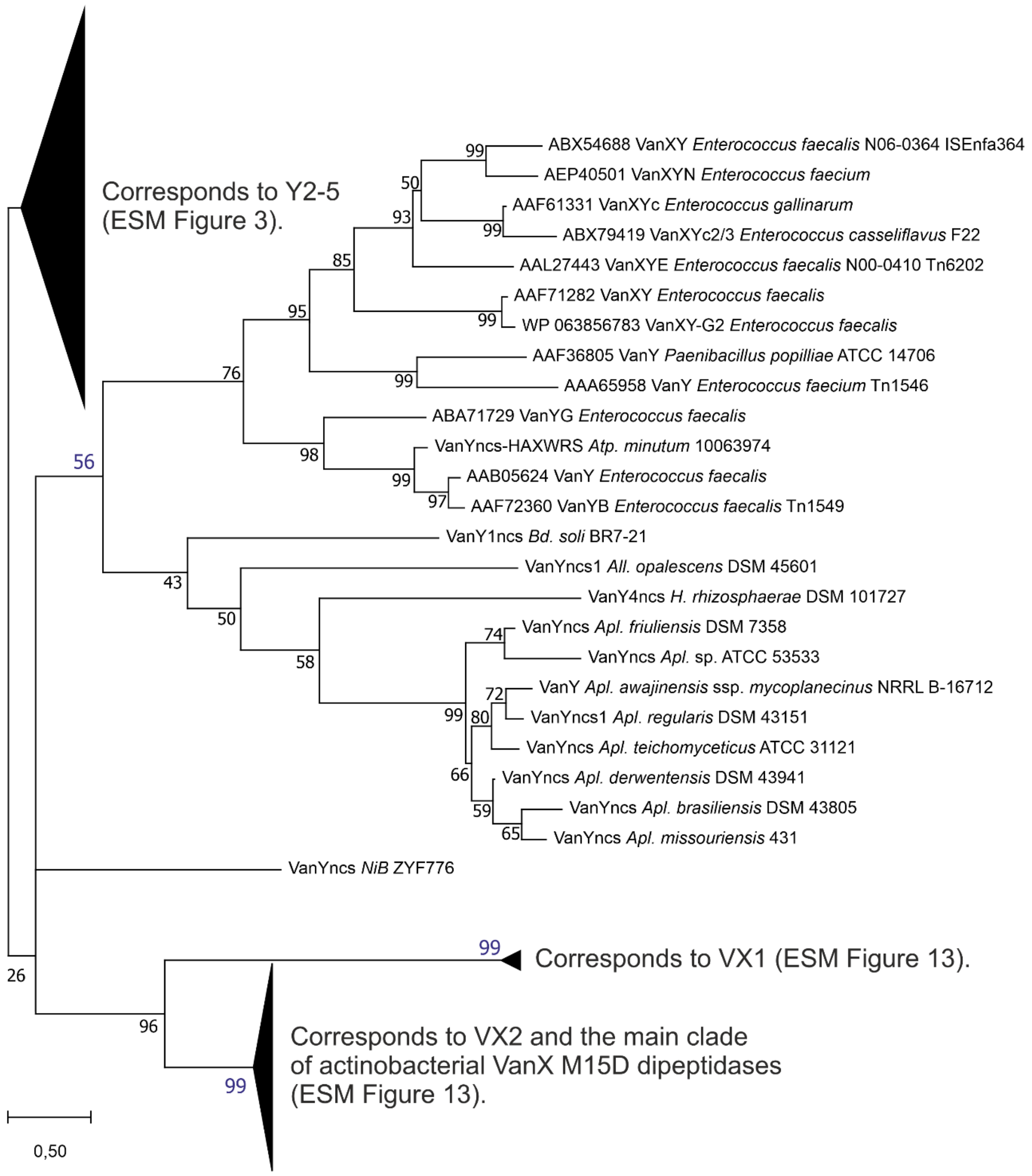

ESM Figure S15. Phylogeny of combined datasets for VanY-like M15B carboxypeptidases and VanX M15D dipeptidases, including also VanXY-proteins. Phylogenetic tree was constructed as described in Methods section. Scale bar represents number of substitutions per site.

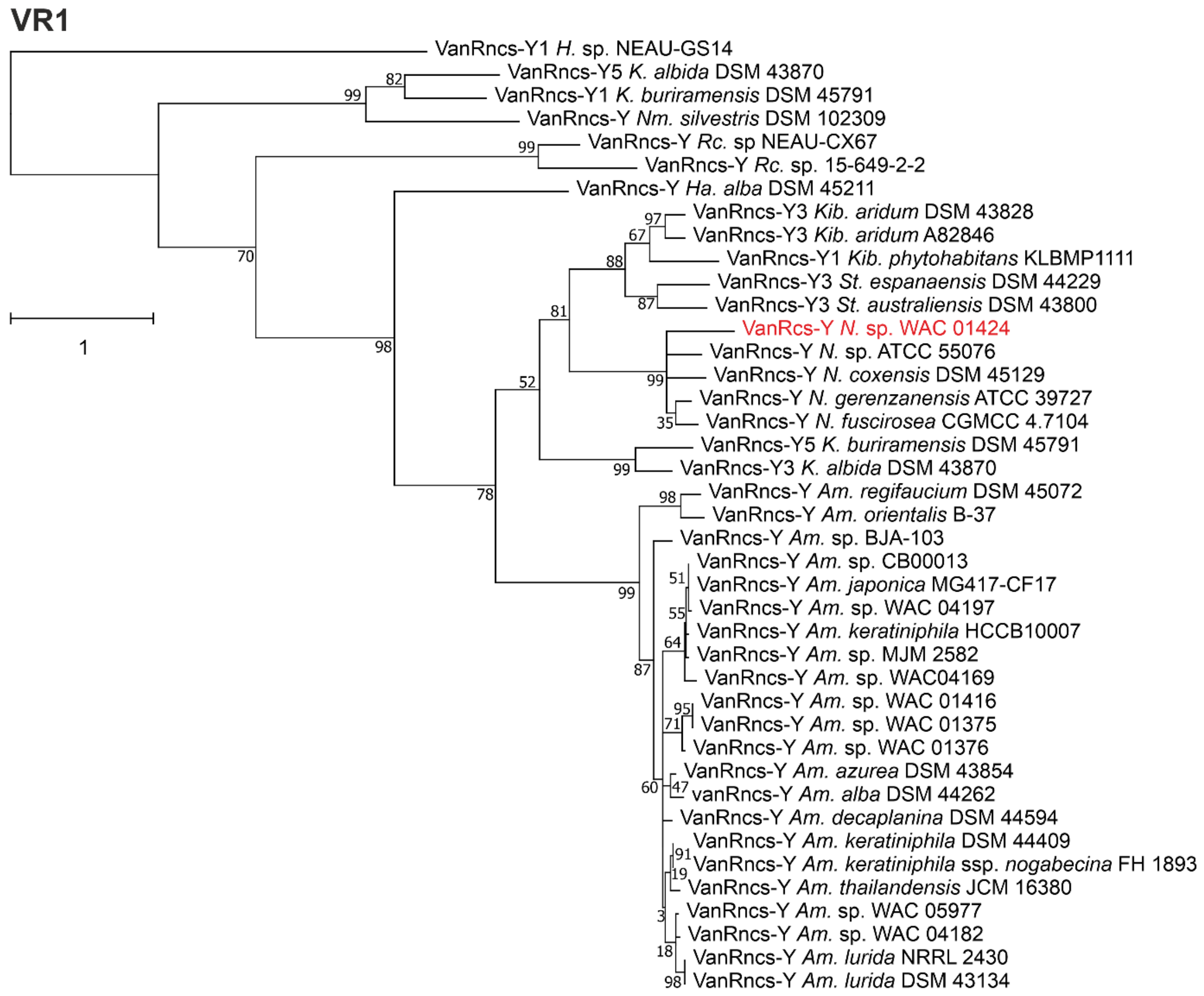

ESM Figure S16. Expanded version of the VR1 cluster from the phylogenetic tree of actinobacterial VanR-like response regulators (Figure 11a). Please refer to the main text for more details. BGC-encoded proteins are given in red. Scale bar represents number of substitutions per site.

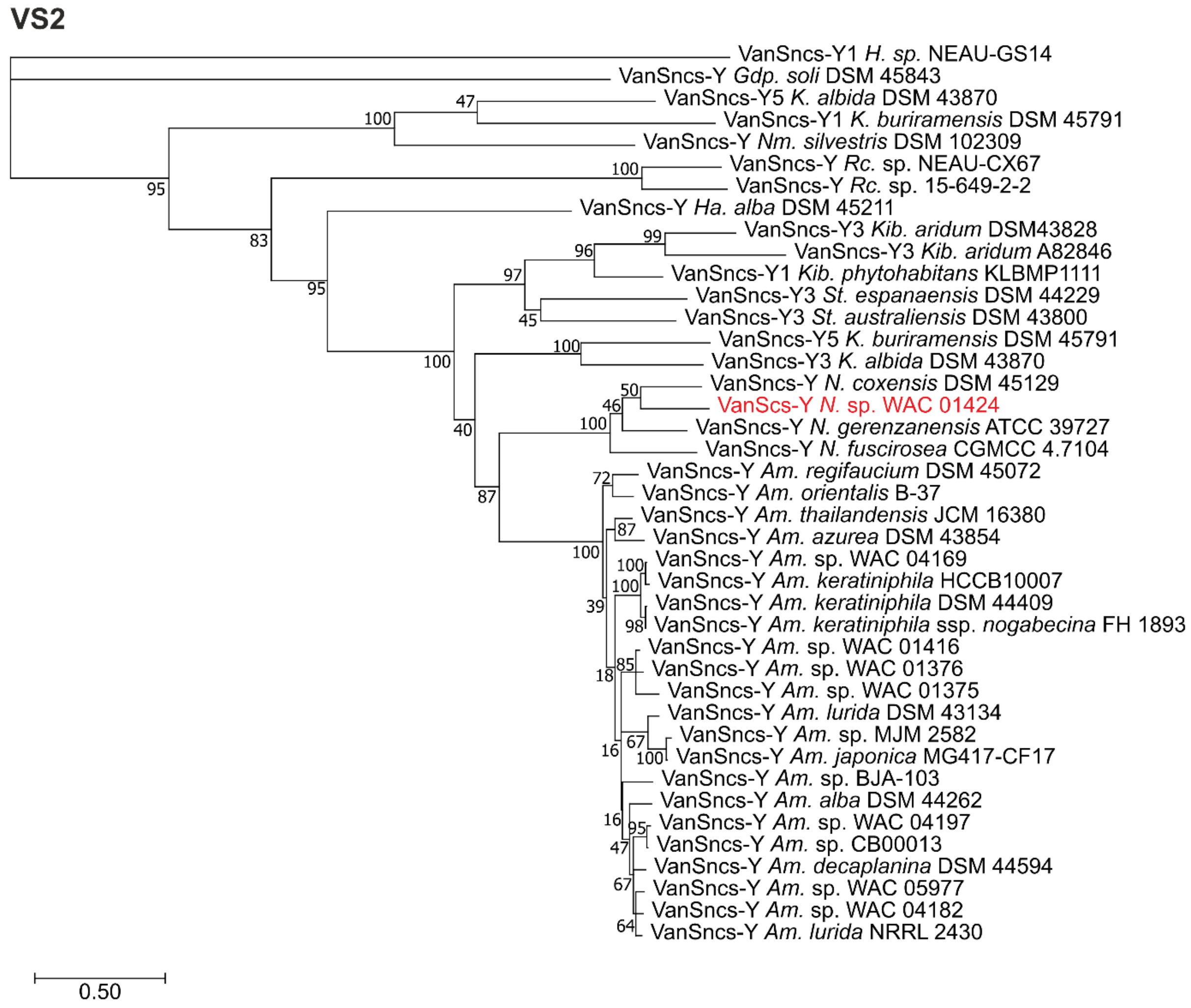

ESM Figure S17. Expanded version of the VS2 cluster from the phylogenetic tree of actinobacterial VanS-like sensor histidine kinases (Figure 11b). Please refer to the main text for more details. BGC-encoded proteins are given in red. Scale bar represents number of substitutions per site.

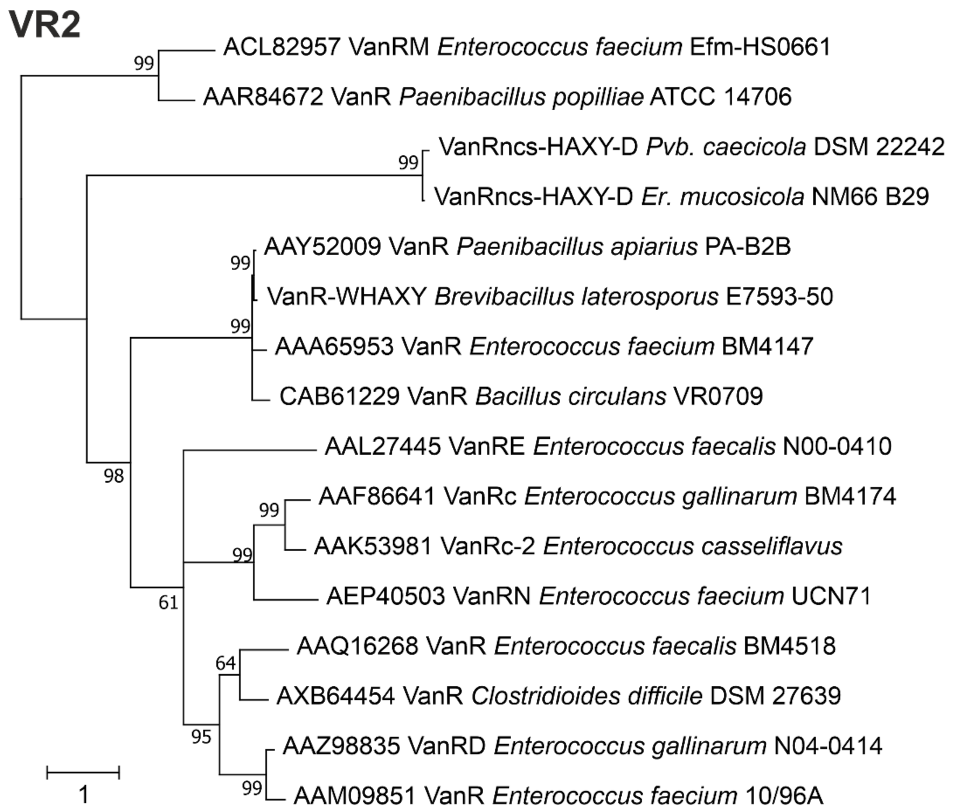

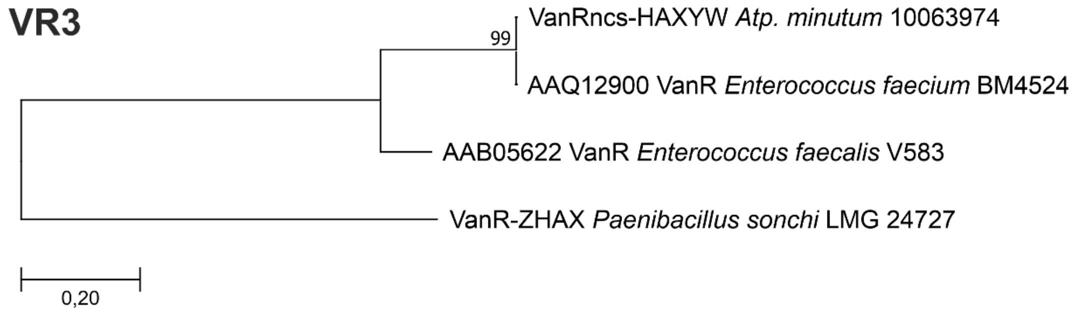

ESM Figure S18. Expanded versions of the VR2 and VR3 clusters from the phylogenetic tree of actinobacterial VanR-like response regulators (Figure 11a). Please refer to the main text for more details. Scale bar represents number of substitutions per site.

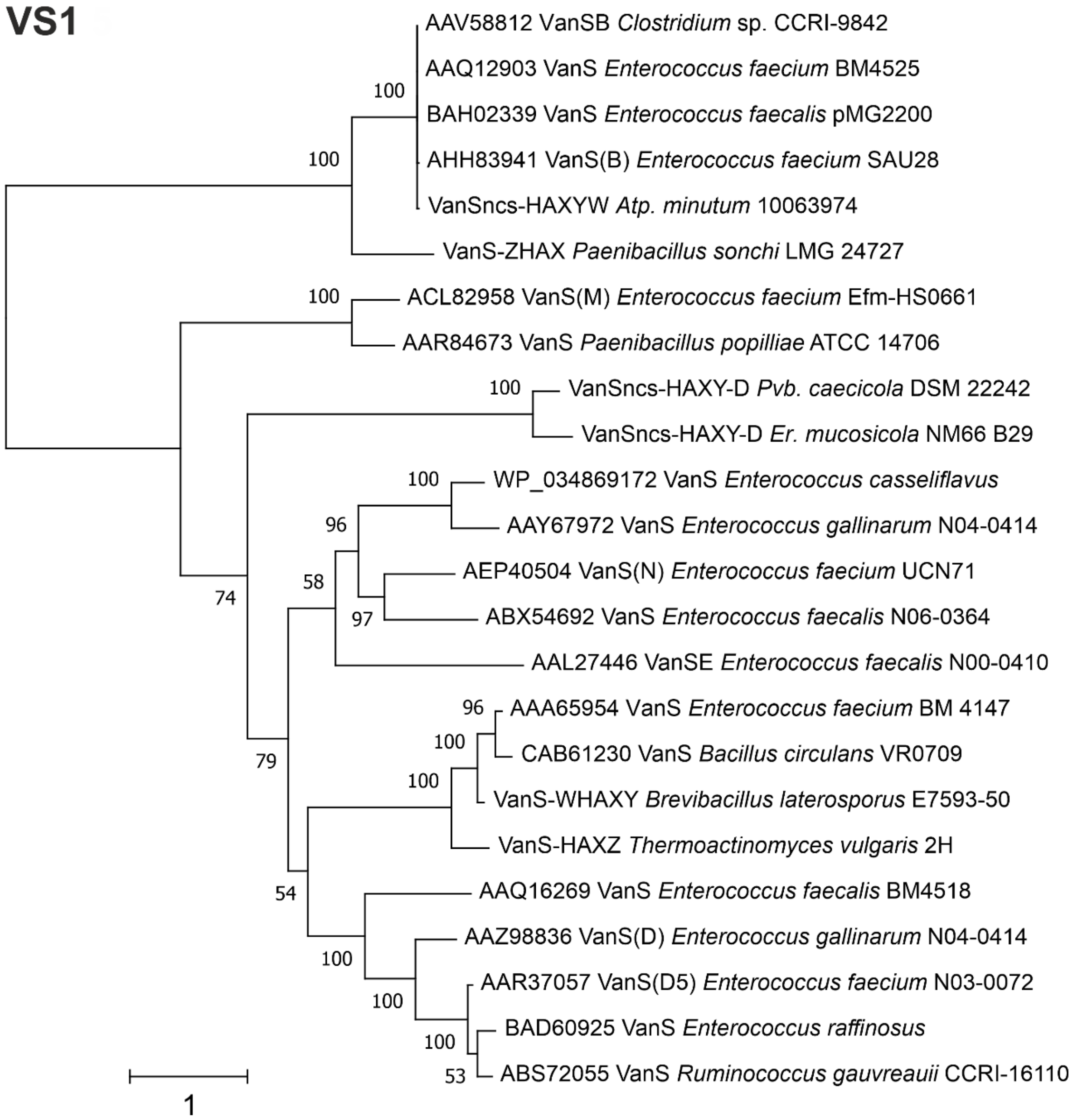

ESM Figure S19. Expanded version of the VS1 cluster from the phylogenetic tree of actinobacterial VanS-like sensor histidine kinases (Figure 11b). Please refer to the main text for more details. Scale bar represents number of substitutions per site.

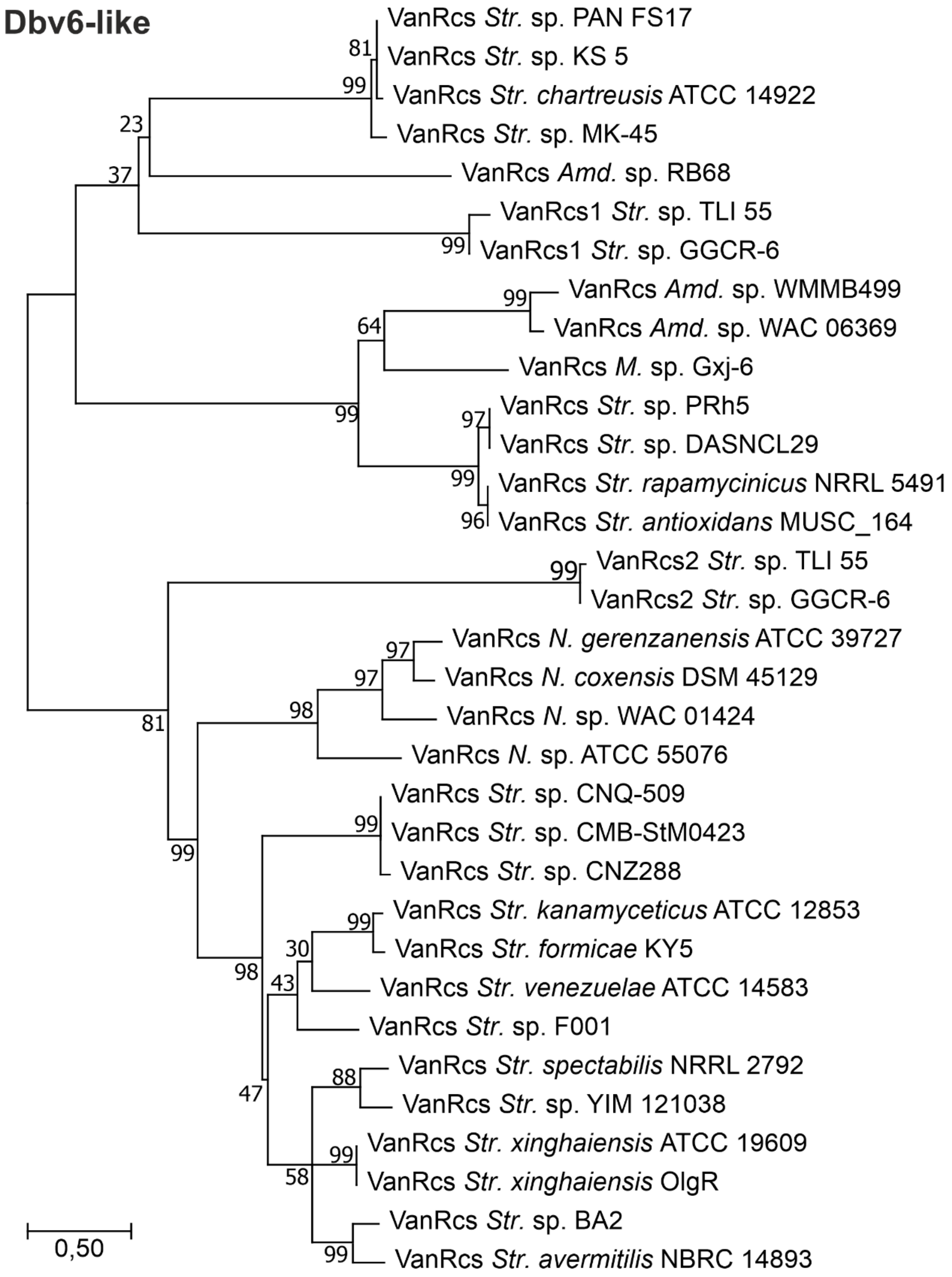

ESM Figure S20. Expanded version of the Dbv6-like cluster from the phylogenetic tree of actinobacterial VanR-like response regulators, coded within type V GPA BGCs, *Nonomuraea* spp. type IV GPA BGCs, and *feg*-like BGCs (Figure 11a). Please refer to the main text for more details. Scale bar represents number of substitutions per site.

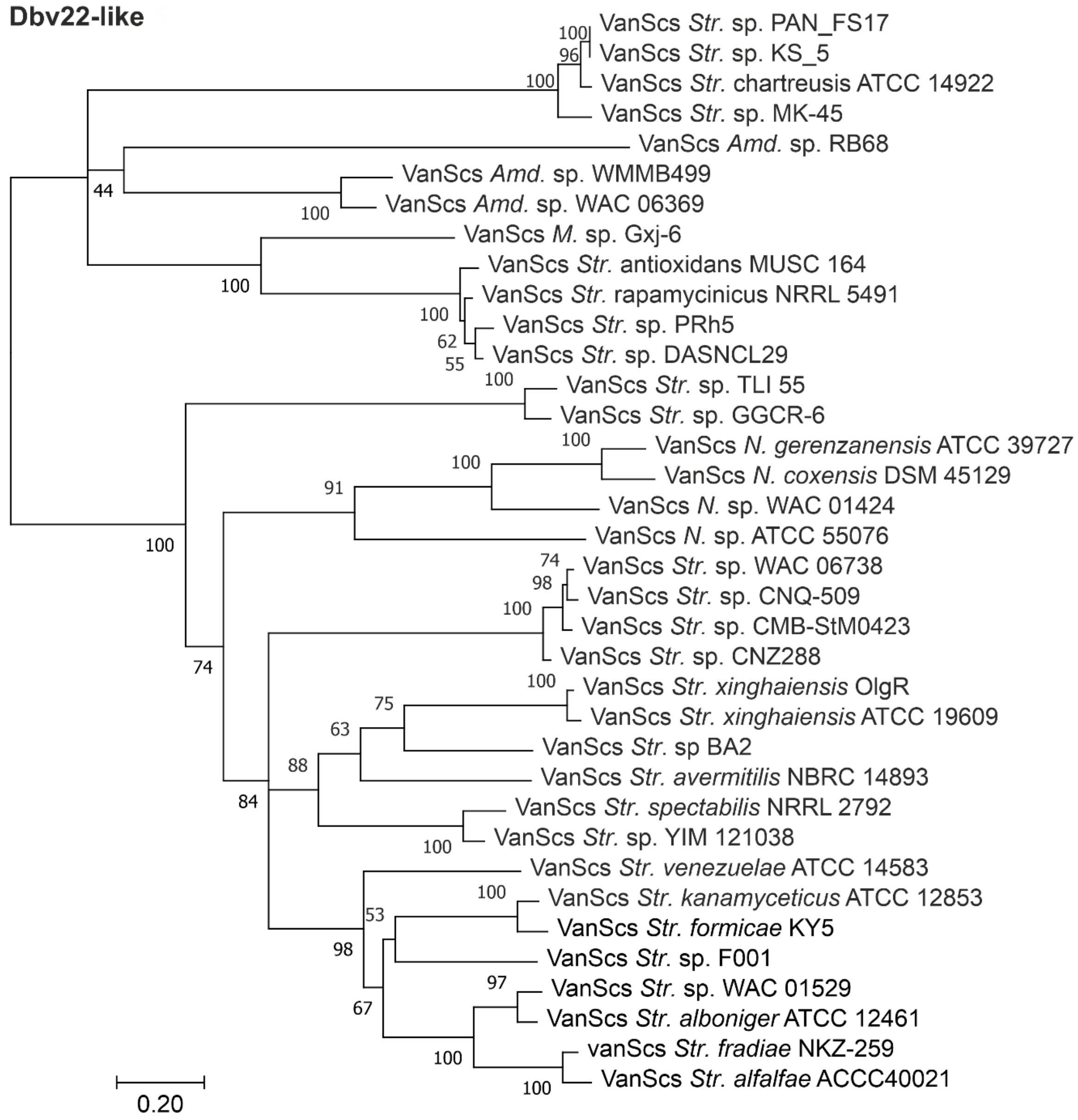

ESM Figure S21. Expanded version of the Dbv22-like cluster from the phylogenetic tree of actinobacterial VanS-like sensor histidine kinases, coded within type V GPA BGCs, *Nonomuraea* spp. type IV GPA BGCs, and *feg*-like BGCs (Figure 11b). Please refer to the main text for more details. Scale bar represents number of substitutions per site.

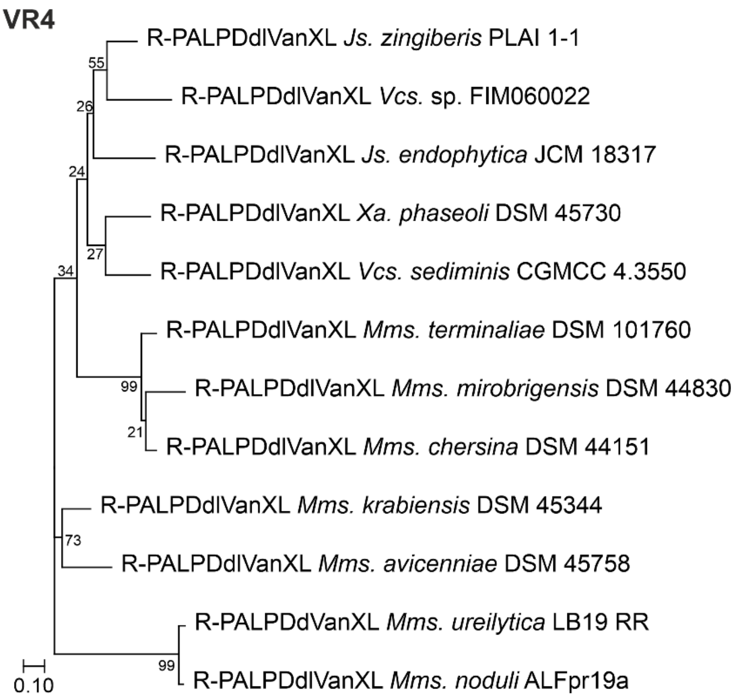

ESM Figure S22. Expanded version of the VR4 cluster from the phylogenetic tree of actinobacterial VanR-like response regulators, coded within putative *pdx*-operons from *Micromonosporales* (Figure 11a). Please refer to the main text for more details. Scale bar represents number of substitutions per site.

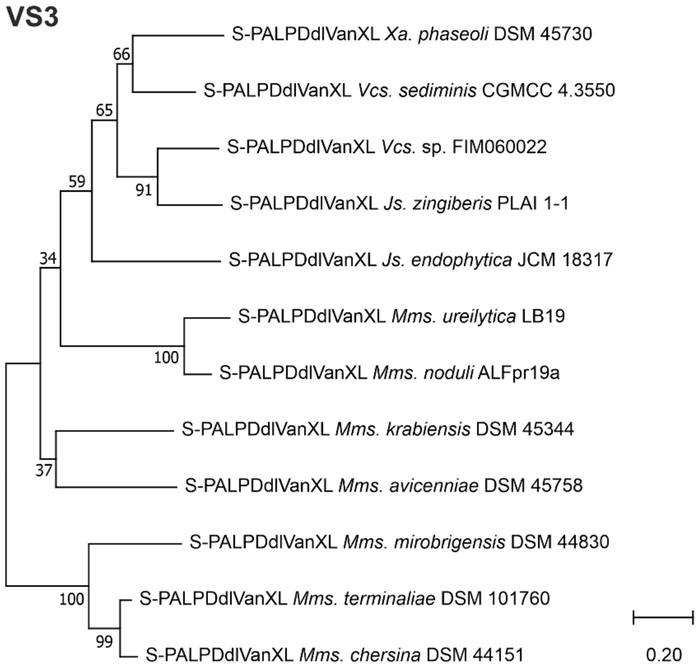

ESM Figure S23. Expanded version of the VS3 cluster from the phylogenetic tree of actinobacterial VanS-like sensor histidine kinases, coded within putative *pdx*-operons from *Micromonosporales* (Figure 11b). Please refer to the main text for more details. Scale bar represents number of substitutions per site.

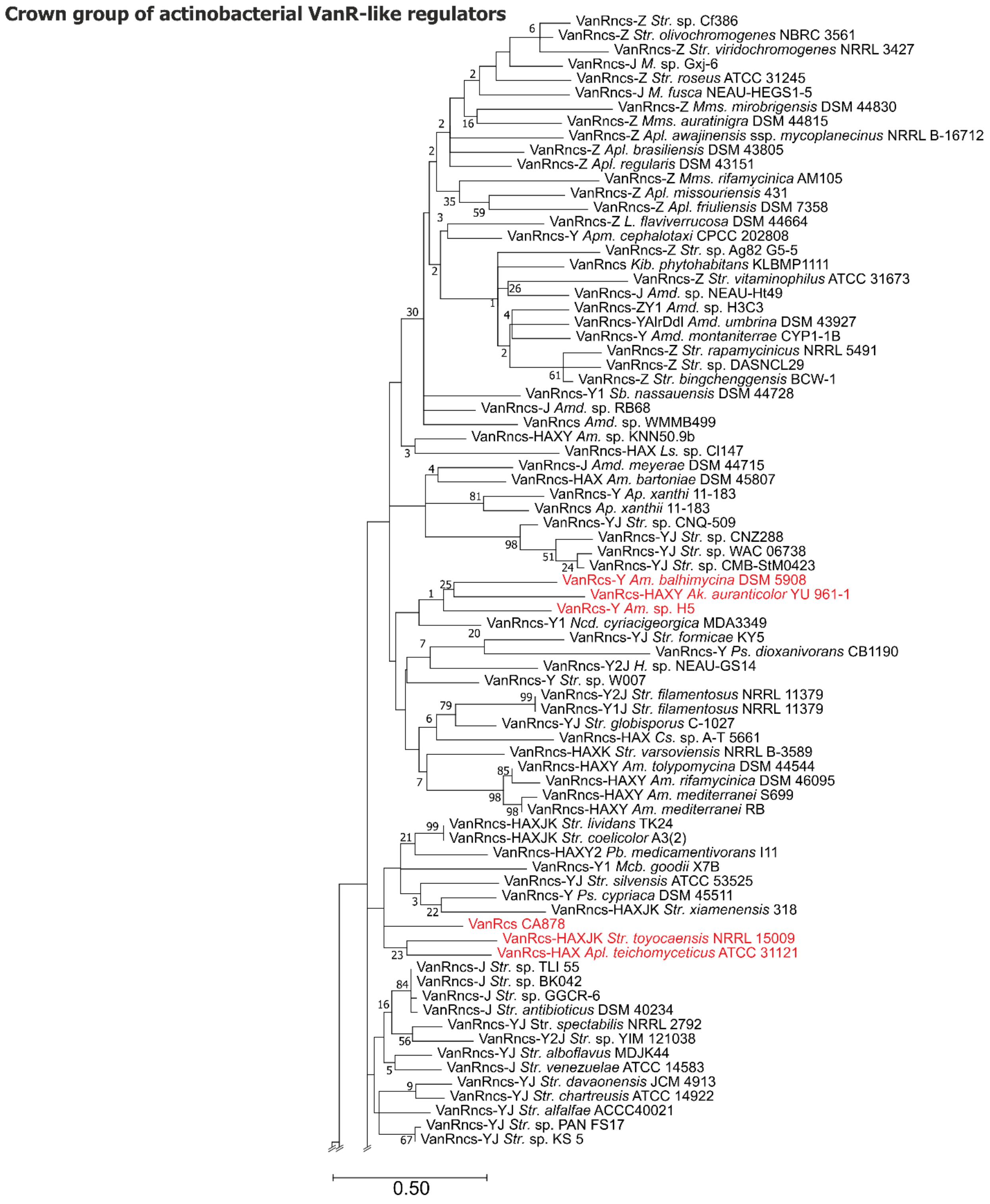

ESM Figure S24. Continued on the next page.

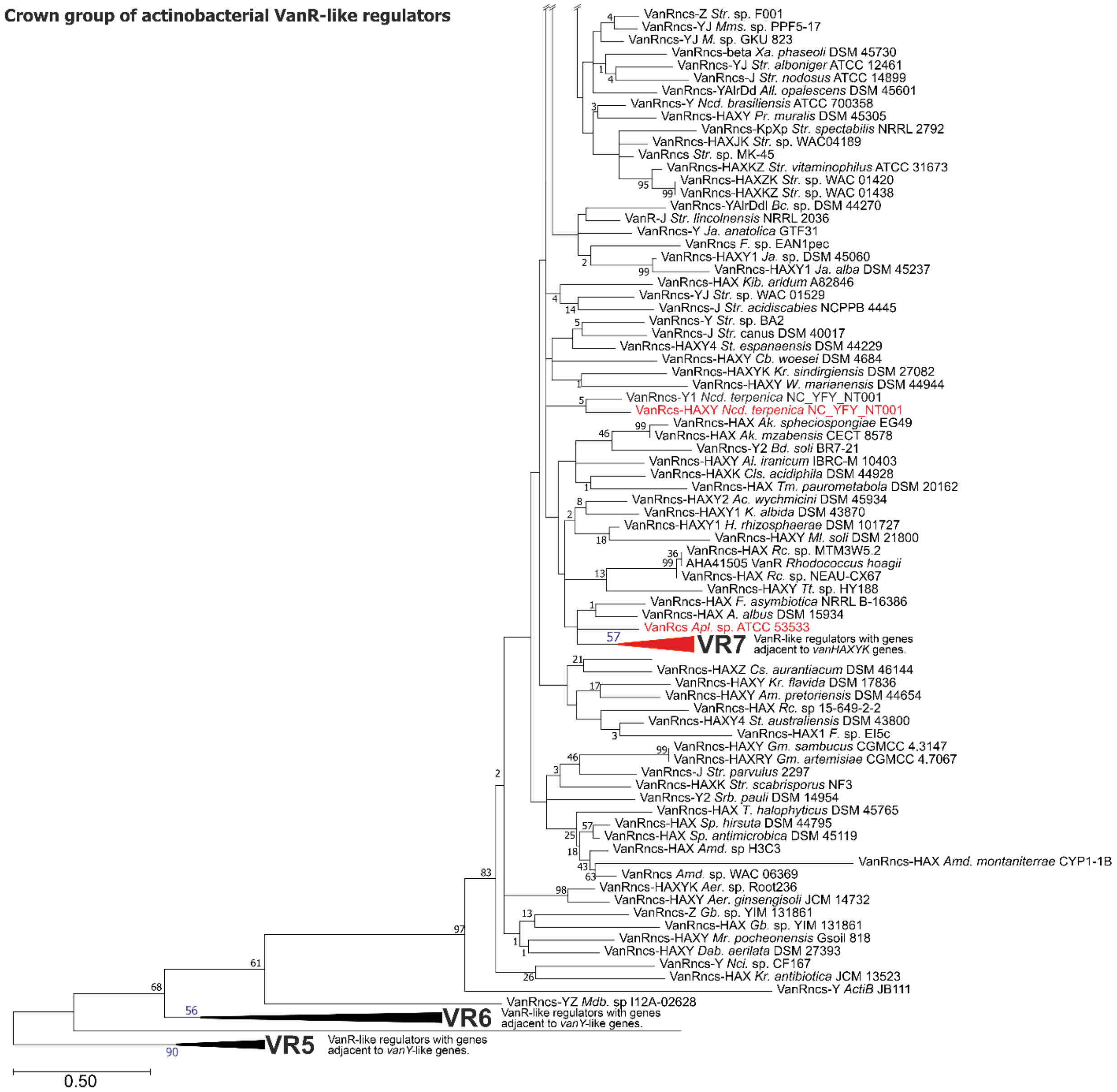

ESM Figure S24. Expanded version of the clade corresponding to the crown group of bacterial VanR response regulators from tree on Figure 11a. Well-defined clusters VR5-7 were collapsed; their expanded versions are given on ESM Figure S26. Please refer to the main text for more details. BGC-encoded proteins are given in red. Scale bar represents number of substitutions per site.

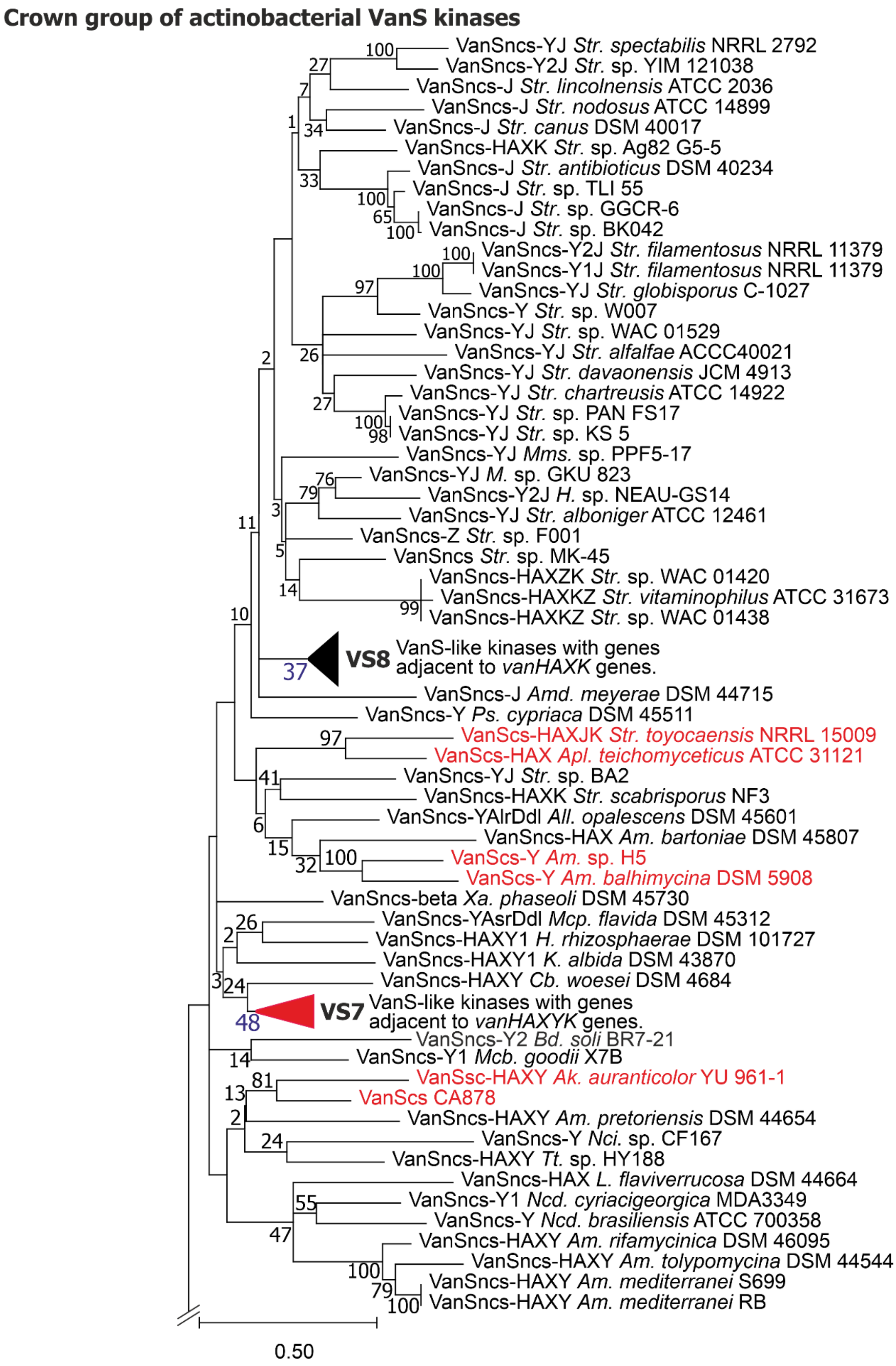

ESM Figure S25. Continued on the next page.

Crown group of actinobacterial VanS kinases

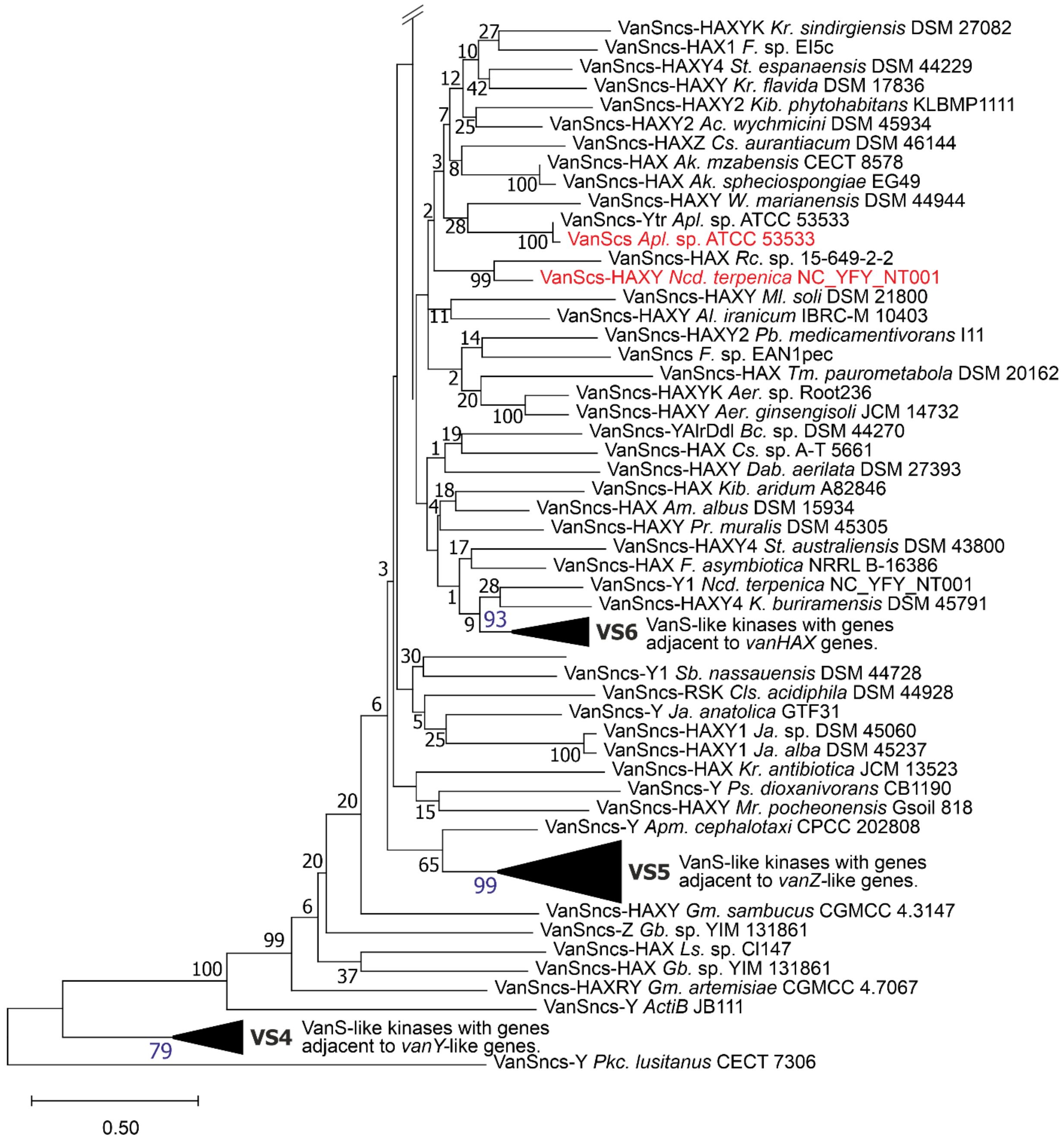

ESM Figure S25. Expanded version of the crown group of bacterial VanS-like sensor histidine kinases from tree in Figure 11b. Well-defined clusters VS4-8 were collapsed; their expanded versions are given in ESM Figures S27-31. Please refer to the main text for more details. BGC-encoded proteins are given in red. Scale bar represents number of substitutions per site.

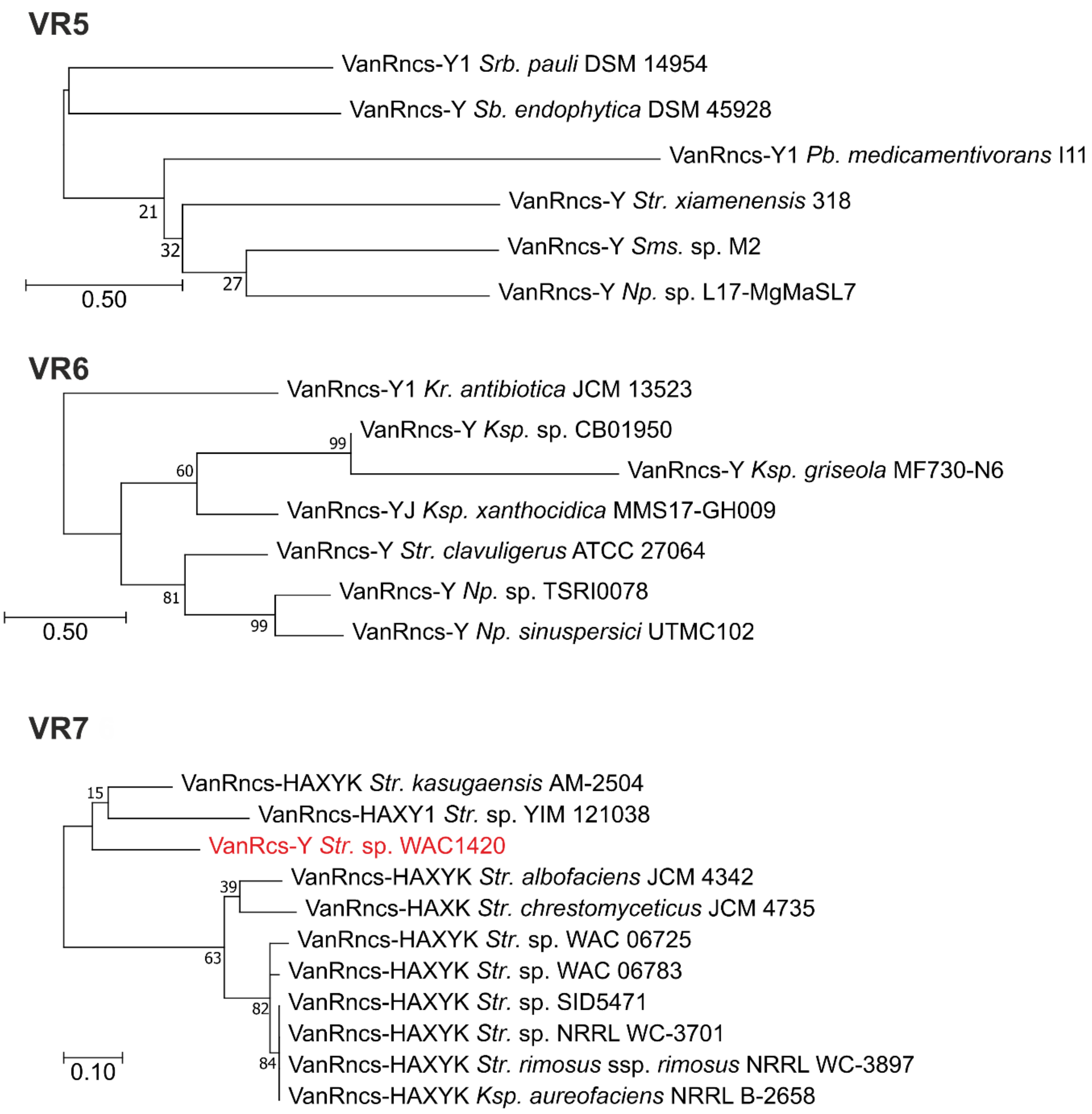

ESM Figure S26. Expanded versions of the VR5-7 clusters of the phylogenetic tree from ESM Figure S24. Please refer to the main text for more details. BGC-encoded proteins are given in red. Scale bar represents number of substitutions per site.

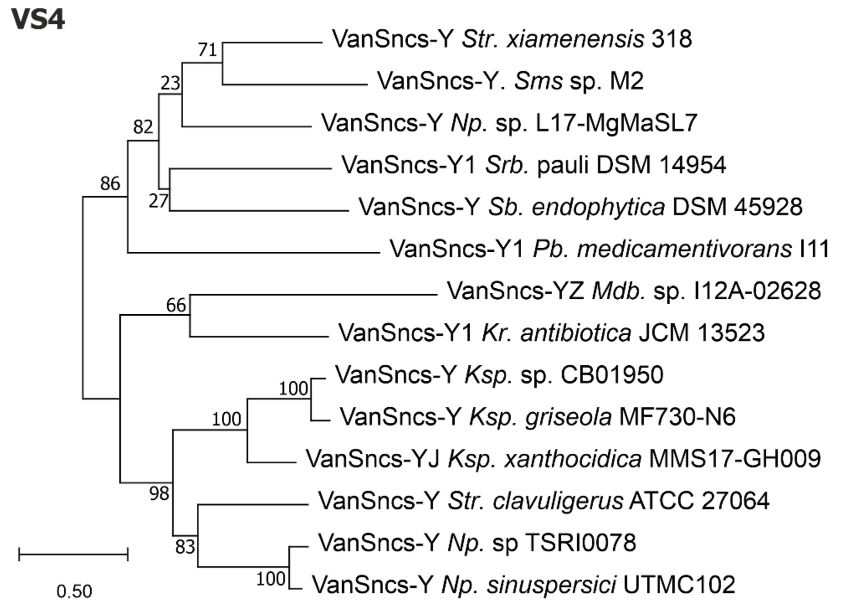

ESM Figure S27. Expanded version of the VS4 cluster of the phylogenetic tree from ESM Figure S25. Please refer to the main text for more details. Scale bar represents number of substitutions per site.

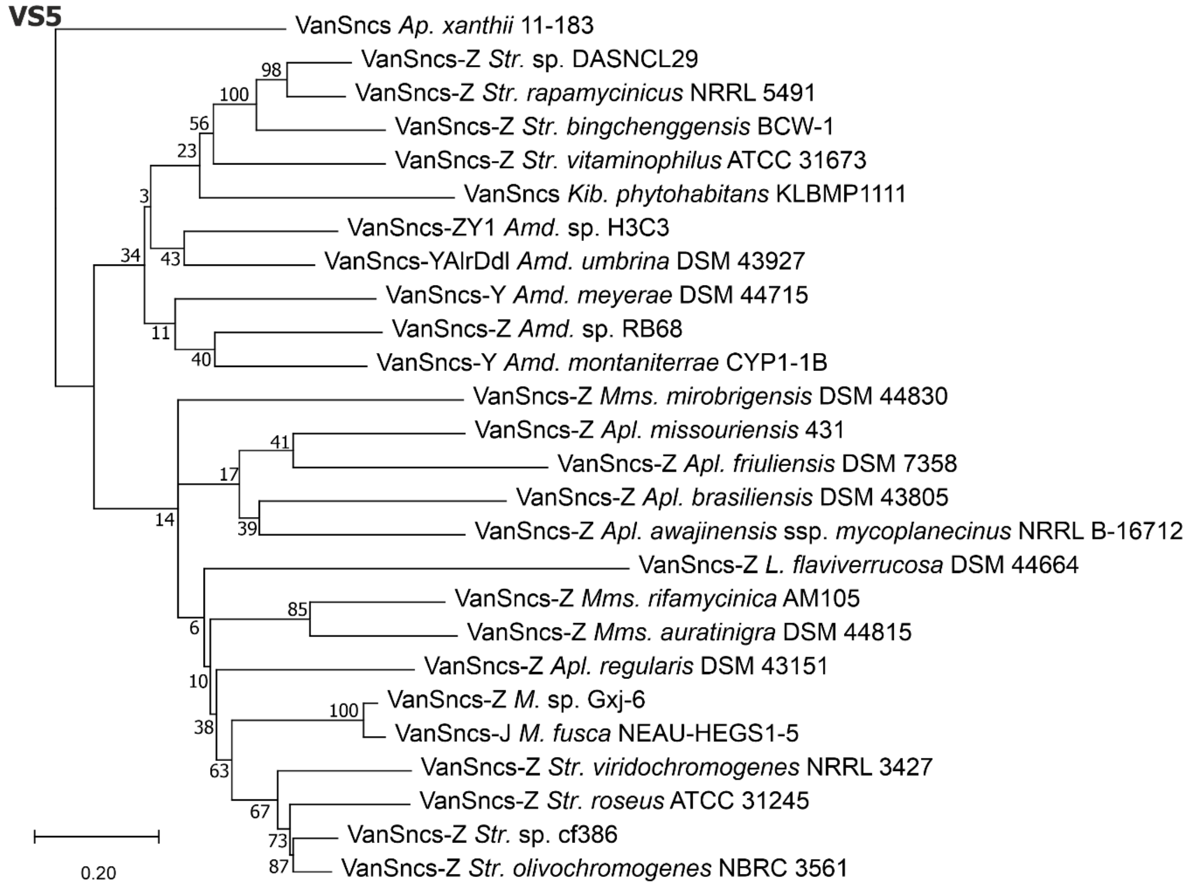

ESM Figure S28. Expanded version of the VS5 cluster of the phylogenetic tree from ESM Figure S25. Please refer to the main text for more details. Scale bar represents number of substitutions per site.

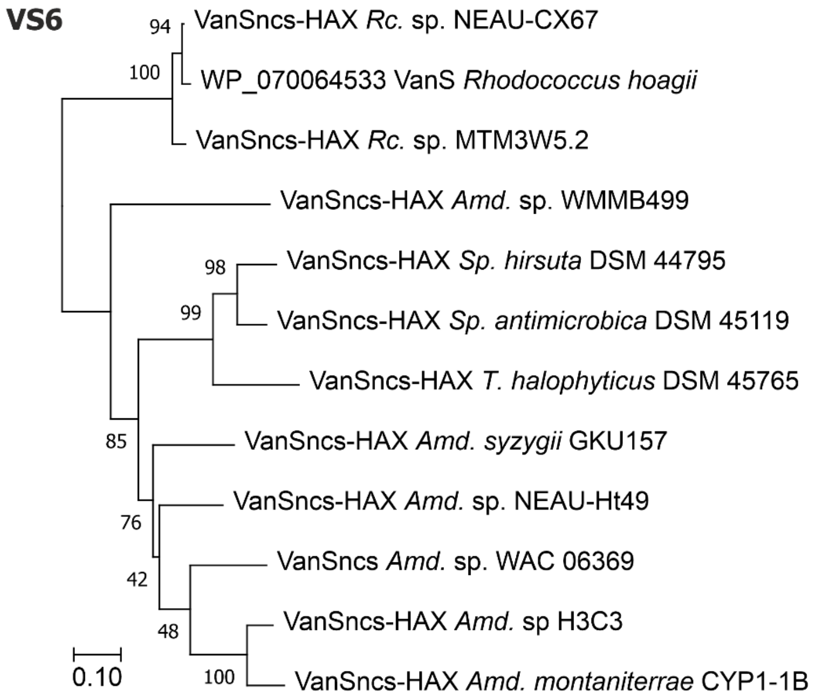

ESM Figure S29. Expanded version of the VS6 cluster of the phylogenetic tree from ESM Figure S25. Please refer to the main text for more details. Scale bar represents number of substitutions per site.

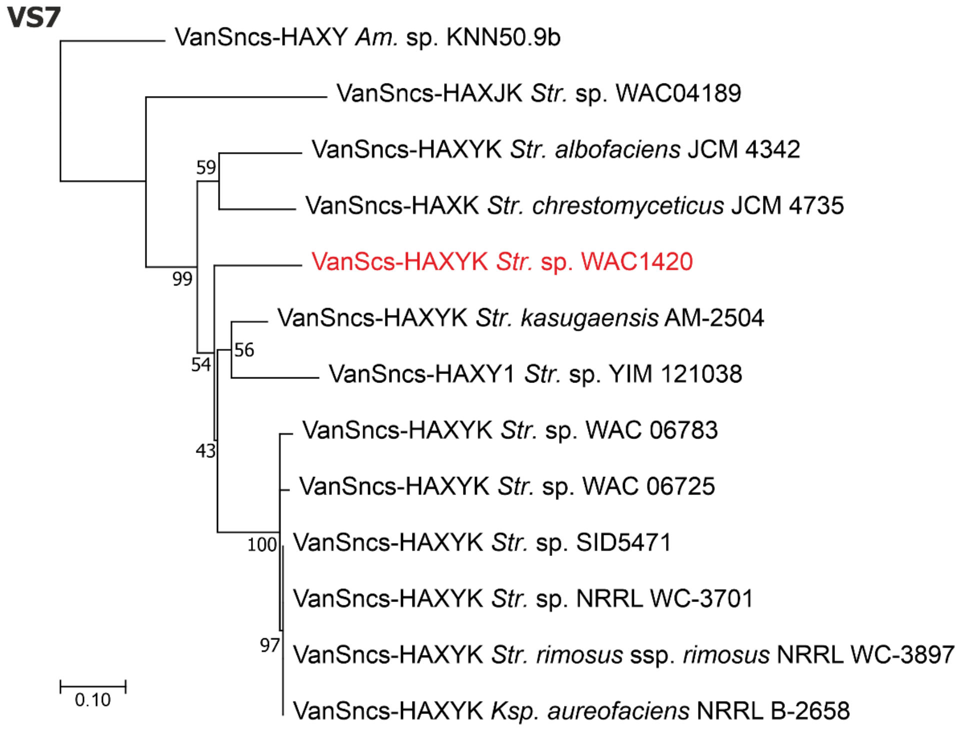

ESM Figure S30. Expanded version of the VS7 cluster of the phylogenetic tree from ESM Figure S25. Please refer to the main text for more details. BGC-encoded proteins are given in red. Scale bar represents number of substitutions per site.

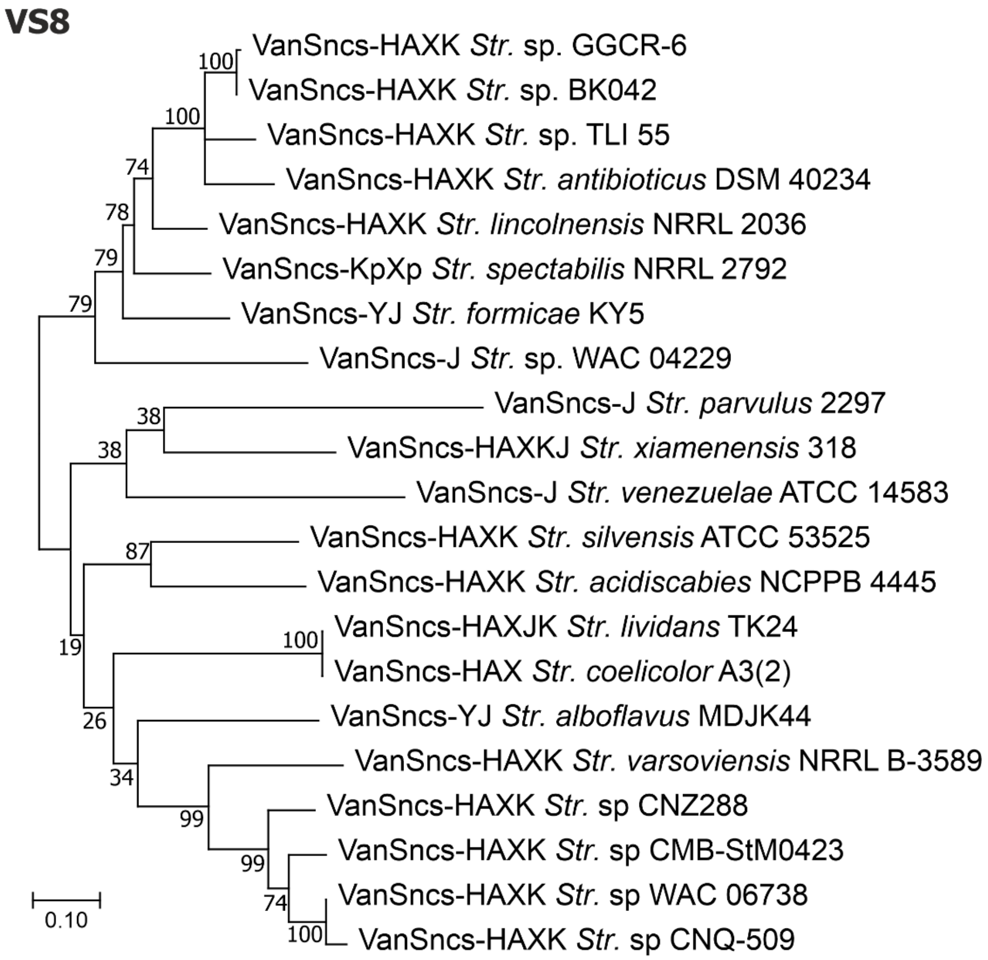

ESM Figure S31. Expanded version of the VS8 cluster of the phylogenetic tree from ESM Figure S25. Please refer to the main text for more details. Scale bar represents number of substitutions per site.

Supplementary references

1. Weigel, L.M.; Clewell, D.B.; Gill, S.R.; Clark, N.C.; McDougal, L.K.; Flannagan, S.E.; Kolonay, J.F.; Shetty, J.; Killgore, G.E.; Tenover, F.C. Genetic analysis of a high-level vancomycin-resistant isolate of *Staphylococcus aureus*. *Science*. **2003**, *302*, 1569–1571, doi:10.1126/science.1090956.
2. Heaton, M.P.; Discotto, L.F.; Pucci, M.J.; Handwerger, S. Mobilization of vancomycin resistance by transposon-mediated fusion of a VanA plasmid with an *Enterococcus faecium* sex pheromone-response plasmid. *Gene* **1996**, *171*, 9–17, doi:10.1016/0378-1119(96)00022-4.
3. Handwerger, S.; Skoble, J. Identification of chromosomal mobile element conferring high-level vancomycin resistance in *Enterococcus faecium*. *Antimicrob. Agents Chemother.* **1995**, *39*, 2446–2453, doi:10.1128/AAC.39.11.2446.
4. Paulsen, I.T.; Banerjei, L.; Hyers, G.S.A.; Nelson, K.E.; Seshadri, R.; Read, T.D.; Fouts, D.E.; Eisen, J.A.; Gill, S.R.; Heidelberg, J.F.; et al. Role of mobile DNA in the evolution of vancomycin-resistant *Enterococcus faecalis*. *Science*. **2003**, *299*, 2071–2074, doi:10.1126/science.1080613.
5. Garnier, F.; Taourit, S.; Glaser, P.; Courvalin, P.; Galimand, M. Characterization of transposon Tn1549, conferring VanB-type resistance in *Enterococcus* spp. *Microbiology* **2000**, *146*, 1481–1489, doi:10.1099/00221287-146-6-1481.
6. Boyd, D.A.; Cabral, T.; Van Caeseele, P.; Wylie, J.; Mulvey, M.R. Molecular characterization of the *vanE* gene cluster in vancomycin-resistant *Enterococcus faecalis* N00-410 isolated in Canada. *Antimicrob. Agents Chemother*. **2002**, *46*, 1977–1979, doi:10.1128/AAC.46.6.1977-1979.2002.
7. Quintiliani, R.; Courvalin, P. Characterization of Tn1547, a composite transposon flanked by the IS16 and IS256-like elements, that confers vancomycin resistance in *Enterococcus faecalis* BM4281. *Gene* **1996**, *172*, 1–8, doi:10.1016/0378-1119(96)00110-2.
